# Analysis of gene expression profile for identification of novel gene signatures during dengue infection

**DOI:** 10.1016/j.imj.2023.02.002

**Published:** 2023-02-18

**Authors:** Jhansi Venkata Nagamani Josyula, Prathima Talari, Agiesh Kumar Balakrishna Pillai, Srinivasa Rao Mutheneni

**Affiliations:** a Applied Biology Division, CSIR-Indian Institute of Chemical Technology, Tarnaka, Hyderabad, 500007, Telangana, India; b Academy of Scientific and Innovative Research (AcSIR), Ghaziabad 201002, Uttar Pradesh, India; c MGM Advanced Research Institute, Sri Balaji Vidyapeeth (Deemed to be University), Puducherry, 607402, India

**Keywords:** Dengue fever, Severe dengue, Microarray, Gene expression, Data analysis, Gene signatures

## Abstract

**Background:**

Dengue is a major arthropod-borne viral disease spreading rapidly across the globe. The absence of vaccines and inadequate vector control measures leads to further expansion of dengue in many regions globally. Hence, the identification of genes involved in the pathogenesis of dengue will help to understand the molecular basis of the disease and the genes responsible for the disease progression.

**Methods:**

In the present study, a meta-analysis was carried out using dengue gene expression data obtained from Gene Expression Omnibus repository. The differentially expressed genes such as CCNB1 and CCNB2 (G2/mitotic-specific cyclin-B2 and B1) were upregulated in dengue fever to control (DF-CO) and severe dengue (dengue hemorrhagic fever [DHF]) to control (DHF-CO) were identified as key genes for controlling the major pathways (cell cycle, oocyte meiosis, p53 signaling pathway, cellular senescence and progesterone-mediated oocyte maturation). Similarly, interferon alpha-inducible (IFI27) genes, type-I and type-III interferon (IFN) signaling genes (STAT1 and STAT2), B cell activation and survival genes (TNFSF13B, TNFRSF17) and toll like receptor (TLR7) genes were differentially up activated during DF-CO and DHF-CO. Followed by, Cytoscape was used to identify the immune system process and topological analysis.

**Results:**

The results showed that the top differentially expressed genes under the statistical significance *p* <0.001, which is majorly involved in Kyoto Encyclopedia of Genes and Genomes orthology K05868 and K21770 with gene names CCNB1 and CCNB2. In addition to this, the immune system profile showed up-regulation of IL12A, CXCR3, TNFSF13B, IFI27, TNFRSF17, STAT, STAT2, and TLR7 genes in DF-CO and DHF-CO act as immunological signatures for inducing the immune response towards dengue infection.

**Conclusions:**

The current study could aid in understanding of molecular pathogenesis, genes and corresponding pathway upon dengue infection, and could facilitate for identification of novel drug targets and prognostic markers.

## Highlights

• Two clinical forms of dengue and convalescent samples were tested for differential gene expression analysis using pair wise comparisons.
• Integrative omics reveals top gene signatures in transcriptomic profiles of dengue clinical forms.
• Gene ontology and Kyoto Encyclopedia of Genes and Genomes pathway was used for analysis of gene expression data.
• The immune genes (*IFI27, STAT1, STAT2, TLR7, TNFRSF17, TNFSF13B*) were recognized with 2-fold up-regulation in dengue infection.
• Ten regulatory proteins were identified which involves in pair comparisons by cytoHubba in cytoscape.

## 1 Introduction

Dengue is a major global public health concern infecting 50 to 100 million people annually [1]. The dengue virus (DENV) is transmitted to humans through the bite of infected female mosquito vectors *Aedes aegypti* and *Aedes albopictus*. The DENV belongs to the family *Flaviviridae*, genus *flavivirus* with 4 related but antigenically distinct serotypes ie, DENV-1, DENV-2, DENV-3 and DENV-4 [2], [3], [4]. The infection with any one of these serotypes ranges from a mild febrile illness (dengue fever [DF]) and occasionally develops into severe dengue (SD, also called dengue hemorrhagic fever) [5]. The clinical symptoms of DF include sudden onset of fever which may last for 2 to 7 days with intense headache, joint pains, muscle pains, vomiting, nausea and skin rash. Whereas SD develops potentially lethal complications due to plasma leakage, fluid accumulation, severe bleeding or organ impairment, respiratory distress, abdominal pain and fatigue [3,6].

Globally, dengue incidence has increased more than 8-fold during the past 2 decades. Dengue was more prevalent in South-East Asia, America, and Western Pacific regions. Asia currently bears around 70% of the world's dengue burden [1]. In recent years the dengue incidence has increased by over 400% in Asia and WHO estimated that there would be 100 million symptomatic cases and 300 million asymptomatic cases occurring annually [7]. The burden of dengue has increased due to a poor understanding of complex host-parasite interactions, lack of proper knowledge on host immune response towards disease outcome and severity [8], and lack of appropriate animal model studies [9]. However, in recent years efforts have been made by various researchers to understand the DENV pathogenesis and immune response such as T-cell activation, increased cytokine expression (including tumor necrosis factors [TNF], interleukin (IL)−1, IL-2 and IL-6, platelet-activating factor, complement components C3a and C5a and histamines) [10]. Risk factors for DF and SD were identified, but still, there is an uncertainty underlying understanding of the molecular mechanism of dengue pathogenesis [11].

Microarray technology is a novel approach to studying the differentially expressed genes (DEGs) during dengue infection and provides valuable insights, relationships and patterns between virus and host cell [12]. This technology has been used as a tool for identifying the signature genes in various diseases. Efforts have been made to find out the biomarkers associated with dengue and SD via microarray-based genome analysis of host gene expression patterns using human peripheral blood [13]. Nevertheless, none of the identified gene sets has yet been shown to be generalizable. Hence, the present study has focused on identification of gene expression signatures amongst different groups such as (1) DF and healthy controls (CO), (2) SD and healthy CO, (3) convalescent patients (CP) and DF, (4) CP and SD. Here, we have performed a meta-analysis of microarray data from a heterogeneous population consisting of DF, SD, convalescent subjects and healthy CO with a wide range of ages. The study findings can help to understand dengue pathogenesis and provide novel gene signatures for diagnostic and therapeutic intervention against the infection.

## 2 Materials and methods

### 2.1 Data analysis

"R" is a free statistical software and graphical programming language (version 3.6.0) which allows Bioconductor package (version 3.9) for microarray data analysis [14,15] to identify the DEGs between healthy subjects ie, CO, CP's, DF, and SD samples. Similarly, various statistical and data visualization packages of R software related to genomic analysis (limma, GenomicRanges and Rgraphviz) were downloaded and used for the analysis of the expression data. Heat maps of DEGs (up and down-regulated genes) between DF and healthy CO patients, SD and healthy CO patients, CP and DF, CP and SD were drawn using the heat map function in the R programme.

### 2.2 Microarray data

Microarray gene expression data was collected from the National Centre for Biotechnology Information Gene Expression Omnibus (GEO) database. These expression datasets were deposited from various experiments and users can enable to download the patterns stored in GEO [16]. To seek GEO datasets for related gene expression profiles, we have selected an array, which consists of all DF, SD, CP, and CO samples. However, the study found only one GEO data set that fulfils all search criteria containing the accession number GSE51808 [17]. The gene expression profiles of peripheral blood samples of 28 dengue patients, 19 CP, and 9 CO groups were obtained from the GEO database for further analysis [16]. The expression data was collected with the help of the GPL570 platform Affymetrix Human Genome HG-U133 plus 2.0 Array.

### 2.3 Data pre-processing & normalization

Microarray experiments produce large quantities of gene expression data hence, a systematic pre-processing is required to extract meaningful information from the data. Affy package in R was used for pre-processing the expression datasets [18]. During pre-processing the microarray image quality, probe signal intensity and background noise correction were assessed (Supplemental Fig. S1). Besides these, array-array intensity correlation (AAIC) analysis was also performed. The AAIC defines a symmetric square matrix of Spearman correlation (Supplemental Fig. S2) and the lowest correlation coefficient (*R* = 0.65) was observed between the 46th sample and other samples. Similarly, the quality of signal distribution and the quality of hybridization in an array were assessed by using a density plot (Supplemental Fig. S3) and an RNA degradation plot (Supplemental Fig. S4).

The normalization of data includes (1) log-transformation, (2) missing value management, (3) flat pattern filtering and (4) pattern standardization were executed. The expression data were normalized using robust multichip averaging (RMA) method. RMA is the most widely used data pre-processing algorithm to perform background correction using log transformation, and data normalization through Quantile normalization (QN) [19], [20], [21]. Further, the normalized data were analyzed using principal component analysis for outlier (sample) detection and removal of batch effect on expression data.

### 2.4 Microarray data analysis

Differential expression analysis was performed using the Limma package in R. The Limma uses a linear modeling approach to estimate the feature dependencies between samples and variability in the data set. This analysis determines gene expression patterns which are significantly up-or-down regulated during DF, SD, CP and healthy samples. The DEGs were identified using the selection criteria of an empirical Bayes moderated *t*-test and statistically significant genes were identified with |log^FC(fold change)^| > 2 and <−2 with an adjusted *p* value *(p < 0.001*) based on the false discovery rate using the Benjamini-Hochberg (BH) method [22]. The volcano plots were drawn using R (ggplot2 and Venn diagram packages) to display up-regulated and down-regulated DEGs.

#### 2.4.1 Gene ontology and pathway enrichment analysis

Gene Ontology (GO) and Kyoto Encyclopedia of Genes and Genomes (KEGG) pathway enrichment analysis was performed. The GO analysis provides defined GO terms which cover cellular components, molecular functions, biological processes and pathway annotations [23], [24], [25], [26], [27]. Furthermore, pathway enrichment analyses (*p* < 0.05 was considered for significant enrichment) also performed using the clusterProfiler package in R.

#### 2.4.2 Gene set enrichment analysis

Gene set enrichment analysis (GSEA) is a powerful analytical tool for interpreting the results of gene expression data at the level of the gene set. GSEA was used to evaluate a specific gene set that is the genes group that has common biological functions, pathways and relations between genes thus reducing the dimensionality of the genomic landscape [28]. Different signature gene (C1–C7) sets (c7.all.v7.4.entrez.gmt) were downloaded from the Molecular Signature Database (MSigDb) and used as a reference gene set for identification of molecular signatures from the annotated gene sets [28]. The GSEA analysis was performed using the ClusterProfiler package in R.

#### 2.4.3 Protein-protein interaction analysis

The search tool for the retrieval of interacting genes (STRING), a database for the prediction of known and unknown protein functional interactions was employed to construct protein-protein interactions (PPIs) at the transcriptional and translational level of proteins encoded by DEGs [28]. The medium confidence interaction score >0.4 in STRING was considered, which infers that interactions with a medium level of confidence were extracted from the database to construct PPI network. Cytoscape software (version 3.8.2) was used to visualize the PPI network of up-regulated and down-regulated DEGs [29].

Furthermore, to understand the functions of DEGs we performed functional enrichment analysis using ClueGO, a Cytoscape plug-in. The statistical test used for ClueGO enrichment analysis was based on a 2-sided hypergeometric test (*p* ≤ 0.05) with a Benjamini-Hochberg correction and kappa score ≥0.4 as a primary criterion [30].

## 3 Results

**Fig. 1:**
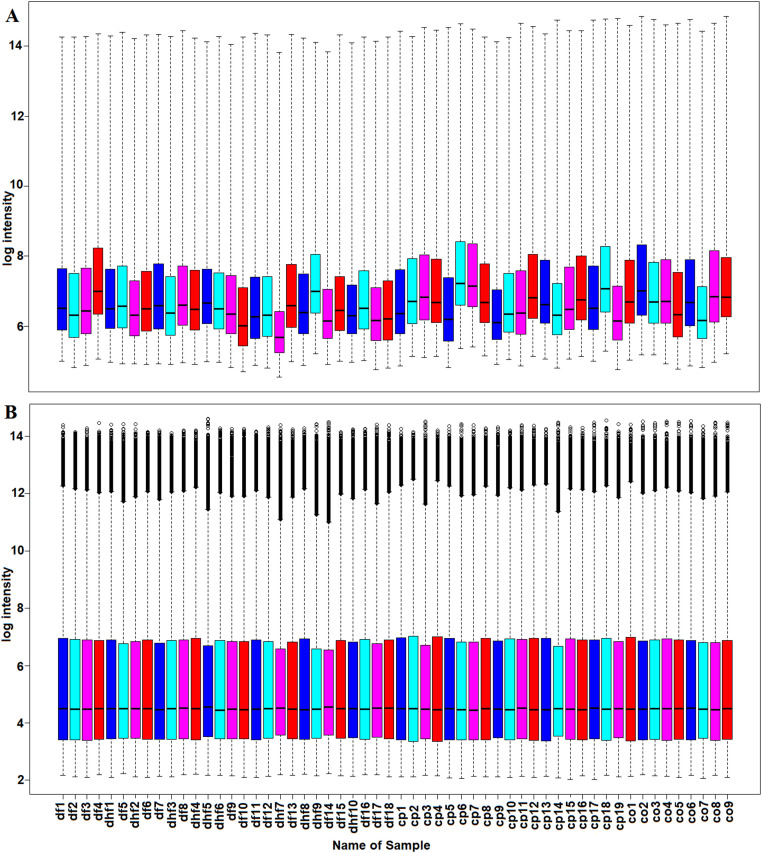
Box plots for gene expression data of each sample (A) before normalization (B) after normalization of the dengue samples.

The gene expression data set GSE51808 was selected and obtained from the GEO database. The GSE51808 expression data consist of 56 peripheral blood samples (Supplemental Table S1) including 28 dengue patients (13 from DF and 6 from SD), 19 CP and 9 healthy CO. After normalization, a total of 54,175 top genes were identified in the array (Fig. 1). Further, the normalized data were processed for principal component analysis demonstrating that there was a clear distinction between DF, SD, CP, and CO samples. The principal components (PC) PC1 captured 32.21% and PC2 captured 9.94% of the variance (Supplemental Fig. S5 A). The scree plot shows that the PC explain 80% proportion of variance cumulatively at PC23 (Supplemental Fig. S5 B).

### 3.1 Differentially expressed genes

**Fig. 2:**
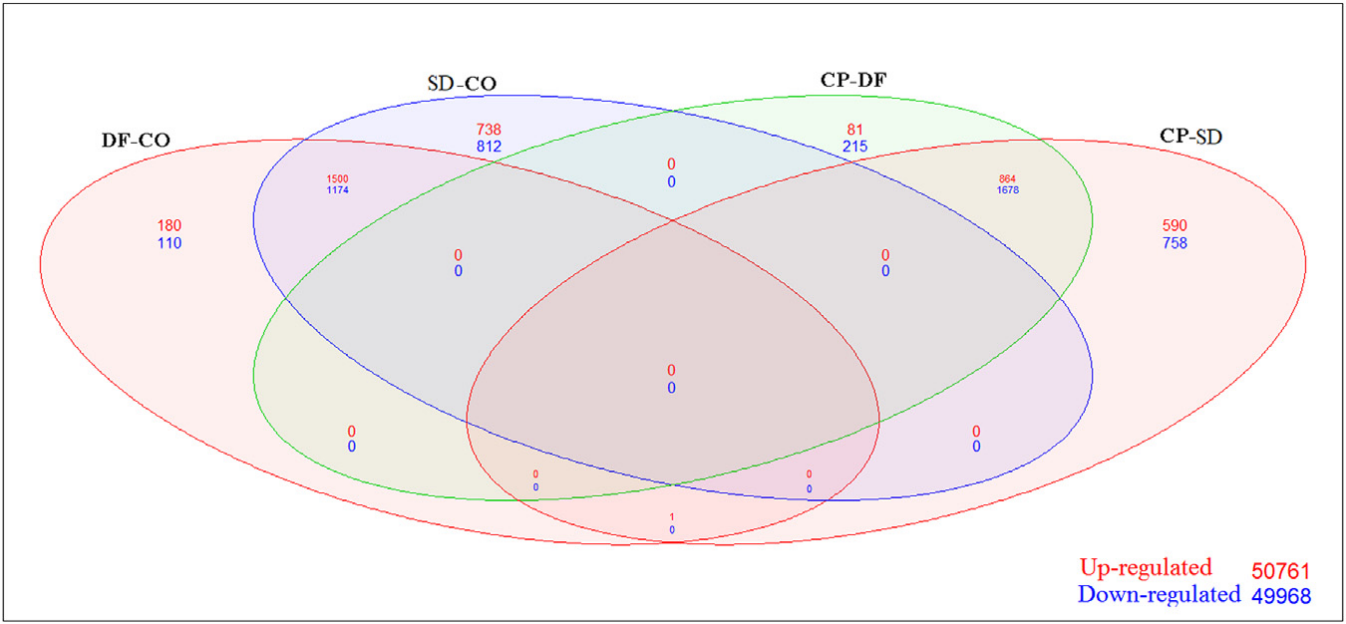
Venn showed (up and down) regulation of genes between the groups (DF-CO, SD-CO, CP-DF, and CP-DHF). CP, convalescent patients; DF-CO, dengue fever to control; DHF, dengue hemorrhagic fever; SD, severe dengue.

**Fig. 3:**
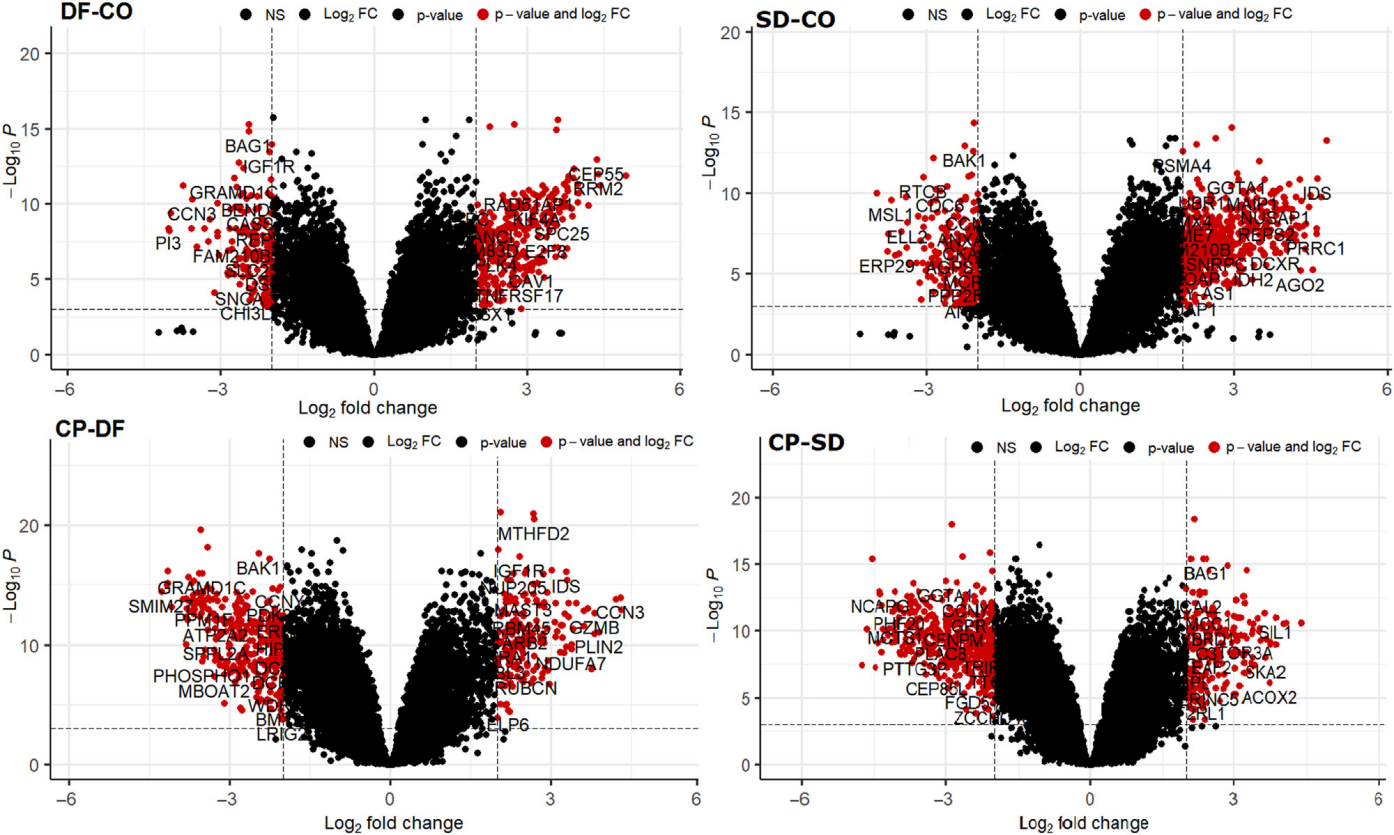
Volcano plots demonstrating an overview of DEGs. The plot compared the DEGs between DF-CO, SD-CO, CP-DF, and CP-SD groups. The down-regulated genes are on the left side of the plot (0–6) and up-regulated are on the right side of the plot (0–6). CP, convalescent patients; DEGs, differentially expressed genes; DF-CO, dengue fever to control; SD, severe dengue.

**Fig. 4:**
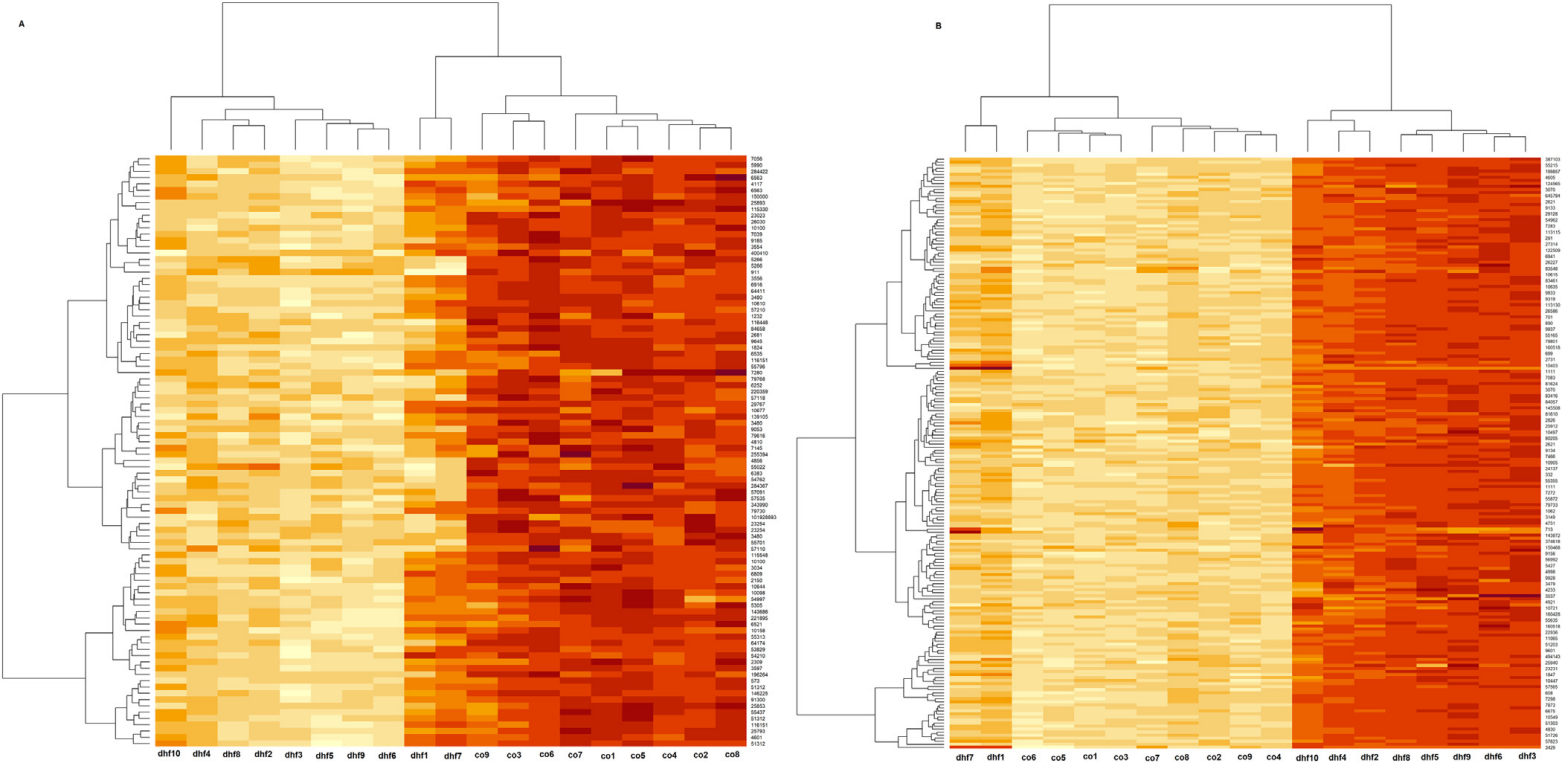
Hierarchical cluster analysis of top 200 DEGs (up-regulated and down-regulated) between severe dengue and control groups. Hierarchical cluster analysis between other clinical groups was shown in Supplemental Figure S6 (A-F). DEGs, differentially expressed genes.

The DEGs were recognized using fold change analysis (Limma package) with |Log FC| ≥ 2 and |Log FC| ≤ 2 with adjusted *p<0.001* (Table 1). A total of 5606 DEGs in DF-CO (324 up-regulated and 165 downregulated), 6209 genes in SD-CO (470 up, 228 down), 8528 genes in CP-DF (182 up, 306 down), and 8163 genes in the CP-SD group (213 up, 476 down) were identified. In Figure 2, the Venn diagram explains the distribution of DEGs across the 4 groups. There was one common up-regulated gene between the CP-SD and DF-CO, 1500 up-regulated and 1174 down-regulated genes were observed between DF-CO and SD-CO and 864 up-regulated and 1678 down-regulated were observed between CP-DF and CP-SD respectively (Fig. 2). The volcano plot (Fig. 3) displayed the DEGs between DF-CO, SD-CO, CP-DF and CP-SD with up and down-regulation with an adjusted *p<0.001* and log FC of −2 to −4 and +2 to +4 were selected for further analysis. The top 200 DEGs were subjected to a heatmap to illustrate the expression levels of the genes across samples using the Hierarchical clustering algorithm shown in Figure 4 and Supplemental Figure S6 (A-F).

**Table 1:** Differentially expressed genes (up and down regulated) with 4-fold log2 change in paired groups.

	Probe-ID	Gene symbol	Log FC	Average expression	T	*p*-value	Adjusted *p*-value	Regulation
DF-CO	41469_at	PI3	−4.02046	7.153617	−8.18953	3.24E-11	4.42E-09	Down
	218542_at	CEP55	4.359249	6.154028	11.912	3.96E-17	1.14E-13	Up
	201890_at	RRM2	4.395685	8.834722	11.19507	4.84E-16	1.02E-12	Up
	202411_at	IFI27	4.930528	10.74717	11.09436	6.92E-16	1.36E-12	Up
	209642_at	BUB1	4.008007	6.311865	10.09827	2.51E-14	1.83E-11	Up
SD-CO	201890_at	RRM2	4.628832	8.834722	10.47468	6.38E-15	1.21E-11	Up
	218542_at	CEP55	4.281092	6.154028	10.39433	8.54E-15	1.42E-11	Up
	202589_at	TYMS	4.070686	9.036458	10.145	2.12E-14	2.63E-11	Up
	209642_at	BUB1	4.213311	6.311865	9.432154	2.95E-13	1.66E-10	Up
	202411_at	IFI27	4.708685	10.74717	9.414068	3.16E-13	1.73E-10	Up
	203764_at	DLGAP5	4.313627	6.838005	9.228543	6.32E-13	2.88E-10	Up
	226661_at	CDCA2	4.059122	5.651605	8.797882	3.2E-12	9.27E-10	Up
	219148_at	PBK	4.130931	5.632338	8.762545	3.66E-12	1.02E-09	Up
	219493_at	SHCBP1	4.133453	7.042283	8.338829	1.83E-11	3.25E-09	Up
	212097_at	CAV1	4.405742	6.241624	8.035951	5.84E-11	8.19E-09	Up
	223565_at	MZB1	4.616763	9.093747	7.838319	1.25E-10	1.46E-08	Up
CP-DF	235683_at	SESN3	4.303916	9.263779	12.09622	2.1E-17	1.09E-14	Up
	241881_at	TRIM58	4.211268	6.808613	11.95262	3.45E-17	1.58E-14	Up
	218542_at	CEP55	−4.15205	6.154028	−14.0823	3.03E-20	7.2E-17	Down
	201890_at	RRM2	−4.14821	8.834722	−13.1129	6.93E-19	7.6E-16	Down
	209642_at	BUB1	−4.16669	6.311865	−13.0301	9.11E-19	9.23E-16	Down
	202411_at	IFI27	−4.08248	10.74717	−11.4017	2.34E-16	7.31E-14	Down
CP-SD	235683_at	SESN3	4.071956	9.263779	9.635058	1.39E-13	2.56E-11	Up
	241881_at	TRIM58	4.021634	6.808613	9.609884	1.52E-13	2.72E-11	Up
	201890_at	RRM2	−4.38136	8.834722	−11.6603	9.47E-17	1.1E-13	Down
	218542_at	CEP55	−4.07389	6.154028	−11.6328	1.04E-16	1.14E-13	Down
	209642_at	BUB1	−4.37199	6.311865	−11.5107	1.6E-16	1.59E-13	Down
	203764_at	DLGAP5	−4.11815	6.838005	−10.3616	9.61E-15	3.54E-12	Down
	219148_at	PBK	−4.00006	5.632338	−9.97888	3.89E-14	9.56E-12	Down
	219493_at	SHCBP1	−4.05841	7.042283	−9.62899	1.42E-13	2.6E-11	Down
	223565_at	MZB1	−4.64471	9.093747	−9.27421	5.33E-13	7.2E-11	Down
	212097_at	CAV1	−4.24828	6.241624	−9.11307	9.75E-13	1.11E-10	Down
	206641_at	TNFRSF17	−4.13187	8.990867	−8.51095	9.52E-12	6.69E-10	Down

CP, convalescent patients; DF-CO, dengue fever to control; SD, severe dengue.

### 3.2 GO enrichment and KEGG pathway analysis

**Fig. 5:**
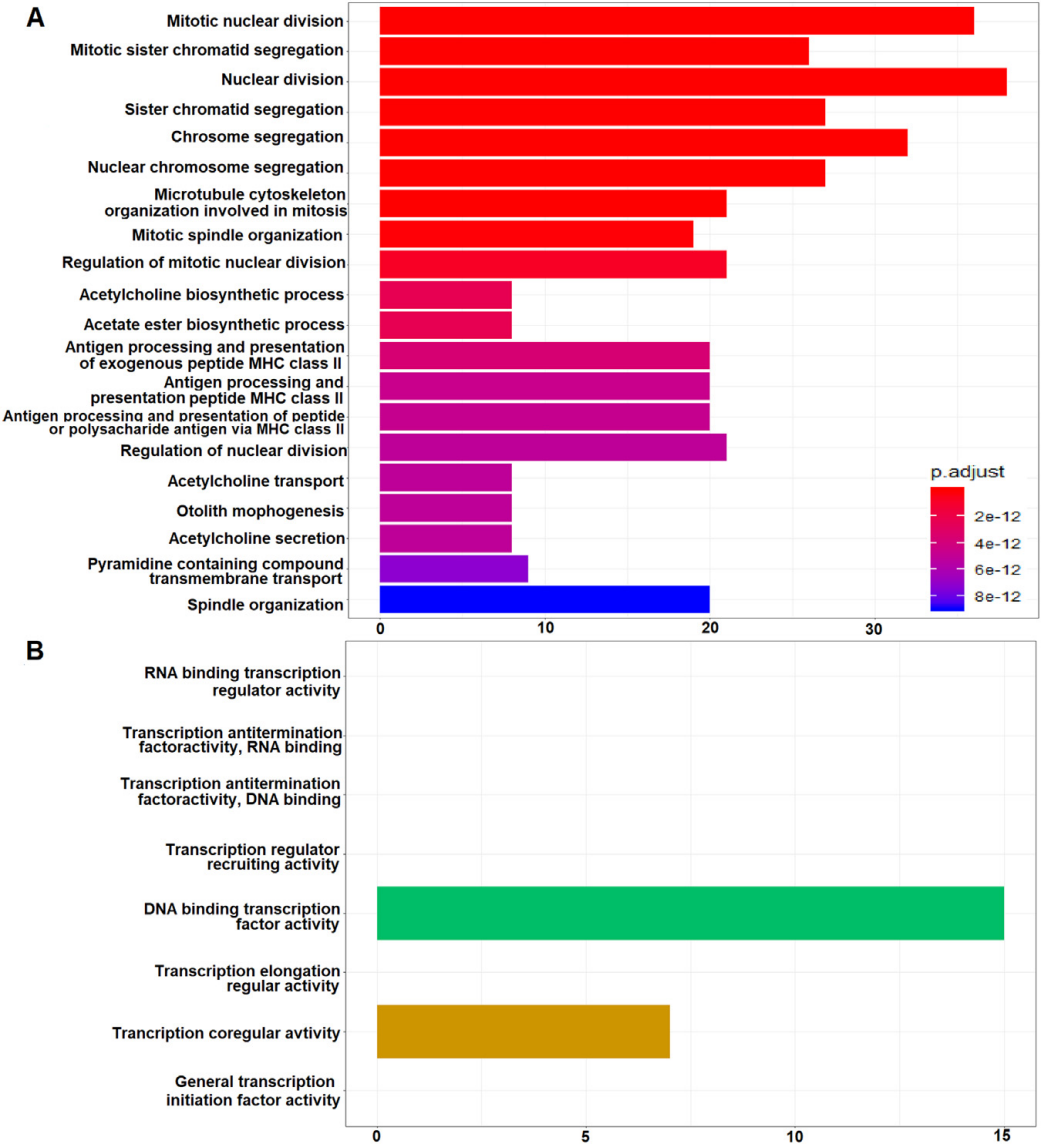
Gene ontology (GO) enrichment analysis showing most enriched GO terms are biological processes and molecular function of SD-CO (other groups are shown in Supplemental Figs. S7 A-M). The x-axis represents the number of DEGs enriched terms. Y-axis represents the GO terms. CO, control; DEGs, differentially expressed genes. SD, severe dengue.

The GO functional enrichment analysis of DEGs was significantly enriched (*P<0.05* with a 2-fold increase or decrease log fold change) in cellular functions, biological processes and molecular functions (Supplemental Table S2, Fig. 5, and Supplemental Figure S7 [A-M]). The GO exploration has shown that a significant variation in differentially expressed transcripts across 4 groups was shown in the network plot (Supplemental Fig. S8). The GO analysis revealed that the genes *BUB1, RRM2, IFI27, DLGAP5,* and *CEP55* exhibit 4-fold up-regulation in dengue and SD patients however, they exhibit downregulation in CP (Supplemental Fig. S8). In addition, KEGG pathway analysis explored up and down-regulation of DEGs which were highly associated with various metabolic pathways mentioned in Table 2.

**Table 2:** KEGG pathways enriched in DEGs of 4 groups enriched in pathways with significant regulation using *p-value*.

ID	Description	Gene ratio	*p*-value	Gene ratio	*p*-value	Gene ratio	*p*-value	Gene ratio	*p*-value
		DF-CO		SD-CO		CP-DF		CP-SD	
hsa04110	Cell cycle	16↑	2.50E-15	15↑	6.54E-11	15↓	8.75E-14	16↓	5.24E-12
hsa04115	p53 signaling pathway	8↑	1.40E-07	8↑	4.99E-06	8↓	1.76E-07	9↓	4.50E-07
hsa04218	Cellular senescence	9↑	5.34E-06	10↑	3.99E-05	9↓	6.83E-06	10↓	4.00E-05
hsa04114	Oocyte meiosis	8↑	1.09E-05	8↑	0.000306	6↓	0.000821	7↓	0.001615
hsa04914	Progesterone-mediated oocyte maturation	7↑	1.84E-05	8↑	5.14E-05	6↓	0.000209	7↓	0.000354
hsa05166	Human T-cell leukemia virus 1 infection	8↑	0.000455693	–	–	7↓	0.00269	–	–
hsa00240	Pyrimidine metabolism	4↑	0.001195692	–	–	4↓	0.001333	–	–
hsa05161	Hepatitis B	6↑	0.002279132	–	–	–	–	–	–
hsa04152	AMPK signaling pathway	5↓	0.003233464	–	–	–	–	–	–
hsa03030	DNA replication	3↑	0.003301914	–	–	5↓	1.27E-05	5↓	0.000104
hsa05144	Malaria	–	–	5↓	0.000502	–	–	–	–
hsa01521	EGFR tyrosine kinase inhibitor resistance	–	–	6↑↓	0.000613	–	–	–	–
hsa01523	Antifolate resistance	–	–	–	–	3↓	0.002327	–	–
hsa00670	One carbon pool by folate	–	–	–	–	3↓	0.000632	–	–
hsa04141	Protein processing in endoplasmic reticulum	–	–	–	–	–	–	10↓	8.72E-05

CO, control; CP, convalescent patients; DEGs, differentially expressed genes; DF, dengue fever, DF-CO, dengue fever to control; KEGG, Kyoto Encyclopedia of Genes and Genomes; SD, severe dengue. (↓Downregulation; ↑Up regulation).

The metabolic pathways involved in 4 groups (DF-CO, SD-CO, CP-DF, CP-SD) were cell cycle, p53 signaling (hsa04115), cellular senescence (hsa04218), oocyte meiosis (hsa04114) and progesterone-mediated oocyte maturation (hsa04914). These metabolic pathways showed upregulation in DF-CO and SD-CO and it is inversely proportional in CP-DF and CP-SD. Similarly, the AMPK signaling pathway (hsa04152) was downregulated only in DF-CO condition (Table 2).

### 3.3 Identification of molecular signature genes

The GSEA was performed using the molecular signatures database (MSigDB). The MSigDB organizes into 8 major collections (https://www.gsea-msigdb.org/gsea/msigdb/) of human genes assembled based on their location. In DEGs, molecular signatures were identified from 8 collections (Hallmark + C1 to C7) presented in Supplemental Table S3.

### 3.4 PPI networks

**Fig. 6:**
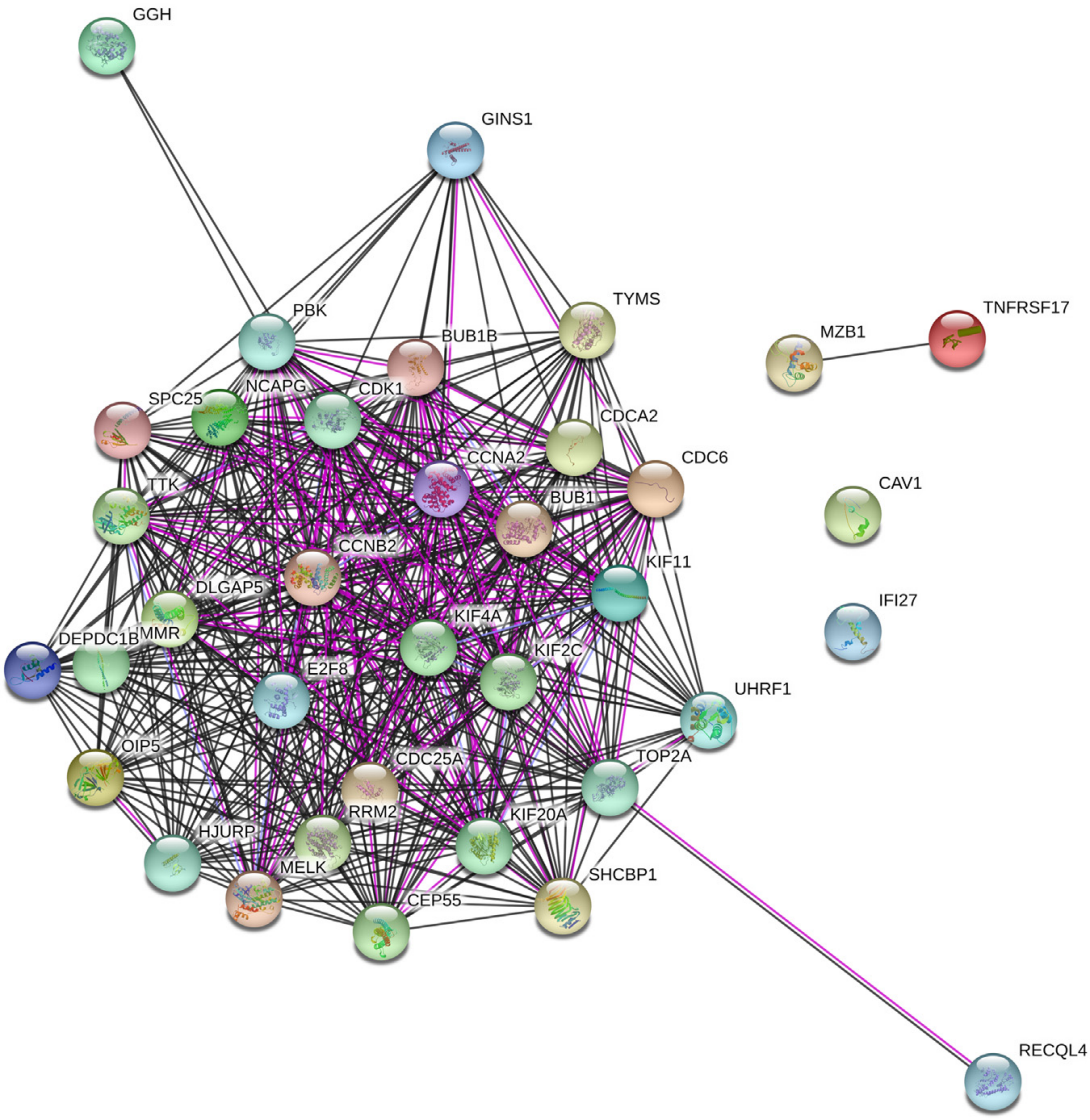
STRING generated interaction network between commonly identified up and down-regulated DEGs genes in 4 group comparisons. STRING, Search Tool for the Retrieval of Interacting Genes.

The physical and functional associations of proteins of DEGs were evaluated using the STRING tool and visualized the network by using Cytoscape software. A total of 516 nodes (DF-CO:145; SD-CO: 20; CP-DF:148; CP-SD:203 nodes), and 8213 edges (DF-CO:2533; SD-CO: 285; CP-DF:2357; CP-SD:3038 edges) were identified in the network with interaction score of >0.4 (Fig.-6). Nodes indicate the number of proteins and edges signify their interaction. The PPI network of DF-CO; SD-CO; CP-DF; CP-SD are shown in the supplementary document (Supplemental Figs. S9-S12). The accuracy of the PPI network was assessed by clustering coefficient, network density and PPI enrichment *p-*values mentioned in Table 3 and Supplemental Table-S4. Cytoscape network contains CytoHubba for ranking nodes in a network based on network features to infer their importance in the network. Based on available genes in the network further, we have searched for connected hub genes using 5 topological analysis methods such as Degree, Edge Percolated Component, EcCentricity, Maximal Clique Centrality, and Maximum Neighborhood Component in the CytoHubba. The top 10 genes which were recognized by Cytohubba in different groups are presented in Supplemental Table S5.

**Table 3:** Cytoscape interaction network analysis of DEGs-related proteins.

Summary statistics	DF-CO	SD-CO	CP-DF	CP-SD
Number of nodes	145	20	148	203
Number of edges	2533	285	2357	3038
Average number of neighbors	34.938	27.689	31.851	29.931
Network diameter	5	7	5	8
Network radious	1	1	1	1
Characteristic path length	1.458	1.89	1..680	1.938
Clustering coefficient	0.304	0.258	0.29	0.272
Network density	0.121	0.068	0.108	0.074
Connected components	4	3	2	2
Multiedge node pairs	0	0	0	0
Number of self-loops	0	0	0	0
PPI enrichment *p*-value:	< 1.0e-16	< 1.0e-16	< 1.0e-16	< 1.0e-16

CO, control; CP, convalescent patients; DEGs, differentially expressed genes; DF, dengue fever, DF-CO, dengue fever to control; PPIs, protein-protein interactions; SD, severe dengue.

**Fig. 7:**
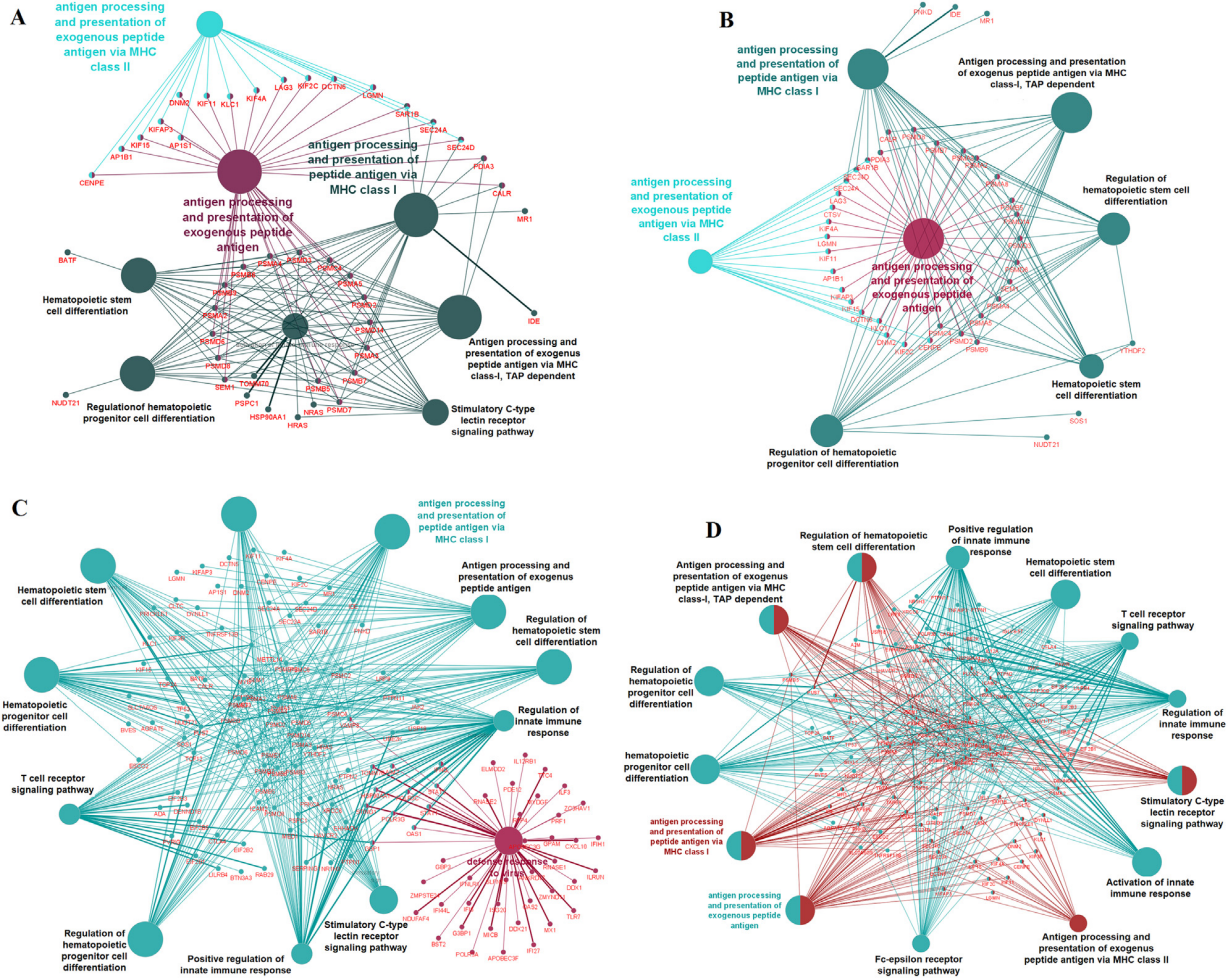
Cytoscape immune response pathway network of significantly over-represented Immune system process gene ontology transcriptome and proteome profiling by ClueGo for DEGs. DEGs, differentially expressed genes.

Furthermore, the Cytoscape plugin ClueGO/CluePedia is also used to study the functional enrichment of DEGs. ClueGo helped to visualize the GO terms of the immune system process identified in the PPI network. The DEGs from the PPI network (immune system process) were predominantly enriched for up-regulation of antigen processing and presentation of exogenous peptide antigen (GO:0002478), antigen processing and presentation of exogenous peptide antigen via major histocompatibility complex (MHC) class II (GO:0019886), antigen processing and presentation of peptide antigen via MHC class I (GO:0002474), antigen processing and presentation of exogenous peptide antigen via MHC class I, transporter associated with antigen processing (TAP) dependent (GO:0002479), hematopoietic stem cell differentiation (GO:0060218), regulation of hematopoietic progenitor cell differentiation (GO:1901532) and regulation of hematopoietic stem cell differentiation (GO:1902036) in DF-CO, SD-CO and it is indirectly proportional to the CP-DF and CP-SD. Hemopoiesis (GO:0030097) and myeloid cell differentiation (GO:0030099) were downregulated in DF-CO and SD-CO (Fig. 7; Supplemental Table S6).

## 4 Discussion

The present study aimed to investigate the DEGs between dengue patients and healthy CO samples. The data (GSE51808) was curated and removed the background noise, annotated and summarized the probes. Following this, the data were normalized using RMA and downstream analyses to identify the biologically significant DEGs. This analysis explores expression variability amongst 56 samples which helps us to identify strategies to clearly understand dengue pathology and to find the genes which are easily accessible to promote early detection of dengue. A total of 5606 DEGs were observed in DF-CO, 6209 genes in SD-CO, 8528 genes in CP-DF samples, and 8163 genes in CP-SD samples.

MHC class I polypeptide-related sequence B (MICB) is an important gene associated with dengue infection [31]. The MICB encodes an activating ligand for natural killer cells and possibly CD8+ T lymphocytes. Mutations in MICB would collapse the anti-viral effector functions in NK cells which lead to higher DENV infection, which is a known risk factor for SD [31,32]. In the present study, the MICB was up-regulated in both DF-CO and SD-CO conditions with a fold change of 0.9. similarly, the CP-DF and CP-SD have shown downregulation with a fold change of 0.1. Generally, the toll-like receptors (TLR) play an important role in pathogen recognition and activation of inflammatory pathways during dengue infection [33]. In the present study, the expression of the TLR6 gene was decreased in DF-CO, and SD-CO with a fold change of −1.325 and −1.422 respectively. Similarly, an increased expression of the TLR7 gene was observed with a fold change of 1.3 in both DF-CO and SD-CO conditions. TLR7 is an endosomal pattern-recognition receptor for single-stranded RNA viruses and it also controls the host immunological response to infections by recognizing the viral uridine-containing single-strand RNAs [34].

In the immune response category, an up-regulated gene expression of tumor necrosis factor receptor TNFSF13B with a fold change of 1.27, 1.39 and downregulation of other receptors like TNFRSF10B*,* C and 14 was observed in DF-CO, SD-CO. Moreover, the TNFSF13B gene is associated with B cell activation and also showed an immune response to live attenuated tetravalent dengue vaccine candidates [35]. Following this, TNFRSF17 plays an important role in the control of humoral immunity and promotes B-cell survival which was upregulated with 3.8, 2.8-fold change in SD-CO and DF-CO conditions [36]. Similarly, a cluster of genes consisting of nuclear factor 1A, 1B, and 1C expression was down-regulated in DF-CO and SD-CO conditions.

SD illness requires the activation of multiple inflammatory pathways. It is observed that an up-regulation of interferon-gamma (IFN), IL12A in SD-CO samples with an increased fold change of 1.94397 whereas, a significant downregulation was noticed in CP-SD condition [37]. It is also observed that an up-regulated gene expression of Interleukin enhancer-binding factor 3-A (ILF3) contributes an innate immunity by participating in cellular antiviral responses and also interacts with the viral NS3 protein [38,39]. The interleukin receptors such as IL11RA, IL13RA1, IL1R1, IL1RAP, IL6R, and IL7R have shown downregulation during dengue infection. Here, it is worth mentioning that IL12RB2 (Interleukin-12 receptor subunit beta-2) gene expression was observed in DF-CO, and SD-CO conditions at a fold change of 0.7352 and 1.2 respectively whereas, the similar expression was not observed in SD-CO and CP-SD conditions.

Similarly, the chemokine receptors such as CCR10, CCR3, CCR6, CXCR3, and CXCR4 showed both up and down-regulated gene expressions in healthy CO, convalescent, DF and SD samples. The humoral immune response genes C2, CXCR3, IRF4 and POU2AF1 expression are up-regulated in DF-CO, SD-CO and down-regulated in CP-DF and CP-SD conditions. The CXCR3 plays a protective role in dengue infection however the absence of this significantly damages the host's defense against viral infections [40]. POU2AF1 is a B-cell transcriptional coactivator and IRF4 is associated with the activation of T cells [41]. Similarly, the gene expression of CD1C, ITGB2, PTAFR, SFTPD, and YY1 are downregulated in DF-CO and SD-CO conditions.

The interferon genes that have been identified as part of the type I IFN profile STAT1, STAT2, OAS2, and IFI27 gene expressions were up-regulated in DF-CO and an inverse relationship was noted in SD-CO conditions. However, earlier studies have reported that the expression of these genes was up-regulated in DF patients and down-regulated in SD patients [9,42]. Similarly, IFI27 is a mitochondrial protein that contributed to IFN-induced apoptosis by disrupting normal mitochondrial activity [42]. The CRTAP gene was downregulated in the present study and it plays an important role in cell junction integrity (cell-cell adhesion) and collagen assembly (an extracellular matrix component) [43]. The platelet-related genes IL1R1, IL13RA1, and IL6R have phosphorylation sites found in human cells. Moreover, platelet-related genes CRTAP and IL11RA had no phosphorylation sites was observed.

The cell cycle transcription factors such as repressor genes E2F6, E2F7, and E2F8 are located on chromosome 7 and responsible for cell cycle regulation. The expression of the above genes was up-regulated with a fold change of 3 in DF-CO, and SD-CO conditions but, CP-DF, and CP-SD expressed inversely. Similarly, E2F1 the cell cycle activator was up-regulated with a fold change of 0.487. Furthermore, tyrosine-protein phosphatases (PTPN1, PTPN2) showed increased gene expression and PTPRC, PTPRJ and PTPRO showed downregulation in DF-CO and SD-CP conditions. The molecular function of GO analysis showed that cyclin-dependant protein serine/threonine kinase regulator activity (GO:0016538) was observed in DF-CO and CP-SD conditions. Similarly, the protein kinase regulator activity (GO:0019887) was observed only in DF-CO samples. Hydroxymethyl-, formyl- and related transferase activity (GO:0016742) was perceived in CP-DF and catalytic activity (GO:0140097) was observed in CP-SD samples.

The ClueGO enrichment analysis showed that the DEGs alter the behavior of the immune system and are closely associated with the up-regulation of antigen processing and immune response to the virus, which can lead to activation of cellular immune response by MHC class I and class II-restricted cell surface expression towards DENV in DF-CO and SD-CO [44]. CytoHubba network analysis showed topmost intersecting genes derived from 4 pair comparisons using Maximal Clique Centrality are AURKB, DLGAP5, RRM2, KIF11, BUB1B, CCNB2, MELK, BIRC5, BUB1, and PBK. These are the core proteins and key candidate genes which have importance in biological regulatory functions.

## 5 Conclusions

Our study sheds some light on the molecular underpinnings of inflammatory gene expression patterns in peripheral blood mononuclear cells. This analysis represented DEGs with statistical significance less than 0.001 (adjusted *p* value) which are majorly involved in metabolic KEGG orthology K05868 and K21770 with gene names CCNB1 and CCNB2. The immunological profile showed an upregulation of IL12A, CXCR3, TNFSF13B, IFI27, TNFRSF17, STAT, STAT2, and TLR7 genes in DF-CO and SD-CO for simulation of the immune response towards dengue infection. This OMICS analysis revealed the systems-level top gene signatures in the transcriptomic profile of dengue clinical forms. The outcome of the study assists us in learning more about how dengue comprehends the downstream target gene molecules and their signaling pathways. Further transcriptional profiling of dengue samples will reveal the interesting functional mechanisms of disease pathogenesis. The major limitation is, the study has considered only a single microarray dataset of dengue clinical conditions which may not provide adequate information. However, including multiple microarray datasets provides a larger volume of the sample size which increases the accuracy and novelty of identifying the gene-related markers.

## Funding

The authors do not receive any funding for this study.

## Author contributions

Execution of research method, bioinformatics analysis, result interpretation, experimental validation, and original draft preparation were performed by Jhansi Venkata Nagamani Josyula. Prathima Talari has done the collection of data, pre-processing, and bioinformatics analysis. Conceptualization, supervision, reviewing, by Srinivasa Rao Mutheneni and final editing of the manuscript were accomplished by Agiesh Kumar Balakrishna Pillai and Srinivasa Rao Mutheneni.

## Acknowledgments

The authors are grateful to the Director of the Council of Scientific and Industrial Research-Indian Institute of Chemical Technology (CSIR-IICT), Hyderabad, for his encouragement and support. Jhansi Venkata Nagamani Josyula acknowledges ICMR for funding the ICMR-SRF fellowship. The funders had no role in the study design, data collection, analysis, decision to publish, or preparation of the manuscript. CSIR-IICT communication number of the article is IICT/Pubs./2021/258.

## Declaration of competing interest

The authors declare no conflict of interest exists.

## Data available statement

The data were available at the National Centre for Biotechnology Information (NCBI) Gene Expression Omnibus (GEO) database under accession number GSE51808.

## Ethics statement

The gene expression data were downloaded from the NCBI GEO public database, and there were no animal or human experiments carried out by any of the authors.

## Informed consent

Not applicable.

## Appendix Supplementary materials

Image, application 1

Image, application 2
