# Supplementary material for: Analysis of gene expression profile for identification of novel gene signatures during dengue infection
Source: Infect Med (Beijing). 2023 Feb 18;2(1):19–30. doi: 10.1016/j.imj.2023.02.002 (PMC10699721; doi:10.1016/j.imj.2023.02.002)
Supplement: Supplementary file 1 [file mmc1.docx]

| **Group** | **Accession** | **Title** | **Organism** | **Infection** | **Status** | **Tissue** |
| --- | --- | --- | --- | --- | --- | --- |
| Dengue Fever  (DF) | GSM1253028 | Patient 3 | Homo Sapiens | DENV | DF | Whole blood |
|  | GSM1253033 | Patient 33 | Homo Sapiens | DENV | DF | Whole blood |
|  | GSM1253035 | Patient 35 | Homo Sapiens | DENV | DF | Whole blood |
|  | GSM1253036 | Patient 36 | Homo Sapiens | DENV | DF | Whole blood |
|  | GSM1253038 | Patient 37 | Homo Sapiens | DENV | DF | Whole blood |
|  | GSM1253029 | Patient 4 | Homo Sapiens | DENV | DF | Whole blood |
|  | GSM1253042 | Patient 41 | Homo Sapiens | DENV | DF | Whole blood |
|  | GSM1253043 | Patient 42 | Homo Sapiens | DENV | DF | Whole blood |
|  | GSM1253044 | Patient 43 | Homo Sapiens | DENV | DF | Whole blood |
|  | GSM1253045 | Patient 44 | Homo Sapiens | DENV | DF | Whole blood |
|  | GSM1253047 | Patient 47 | Homo Sapiens | DENV | DF | Whole blood |
|  | GSM1253050 | Patient 54 | Homo Sapiens | DENV | DF | Whole blood |
|  | GSM1253051 | Patient 55 | Homo Sapiens | DENV | DF | Whole blood |
|  | GSM1253030 | Patient 6 | Homo Sapiens | DENV | DF | Whole blood |
|  | GSM1253053 | Patient 66 | Homo Sapiens | DENV | DF | Whole blood |
|  | GSM1253054 | Patient 68 | Homo Sapiens | DENV | DF | Whole blood |
|  | GSM1253031 | Patient 7 | Homo Sapiens | DENV | DF | Whole blood |
|  | GSM1253055 | Patient 80 | Homo Sapiens | DENV | DF | Whole blood |
| Severe Dengue  (SD) | GSM1253032 | Patient 32 | Homo Sapiens | DENV | SD | Whole blood |
|  | GSM1253034 | Patient 34 | Homo Sapiens | DENV | SD | Whole blood |
|  | GSM1253039 | Patient 38 | Homo Sapiens | DENV | SD | Whole blood |
|  | GSM1253040 | Patient 39 | Homo Sapiens | DENV | SD | Whole blood |
|  | GSM1253041 | Patient 40 | Homo Sapiens | DENV | SD | Whole blood |
|  | GSM1253046 | Patient 45 | Homo Sapiens | DENV | SD | Whole blood |
|  | GSM1253048 | Patient 48 | Homo Sapiens | DENV | SD | Whole blood |
|  | GSM1253049 | Patient 49 | Homo Sapiens | DENV | SD | Whole blood |
|  | GSM1253052 | Patient 60 | Homo Sapiens | DENV | SD | Whole blood |
|  | GSM1253037 | Patient 81 | Homo Sapiens | DENV | SD | Whole blood |
| Convalescent Patients | GSM1253056 | Patient 3 | Homo Sapiens | DENV | Convalescent | Whole blood |
|  | GSM1253059 | Patient 33 | Homo Sapiens | DENV | Convalescent | Whole blood |
|  | GSM1253060 | Patient 34 | Homo Sapiens | DENV | Convalescent | Whole blood |
|  | GSM1253061 | Patient 35 | Homo Sapiens | DENV | Convalescent | Whole blood |
|  | GSM1253062 | Patient 36 | Homo Sapiens | DENV | Convalescent | Whole blood |
|  | GSM1253063 | Patient 37 | Homo Sapiens | DENV | Convalescent | Whole blood |
|  | GSM1253064 | Patient 38 | Homo Sapiens | DENV | Convalescent | Whole blood |
|  | GSM1253065 | Patient 39 | Homo Sapiens | DENV | Convalescent | Whole blood |
|  | GSM1253057 | Patient 4 | Homo Sapiens | DENV | Convalescent | Whole blood |
|  | GSM1253066 | Patient 41 | Homo Sapiens | DENV | Convalescent | Whole blood |
|  | GSM1253067 | Patient 44 | Homo Sapiens | DENV | Convalescent | Whole blood |
|  | GSM1253068 | Patient 45 | Homo Sapiens | DENV | Convalescent | Whole blood |
|  | GSM1253069 | Patient 47 | Homo Sapiens | DENV | Convalescent | Whole blood |
|  | GSM1253070 | Patient 48 | Homo Sapiens | DENV | Convalescent | Whole blood |
|  | GSM1253071 | Patient 49 | Homo Sapiens | DENV | Convalescent | Whole blood |
|  | GSM1253072 | Patient 54 | Homo Sapiens | DENV | Convalescent | Whole blood |
|  | GSM1253073 | Patient 55 | Homo Sapiens | DENV | Convalescent | Whole blood |
|  | GSM1253058 | Patient 6 | Homo Sapiens | DENV | Convalescent | Whole blood |
|  | GSM1253074 | Patient 80 | Homo Sapiens | DENV | Convalescent | Whole blood |
| Control | GSM1253079 | Control c1 | Homo Sapiens | Control | Control | Whole blood |
|  | GSM1253083 | Control c2 | Homo Sapiens | Control | Control | Whole blood |
|  | GSM1253075 | Control c3 | Homo Sapiens | Control | Control | Whole blood |
|  | GSM1253077 | Control c4 | Homo Sapiens | Control | Control | Whole blood |
|  | GSM1253076 | Control c5 | Homo Sapiens | Control | Control | Whole blood |
|  | GSM1253078 | Control c6 | Homo Sapiens | Control | Control | Whole blood |
|  | GSM1253081 | Control c7 | Homo Sapiens | Control | Control | Whole blood |
|  | GSM1253080 | Control c8 | Homo Sapiens | Control | Control | Whole blood |
|  | GSM1253082 | Control c9 | Homo Sapiens | Control | Control | Whole blood |

Table-S1: Information on dengue fever, severe dengue, convalescent patients and control features in GSE51808 from the GEO database.

| **ID** | **Description** | **Gene ratio** | **P-value** | **Gene ratio** | **P-value** | **Gene ratio** | **P-value** | **Gene ratio** | **P-value** |
| --- | --- | --- | --- | --- | --- | --- | --- | --- | --- |
| **Molecular function** | | **DF-CO** | | **SD-CO** | | **CP-DF** | | **CP-SD** | |
| GO:0003777 | Microtubule motor activity | 10/193 | 5.50522E-09 | 9/253 | 2.33E-06 | 10/187 | 4.0615E-09 | 10/250 | 2.09906E-07 |
| GO:0003774 | Motor activity | 10/193 | 8.19049E-07 | 11/253 | 2.77E-06 | 10/187 | 6.1342E-07 | 11/250 | 2.46694E-06 |
| GO:0008017 | Microtubule binding | 15/193 | 6.27292E-08 | 15/253 | 1.51E-06 | 14/187 | 2.7537E-07 | 15/250 | 1.29948E-06 |
| GO:0015631 | Tubulin binding | 17/193 | 1.256968E-07 | 16/253 | 1.36E-05 | 16/187 | 4.3844E-07 | 16/250 | 1.1725E-05 |
| GO:0035173 | Histone kinase activity | 4/193 | 1.61076E-05 | 4/253 | 5.51E-05 | 4/187 | 1.4225E-05 | 4/250 | 5.26235E-05 |
| GO:0016538 | Cyclin-dependent protein serine / threonine kinase regulator activity | 6/193 | 7.02597E-06 | NA | NA | NA | NA | 6/250 | 3.45569E-05 |
| GO:0019887 | Protein kinase regulator activity | 9/193 | 0.000125106 | NA | NA | NA | NA | NA | NA |
| GO:0016742 | Hydroxymethyl-, formyl- and related transferase activity | NA | NA | NA | NA | 3/187 | 0.00011486 | NA | NA |
| GO:0140097 | Catalytic activity, acting on DNA | NA | NA | NA | NA | NA | NA | 11/250 | 9.4342E-05 |
| **Cellular component** | |  |  |  |  |  |  |  |  |
| GO:0098687 | Chromosomal region | 29/198 | 2.01186E-18 | 30/267 | 1.08E-15 | 29/193 | 9.7904E-19 | 31/265 | 1.12249E-16 |
| GO:0005819 | Spindle | 29/198 | 3.26697E-18 | 29/267 | 3.09E-15 | 24/193 | 1.1526E-13 | 26/265 | 9.49008E-13 |
| GO:0000775 | Chromosome, centromeric region | 24/198 | 1.44746E-19 | 26/267 | 1.22E-17 | 24/193 | 7.8534E-20 | 25/265 | 1.0178E-17 |
| GO:0000793 | Condensed chromosome | 24/198 | 9.93838E-19 | 26/267 | 9.75E-18 | 24/193 | 5.4138E-19 | 27/265 | 6.83753E-19 |
| GO:0000779 | Condensed chromosome, centromeric region | 20/198 | 4.24423E-19 | 21/267 | 5.49E-18 | 20/193 | 2.539E-19 | 21/265 | 4.70047E-18 |
| GO:0000776 | Kinetochore | 20/198 | 4.46889E-18 | 21/267 | 1.06E-16 | 20/193 | 2.6828E-18 | 21/265 | 9.08983E-17 |
| GO:0005874 | Microtubule | 20/198 | 1.02146E-08 | 21/267 | 2.14E-07 | 19/193 | 3.6267E-08 | 21/265 | 1.88886E-07 |
| GO:0000777 | Condensed chromosome kinetochore | 18/198 | 1.68035E-17 | 19/267 | 1.87E-16 | 18/193 | 1.0603E-17 | 19/265 | 1.62496E-16 |
| GO:0072686 | Mitotic spindle | 13/198 | 1.05194E-09 | 12/267 | 4.34E-08 | 8/193 | 5.4785E-05 | 9/265 | 2.17E-05 |
| GO:0030496 | Midbody | 13/198 | 1.78109E-08 | 12/267 | 2.19E-06 | 11/193 | 9.4273E-07 | 12/265 | 2.02683E-06 |
| GO:0000922 | Spindle pole | 12/198 | 5.79306E-08 | 13/267 | 2.08E-07 | 9/193 | 2.2943E-05 | 10/265 | 4.94097E-05 |
| GO:0005875 | Microtubule associated complex | 11/198 | 4.21294E-07 | 12/267 | 8.65E-07 | 10/193 | 2.6883E-06 | 12/265 | 7.99047E-07 |
| GO:0005876 | Spindle microtubule | 10/198 | 5.42393E-10 | 10/267 | 3.43E-09 | 9/193 | 8.4639E-09 | 10/265 | 3.19538E-09 |
| **Biological process** | |  |  |  |  |  |  |  |  |
| GO:0007059 | Chromosome segregation | 37/193 | 1.684E-28 | 38/261 | 6.69E-25 | 37/189 | 7.5685E-29 | 39/257 | 3.17E-26 |
| GO:0000280 | Nuclear division | 33/193 | 6.02293E-20 | 36/261 | 9.04E-19 | 35/189 | 2.8022E-22 | 38/257 | 7.90E-21 |
| GO:0140014 | Mitotic nuclear division | 29/193 | 4.8273E-21 | 32/261 | 4.27E-21 | 31/189 | 1.3177E-23 | 33/257 | 2.27E-22 |
| GO:0098813 | Nuclear chromosome segregation | 28/193 | 8.45693E-21 | 29/261 | 2.32E-18 | 29/189 | 3.275E-22 | 30/257 | 1.42E-19 |
| GO:0000819 | Sister chromatid segregation | 24/193 | 4.49334E-20 | 25/261 | 3.48E-18 | 26/189 | 8.0697E-23 | 27/257 | 1.27E-20 |
| GO:0000070 | Mitotic sister chromatid segregation | 23/193 | 1.53082E-20 | 24/261 | 3.87E-19 | 25/189 | 2.1052E-23 | 25/257 | 1.67E-20 |
| GO:0007051 | Spindle organization | 18/193 | 1.0865E-12 | 18/261 | 1.1E-10 | 19/189 | 6.3612E-14 | 18/257 | 8.52E-11 |
| GO:0044843 | Cell cycle G1/S phase transition | 18/193 | 2.03695E-09 | 17/261 | 6.98E-07 | 18/189 | 1.4536E-09 | 17/257 | 5.63E-07 |
| GO:0010948 | Negative regulation of cell cycle process | 18/193 | 8.55134E-08 | 20/261 | 5.26E-07 | 19/189 | 1.1015E-08 | 20/257 | 4.12E-07 |
| GO:0045787 | Positive regulation of cell cycle | 18/193 | 1.05337E-07 | 23/261 | 5.92E-09 | 18/189 | 7.6512E-08 | 18/257 | 1.16E-07 |
| GO:0006959 | Humoral immune response | 18/193 | 1.92755E-07 | no | no | no | no | no | no |
| GO:1902850 | Microtubule cytoskeleton organization  involved in mitosis | 17/193 | 1.63609E-13 | 17/261 | 7.91E-12 | 18/189 | 7.7521E-15 | 17/257 | 6.18E-12 |
| GO:0006260 | DNA replication | 17/193 | 1.41487E-09 | 18/261 | 3.48E-08 | 20/189 | 1.6418E-12 | 23/257 | 2.13E-12 |
| GO:0090068 | Positive regulation of cell cycle process | 17/193 | 7.93473E-09 | 21/261 | 8.43E-10 | 18/189 | 7.6512E-08 | 18/257 | 1.16E-07 |

Table-S2: Gene set enrichment analysis of differentially expressed genes involved in Biological function, Cellular component and Molecular function (with enriched *P<0.05*).

| **Data sets** | **ID** | **Description** | **GENE ID** | **p-value** | | | |
| --- | --- | --- | --- | --- | --- | --- | --- |
|  |  |  |  | **DF-CO** | **SD-CO** | **CP-DF** | **CP-SD** |
| **Hall**  **mark** | HALLMARK_  E2F_TARGETS | Genes encoding cell cycle related targets of E2F transcription factors. | 6241/9787/3161/57405/7153/  9133/983/11004/1111/9833/  701/79733/24137/9212/332/9837/9319 | 7.50E-32 | 0.001217 | 0.001342 | 9.11E-23 |
|  | HALLMARK_  G2M_CHECKPOINT | Genes involved in the G2/M checkpoint, as in progression through the cell division cycle. | 699/55872/3161/7153/9133/983/  990/11004/7272/1111/890/3832/  24137/56992/9212//993/11065 | 2.13E-17 | 0.001241 | 0.001364 | 4.75E-15 |
|  | HALLMARK_  MITOTIC_SPINDLE | Genes important for mitotic spindle assembly. | 699/9787/7153/9133/983/11004/  7272/3832/24137/56992/332/4751/9700 | 6.16E-06 | 0.001272 | 0.001418 | 6.46E-05 |
|  | HALLMARK_  HEME_METABOLISM | Genes involved in metabolism of heme (a cofactor consisting of iron and porphyrin) and erythroblast differentiation. | 129642/23608/25893/645/3920/  51094/4601/25793/51312/6535/7145/6521/10158 | 1.80E-03 | 0.004237 | 0.003509 | 2.23E-09 |
| **C1** | chr1q23 | Genes in Cytogenetic Band chr1q23 | 5498/83540/83416/57823/6675/4921/911 | …....... | 0.00026 | …....... | ….... |
| **C2** | KOBAYASHI_EGFR_  SIGNALING_24HR_DN | Genes down-regulated in H1975 cells (non-small cell lung cancer, NSCLC) resistant to gefitinib [PubChem=123631] after treatment with EGFR inhibitor CL-387785 [PubChem=2776] for 24h. | 6241/55165/699/9787/7298/79801/  55872/3161/64151/57405/7153/  983/990/11004/7272 | 9.25E-73 | 5.06E-69 | 8.98E-65 | 1.74E-66 |
|  | LEE_EARLY_T_  LYMPHOCYTE_UP | Genes up-regulated at early stages of progenitor T lymphocyte maturation compared to the late stages. | 6241/55165/699/9787/7298/55872/  157313/3161/51237/64151/7153/  9133/983/11004/7272/29128/9833 | 1.20E-66 | 4.55E-65 | 4.47E-55 | 8.17E-60 |
|  | GRAHAM_NORMAL_  QUIESCENT_VS_  NORMAL_DIVIDING_DN | Genes down-regulated in quiescent vs dividing CD34+ [GeneID=8842] cells isolated from peripheral blood of normal donors. | 6241/55165/699/9787/7298/64151/  7153/9133/983/990/11004  /1111/9833/701/890 | 9.29E-49 | 1.93E-43 | 3.57E-47 | 1.35E-45 |
|  | HORIUCHI_WTAP_  TARGETS_DN | Genes down-regulated in primary endothelial cells (HUVEC) after knockdown of WTAP [GeneID=9589] by RNAi | 6241/55165/699/9787/7298  /55872/157313/3161/64151  /57405/7153/9133/983/11004/7272 | 1.53E-50 | 3.11E-45 | 2.93E-42 | 7.00E-38 |
|  | ZHOU_CELL_CYCLE  _GENES_IN_IR_  RESPONSE_24HR | Cell cycle genes significantly (p =< 0.05) changed in fibroblast cells at 24 h after exposure to ionizing radiation. | 55165/699/9787/3161/57405/  7153/9133/983/7272/29128/  9833/79733/890/3832 | 1.74E-39 | 1.08E-37 | 1.99E-36 | 4.96E-29 |
|  | HOFFMANN_LARGE_  TO_SMALL_  PRE_BII_  LYMPHOCYTE_UP | Genes up-regulated during differentiation from large pre-BII to small pre-BII lymphocyte. | 6241/699/9787/7153/9133/  983/11004/7272/9833/79733  /113130/890/10112/24137/55355/9212 | 2.78E-33 | 9.57E-29 | 5.60E-29 | 5.22E-20 |
|  | KEGG_  CELL_CYCLE | Cell cycle | 699/9133/983/990/7272/1111  /701/890/4173/9700/993/  4998/9134/891/5111/898 | 4.84E-15 | 1.22E-11 | 1.23E-12 | 2.79E-15 |
|  | REACTOME_CELL_  CYCLE_CHECKPOINTS | Cell Cycle Checkpoints | 699/57405/9133/983/990/11004/  1111/701/890/9212/332/  4173/993/11065/83540 | 1.06E-19 | 4.99E-17 | 1.39E-16 | 6.47E-13 |
| **C3** | E2F_Q6_01 | Genes having at least one occurence of the transcription factor binding site in the regions spanning up to 4 kb around their transcription starting sites. | 6241/990/9833/79733/144455/  4173/5427/993/4998/10733/  54962/5111/9185 | 2.18E-07 | 1.33E-05 | 4.78E-08 | 8.79E-08 |
|  | E2F1_Q3 | Genes having at least one occurence of the transcription factor binding site in the regions spanning up to 4 kb around their transcription starting sites. | 6241/983/990/79733/144455/  56992/5427/993/4998/10733/  54962/63967/5111 | 2.90E-07 | 1.73E-05 | 6.44E-06 | 1.05E-06 |
|  | E2F_Q3 | transcription factor targets | 983/990/79733/144455/56992/  4173/5427/993/4998/10733/54962/5111 | 8.12E-07 | 3.52E-05 | 8.95E-06 | 6.77E-05 |
|  | E2F1_Q6 | Genes having at least one occurence of the transcription factor binding site | 6241/983/990/79733/144455/  4173/5427/993/4998/63967/5111 | 7.68E-06 | 2.19E-04 | 5.05E-05 | 1.82E-04 |
|  | E2F_Q4_01 | transcription factor targets | 990/9833/79733/144455/4173/  5427/993/4998/10733/54962/5111 | 8.33E-06 | 2.36E-04 | 5.24E-05 | 9.29E-06 |
|  | E2F_Q6 | transcription factor targets | 6241/983/990/79733/144455  /4173/5427/993/63967/5111 | 4.41E-05 | 8.59E-04 | 5.43E-05 | 1.82E-04 |
|  | E2F_Q3_01 | transcription factor targets | 983/990/9833/79733/144455/  4173/5427/993/54962/5111 | 4.57E-05 | 8.87E-04 | 5.62E-05 | 4.18E-05 |
|  | E2F_Q4 |  | 6241/983/990/79733/144455/  4173/5427/993/63967/5111 | 4.74E-05 | 4.74E-05 | 5.62E-05 | 1.96E-04 |
| **C4** | GNF2_RRM2 | Neighborhood of RRM2 ribonucleotide reductase M2 polypeptide in the GNF2 expression compendium | 6241/699/9787/7298/3161/  7153/9133/983/9833/701/890/  3832/9212/332/4173/  146909/9700/51203/11065 | 2.19E-32 | 2.94E-31 | 4.06E-30 | 2.18E-29 |
|  | MORF_BUB1 | Neighborhood of BUB1 BUB1 budding uninhibited by benzimidazoles 1 homolog (yeast) in the MORF expression compendium | 699/9787/3161/11004/701/  3832/9700/9928/10615/891 | 9.60E-19 | 7.84E-19 | 1.32E-18 | 9.07E-21 |
|  | GNF2_CCNB2 | Neighborhood of CCNB2 cyclin B2 in the GNF2 expression compendium | 6241/699/9787/7298/79801/  55872/3161/7153/9133/983/  11004/7272/9833/701/890 | 2.25E-45 | 8.07E-45 | 5.86E-43 | 6.37E-47 |
|  | GNF2_CDC2 | Neighborhood of CDC2 cell division cycle 2, G1 to S and G2 to M in the GNF2 expression compendium | 6241/9787/7298/79801/3161/  64151/7153/9133/983/7272/  9833/79733/890/3832/10112 | 6.44E-38 | 1.59E-37 | 1.03E-35 | 1.32E-03 |
|  | GNF2_CENPF | Neighborhood of CENPF centromere protein F, 350/400ka (mitosin) in the GNF2 expression compendium | 6241/9787/7298/79801/3161/  7153/9133/983/7272/79733/890/  3832/24137/55355/56992 | 2.60E-32 | 3.27E-03 | 3.05E-30 | 2.76E-32 |
|  | GNF2_HMMR | Neighborhood of HMMR hyaluronan-mediated motility receptor (RHAMM) in the GNF2 expression compendium | 6241/699/9787/7298/79801/3161/  7153/9133/983/7272/9833/701/  79733/890/3832/10112/9212/332/4173 | 2.07E-40 | 1.46E-38 | 5.78E-36 | 1.22E-38 |
|  | GNF2_PCNA | Neighborhood of PCNA proliferating cell nuclear antigen in the GNF2 expression compendium | 6241/9787/7298/79801/3161/7153/  9133/983/7272/9833/701/890/  3832/56992/332/9837/4173/10635 | 1.18E-34 | 1.56E-32 | 8.35E-31 | 1.31E-32 |
|  | GNF2_CENPE | Neighborhood of CENPE centromere protein E, 312kDa in the GNF2 expression compendium | 6241/699/9787/7298/79801/3161/  9133/983/890/3832/9212/332/  146909/9700/51203/11065/  83461/1062/4605/5111 | 4.50E-28 | 3.86E-27 | 7.45E-28 | 3.39E-27 |
| **C5** | GO_CHROMOSOME_  SEGREGATION | The process in which genetic material, in the form of chromosomes, is organized into specific structures and then physically separated and apportioned to two or more sets. | 699/9787/64151/57405/7153/  990/11004/7272/701/113130/  24137/55355/11339/9212/332/9319 | 1.43E-27 | 1.33E-25 | 2.33E-21 | 4.40E-22 |
|  | GO_MITOTIC_  NUCLEAR_DIVISION | A mitotic cell cycle process comprising the steps by which the nucleus of a eukaryotic cell divides | 699/9787/64151/9133/990/  11004/7272/1111/701/  113130/890/3832/24137/9212 | 1.08E-26 | 5.22E-25 | 2.33E-21 | 8.50E-25 |
|  | GO_ORGANELLE_  FISSION | The creation of two or more organelles by division of one organelle. | 699/9787/64151/9133/990/  11004/7272/1111/701/  113130/890/3832/24137/  9212/332/9319/146909 | 3.63E-23 | 3.37E-22 | 8.18E-20 | 6.65E-22 |
|  | GO_NUCLEAR_  CHROMOSOME_  SEGREGATION | The process in which genetic material, in the form of nuclear chromosomes, is organized into specific structures and then physically separated and apportioned to two or more sets. | 699/9787/64151/7153/9133/990/  11004/7272/1111/701/113130/  890/3832/24137/9212/332/9319 | 1.78E-21 | 2.13E-20 | 3.91E-19 | 1.20E-20 |
|  | GO_MITOTIC_SISTER_  CHROMATID_  SEGREGATION | The cell cycle process in which replicated homologous chromosomes are organized and then physically separated and apportioned to two sets during the mitotic cell cycle. | 699/9787/64151/990/11004/  7272/701/113130/24137/  9212/9319/146909 | 5.71E-21 | 6.32E-20 | 4.77E-19 | 1.28E-20 |
|  | GO_SISTER_  CHROMATID_  SEGREGATION | The cell cycle process in which sister chromatids are organized and then physically separated and apportioned to two or more sets. | 699/9787/64151/7153/990/  11004/7272/701/113130  /24137/9212/9319/146909/4751 | 4.67E-20 | 3.37E-19 | 5.52E-19 | 3.34E-20 |
|  | GO_CHROMOSOME_  CENTROMERIC_  REGION | The region of a chromosome that includes the centromeric DNA and associated proteins. | 699/64151/57405/11004/7272/  701/113130/55355/11339/  9212/332/4751/83540/57082 | 7.57E-20 | 7.76E-18 | 9.27E-14 | 2.17E-14 |
|  | GO_CONDENSED_  CHROMOSOME | A highly compacted molecule of DNA and associated proteins resulting in a cytologically distinct structure. | 699/64151/57405/7153/11004/  1111/701/113130/55355/  9212/332/4751/23397 | 1.77E-19 | 7.94E-18 | 2.04E-13 | 5.69E-14 |
| **C6** | VEGF_A_UP.V1_DN | Genes down-regulated in HUVEC cells (endothelium) by treatment with VEGFA | 6241/699/9787/3161/9133/  7272/701/3832/332/4173/  10615/1062/891/10733/9156 | 0.00137 | 1.56E-08 | 4.50E-07 | 1.70E-08 |
|  | CSR_LATE_UP.V1_UP | Genes up-regulated in late serum response of CRL 2091 cells (foreskin fibroblasts). | 6241/79801/55872/983/990/  29128/332/9319/11065/  83540/4605/6941/63967/81610/7283/64785 | 0.001377 | 1.95E-10 | 9.20E-09 | 1.70E-09 |
|  | E2F1_UP.V1_UP | Genes up-regulated in late serum response of CRL 2091 cells | 699/9787/7298/7153/1111/  79733/9212/4173/  7083/23397/29089/6491/3149/10733 | 0.001377 | 4.76E-07 | 3.76E-07 | 1.34E-08 |
|  | RPS14_DN.V1_DN | Genes down-regulated in CD34+ hematopoietic progenitor cells after knockdown of RPS14 [GeneID=6208] by RNAi. | 9787/55872/9133/990/11004/  79733/10112/56992/9319/  9700/7083/23397/83461/4998 | 0.001379 | 2.45E-13 | 4.87E-13 | 2.86E-15 |
|  | HOXA9_DN.V1_DN | Genes down-regulated in MOLM-14 cells (AML) with knockdown of HOXA9 [GeneID=3205] gene by RNAi vs controls. | 55165/990/79733/9837/  3070/4998/55215/10733/54962/  29028/64785/55388/10276 | 0.001395 | 1.11E-03 | 8.86E-04 | 1.16E-03 |
|  | RB_P107_DN.V1_UP | Genes up-regulated in primary keratinocytes from RB1 and RBL1 [GeneID=5925] [GeneID=5933] skin specific knockout | 6241/79733/24137/5427/23397/  9134/3959/6941/7283/5111/  898/10549/4175/899 | 0.001403 | 1.45E-03 | 1.89E-03 | 3.12E-06 |
|  | GCNP_SHH_UP  _LATE.V1_UP | Genes up-regulated in granule cell neuron precursors (GCNPs) after stimulation with Shh for 24h. | 6241/79733/24137/5427/23397/  9134/3959/6941/7283/5111/898/  10549/4175/899/5983/5557 | 0.001406 | 1.89E-03 | 3.94E-08 | 6.38E-08 |
|  | MTOR_UP.V1_UP | Genes up-regulated by everolimus [PubChem=6442177] in prostate tissue. | 6241/9787/7153/983/11004/  1111/79733/9212/9319/  4173/51203/7083/993/4175 | 0.001427 | 1.08E-05 | 3.49E-05 | 5.12E-04 |
|  | PRC2_EZH2_UP.V1_DN | Genes down-regulated in TIG3 cells (fibroblasts) upon knockdown of EZH2 [GeneID=2146] gene | 6241/983/29128/11274/11065/  6614/3070/54962/6941/  5111/4175/1718/4939/4522 | 0.001427 | 7.94E-08 | 6.51E-09 | 5.12E-07 |
| **C7** | GSE15750_DAY6_VS_  DAY10_TRAF6KO_  EFF_CD8_TCELL_UP | Genes up-regulated in comparison of wild type CD8 effector T cells at day 6 versus those from mice defficient for TRAF6 [GeneID=7189] at day 10. | 6241/55165/699/79801/55872/  157313/3161/64151/57405/7153/  9133/983/990/7272/29128/1111/9833  /701/79733 | 5.07E-68 | 2.06E-62 | 2.99E-35 | 6.48E-55 |
|  | GSE15750_DAY6_  VS_DAY10_EFF_  CD8_TCELL_UP | Genes up-regulated in comparison of wild type CD8 effector T cells at day 6 versus those at day 10. | 6241/55165/699/79801/55872/3161/  64151/57405/7153/9133/983/990/  11004/7272/29128/9833/701 | 1.16E-62 | 3.10E-50 | 2.99E-35 | 3.10E-50 |
|  | GSE30962_PRIMARY_  VS_SECONDARY_  ACUTE_  LCMV_INF_CD8_  TCELL_UP | Genes up-regulated in comparison of splenic primary CD8 effector T cells at day 8 post-acute infection versus splenic secondary CD8 effector T cells at day 8 post-acute infection. | 6241/55165/699/9787/79801/  57405/7153/9133/990/7272/29128/  9833/701/79733/113130/890/10112/24137/  55355 | 2.35E-52 | 9.38E-50 | 2.99E-35 | 1.11E-45 |
|  | GSE13547_CTRL_VS_  ANTI_IGM_STIM_  BCELL_12H_UP | Genes up-regulated in B lymphocytes: control versus stimulated by anti-IgM for 12h. | 6241/55165/699/9787/79801/55872/  3161/64151/57405/7153/9133/  983/990/7272/29128/1111 | 1.92E-51 | 7.53E-41 | 7.68E-47 | 1.92E-46 |
|  | GSE39110_DAY3_VS_  DAY6_POST_  IMMUNIZATION_  CD8_TCELL_DN | Genes down-regulated in CD8 T cells after immunization: day 3 versus day 6. | 55165/699/79801/55872/57405/  7153/9133/983/990/11004/7272/  29128/1111/9833/79733/113130 | 4.81E-49 | 1.34E-36 | 8.11E-40 | 1.40E-35 |
|  | GSE36476_CTRL_VS_  TSST_ACT_40H_  MEMORY_CD4_  TCELL_OLD_DN | Genes down-regulated in comparison of untreated CD4 [GeneID=920] memory T cells from old donors versus those treated with TSST at 40 h. | 6241/55165/699/9787/7298/  79801/55872/3161/64151/7153/  9133/990/11004/7272/1111/701 | 5.64E-38 | 1.34E-36 | 2.99E-35 | 1.40E-35 |
|  | GSE36476_CTRL_VS_  TSST_ACT_72H_  MEMORY_CD4_  TCELL_YOUNG_DN | Genes down-regulated in comparison of untreated CD4 [GeneID=920] memory T cells from young donors versus those treated with TSST at 72 h. | 6241/55165/699/9787/7298/79801/55872/  3161/64151/7153/9133/990/11004  /7272/1111/701/3832 | 8.05E-45 | 7.74E-47 | 4.85E-43 | 2.27E-34 |
|  | GSE25088_WT_VS_  STAT6_KO_  MACROPHAGE_  IL4_STIM_DN | Genes down-regulated in bone marrow-derived macrophages treated with IL4 [GeneID=3565]: wildtype versus STAT6 | 6241/55165/9787/7298/3161/51237  /64151/7153/9133/983/9833/701/  79733/3832/10112/24137 | 1.12E-39 | 2.17E-35 | 6.44E-37 | 2.27E-34 |
|  | GSE24634_TREG_VS_  TCONV_POST_DAY7_  IL4_CONVERSION_UP | Genes up-regulated in comparison of CD25+ regulatory T cell (Treg) treated with IL4  [GeneID=3565] at day 7 versus CD25- T cells treated with IL4 | 6241/55165/699/9787/7298/  9133/11004/  9833/890/3832/24137/  55355/56992/9212/332 | 1.14E-30 | 1.31E-02 | 2.55E-25 | 2.41E-26 |
|  | GSE45365_WT_VS_  IFNAR_KO_BCELL_  MCMV_INFECTION_DN | Genes down-regulated in CD8 T cells: control versus primary acute viral infection. | 55165/9787/9133/11004/701/113130/  10112/24137/55355/  11339/332/9319/857/146909/4751 | 1.16E-29 | 2.20E-30 | 4.27E-19 | 3.12E-24 |

Table-S3: Molecular signature genes that were identified from annotated gene sets from MSigDb.

| **Summary statistics** | **GSE51808** |
| --- | --- |
| Number of nodes | 34 |
| Number of edges | 422 |
| Average number of neighbours | 24.827 |
| Network diameter | 2 |
| Network radius | 1 |
| Characteristic path length | 1.084 |
| Clustering coefficient | 0.447 |
| Network density | 0.376 |
| Connected components | 2 |
| Multi edge node pairs | 0 |
| Number of self loops | 0 |

Tabke-S4: Cytoscape network analysis from Combined proteins from 4 groups and its interaction summary statistics.

| **DF-CO** | **SD-CO** | **CP-DF** | **CP-SD** |
| --- | --- | --- | --- |
| AURKB | BUB1 | BUB1B | BUB1B |
| DLGAP5 | CCNB2 | CCNB2P5 | CCNB2 |
| NCAPG | MELK | MELK | MELK |
| CCNB2 | BIRC5 | BIRC5 | BIRC5 |
| KIF11 | BUB1 | BUB1B | BUB1B |
| BUB1B | PBK | PBK | PBK |
| CCNB2 | AURKB | AURKB | AURKB |
| MELK | NUSAP1 | RRM2 | RRM2 |
| BIRC5 | TOP2A | NUSAP1 | NUSAP1 |
| BUB1 | TTK | TOP2A | TOP2A |

Table-S5: CytoHubba analysis for the identification of 10 Hub proteins which were involved in essential for regulatory networks.

| **ID** | **Term** | **% Associated Genes** | **Number of Genes** | **Regu lation** | ***Associated***  ***Genes Found*** |
| --- | --- | --- | --- | --- | --- |
| **DF-CO** |  |  |  |  |  |
| GO:0002478 | antigen processing and presentation of exogenous peptide antigen | 18.27956963 | 34 | up | *[AP1B1, AP1S1, CALR, CENPE, DCTN6, DNM2, KIF11, KIF15, KIF2C, KIF4A, KIFAP3, KLC1, LAG3, LGMN, PDIA3, PSMA2, PSMA3, PSMA4, PSMA5, PSMB5, PSMB6, PSMB7, PSMB9, PSMC4, PSMD14, PSMD2, PSMD3, PSMD6, PSMD7, PSMD8, SAR1B, SEC24A, SEC24D, SEM1]* |
| GO:0019886 | antigen processing and presentation of exogenous peptide antigen via MHC class II | 15.53398037 | 16 | up | *[AP1B1, AP1S1, CENPE, DCTN6, DNM2, KIF11, KIF15, KIF2C, KIF4A, KIFAP3, KLC1, LAG3, LGMN, SAR1B, SEC24A, SEC24D]* |
| GO:0002474 | antigen processing and presentation of peptide antigen via MHC class I | 22.3300972 | 23 | up | *[CALR, IDE, MR1, PDIA3, PSMA2, PSMA3, PSMA4, PSMA5, PSMB5, PSMB6, PSMB7, PSMB9, PSMC4, PSMD14, PSMD2, PSMD3, PSMD6, PSMD7, PSMD8, SAR1B, SEC24A, SEC24D, SEM1]* |
| GO:0002218 | activation of innate immune response | 13.2911396 | 21 | up | *[HRAS, HSP90AA1, NRAS, PSMA2, PSMA3, PSMA4, PSMA5, PSMB5, PSMB6, PSMB7, PSMB9, PSMC4, PSMD14, PSMD2, PSMD3, PSMD6, PSMD7, PSMD8, PSPC1, SEM1, TOMM70]* |
| GO:0002479 | antigen processing and presentation of exogenous peptide antigen via MHC class I, TAP-dependent | 22.5 | 18 | up | *[CALR, PDIA3, PSMA2, PSMA3, PSMA4, PSMA5, PSMB5, PSMB6, PSMB7, PSMB9, PSMC4, PSMD14, PSMD2, PSMD3, PSMD6, PSMD7, PSMD8, SEM1]* |
| GO:0060218 | hematopoietic stem cell differentiation | 18.0851059 | 17 | up | *[BATF, PSMA2, PSMA3, PSMA4, PSMA5, PSMB5, PSMB6, PSMB7, PSMB9, PSMC4, PSMD14, PSMD2, PSMD3, PSMD6, PSMD7, PSMD8, SEM1]* |
| GO:1901532 | regulation of hematopoietic progenitor cell differentiation | 17.70833397 | 17 | up | *[NUDT21, PSMA2, PSMA3, PSMA4, PSMA5, PSMB5, PSMB6, PSMB7, PSMB9, PSMC4, PSMD14, PSMD2, PSMD3, PSMD6, PSMD7, PSMD8, SEM1]* |
| GO:0002223 | stimulatory C-type lectin receptor signalling pathway | 14.87603283 | 18 | up | *[HRAS, NRAS, PSMA2, PSMA3, PSMA4, PSMA5, PSMB5, PSMB6, PSMB7, PSMB9, PSMC4, PSMD14, PSMD2, PSMD3, PSMD6, PSMD7, PSMD8, SEM1]* |
| GO:0030097 | hemopoiesis | 9.194214821 | 89 | down | *[ACTN1, ACVR2A, AGO4, ANGPT1, ATM, BRAF, CCR6, CD40LG, CDK13, CHD2, CR1, CREB1, CREBBP, CUL4A, ERCC1, F2RL1, FAM210B, FAXDC2, FBXO7, FLVCR1, FNIP1, FOXP1, GAB3, GATA1, GATA2, HCLS1, HIPK1, IL4, IL6R, IL7R, ITGA2B, ITGB8, ITPKB, JAM3, KAT6A, KMT2C, KMT2D, LEPROT, MBD4, MKNK2, MPL, MS4A1, MTURN, N4BP2L2, NCKAP1L, NDFIP1, NOTCH1, PBX1, PICALM, PIK3CD, PIP4K2A, PREX1, PRKCA, PSEN1, PSMB2, PSME4, PTBP3, PTGER4, PTPRC, PTPRJ, RASSF2, RC3H1, RCOR1, RPL22, SIN3A, SLC25A38, SLC46A2, SLC4A1, SNRK, STAT5B, STK11, STK4, TESC, TET2, TGFBR2, THRA, TMEM91, TNRC6B, TNRC6C, TRAF6, TSC1, TSPAN2, WASF2, XRCC5, YY1, ZC3H8, ZFP36L1, ZFP36L2, ZMIZ1]* |
| GO:0030099 | myeloid cell differentiation | 11.01123619 | 49 | down | *[ACTN1, ACVR2A, AGO4, CREB1, CREBBP, CUL4A, F2RL1, FAM210B, FAXDC2, FLVCR1, FOXP1, GAB3, GATA1, GATA2, HCLS1, IL4, ITGA2B, ITGB8, ITPKB, KAT6A, KMT2C, KMT2D, MBD4, MPL, MTURN, NCKAP1L, NDFIP1, PIK3CD, PIP4K2A, PRKCA, PSEN1, PTBP3, RASSF2, RCOR1, SIN3A, SLC25A38, SLC4A1, SNRK, STAT5B, TESC, TET2, TGFBR2, THRA, TNRC6B, TNRC6C, TRAF6, TSPAN2, WASF2, ZFP36L1]* |
| GO:0045639 | positive regulation of myeloid cell differentiation | 16.34615326 | 17 | down | *[ACVR2A, CREB1, FAM210B, FAXDC2, GATA1, GATA2, HCLS1, MBD4, MPL, MTURN, NCKAP1L, PRKCA, RCOR1, STAT5B, TESC, TRAF6, ZFP36L1]* |
| **SD-CO** | | | | | |
| GO:0002478 | antigen processing and presentation of exogenous peptide antigen | 17.74193573 | 33 | up | *[AP1B1, CALR, CENPE, CTSV, DCTN6, DNM2, KIF11, KIF15, KIF2C, KIF4A, KIFAP3, KLC1, LAG3, LGMN, PDIA3, PSMA2, PSMA3, PSMA4, PSMA5, PSMA8, PSMB5, PSMB6, PSMB7, PSMC4, PSMD14, PSMD2, PSMD3, PSMD6, PSMD8, SAR1B, SEC24A, SEC24D, SEM1]* |
| GO:0019886 | antigen processing and presentation of exogenous peptide antigen via MHC class II | 15.53398037 | 16 | up | *[AP1B1, CENPE, CTSV, DCTN6, DNM2, KIF11, KIF15, KIF2C, KIF4A, KIFAP3, KLC1, LAG3, LGMN, SAR1B, SEC24A, SEC24D]* |
| GO:0002474 | antigen processing and presentation of peptide antigen via MHC class I | 22.3300972 | 23 | up | *[CALR, IDE, MR1, PDIA3, PNKD, PSMA2, PSMA3, PSMA4, PSMA5, PSMA8, PSMB5, PSMB6, PSMB7, PSMC4, PSMD14, PSMD2, PSMD3, PSMD6, PSMD8, SAR1B, SEC24A, SEC24D, SEM1]* |
| GO:0002479 | antigen processing and presentation of exogenous peptide antigen via MHC class I, TAP-dependent | 21.25 | 17 | up | *[CALR, PDIA3, PSMA2, PSMA3, PSMA4, PSMA5, PSMA8, PSMB5, PSMB6, PSMB7, PSMC4, PSMD14, PSMD2, PSMD3, PSMD6, PSMD8, SEM1]* |
| GO:0060218 | hematopoietic stem cell differentiation | 17.02127647 | 16 | up | *[PSMA2, PSMA3, PSMA4, PSMA5, PSMA8, PSMB5, PSMB6, PSMB7, PSMC4, PSMD14, PSMD2, PSMD3, PSMD6, PSMD8, SEM1, YTHDF2]* |
| GO:1901532 | regulation of hematopoietic progenitor cell differentiation | 18.75 | 18 | up | *[NUDT21, PSMA2, PSMA3, PSMA4, PSMA5, PSMA8, PSMB5, PSMB6, PSMB7, PSMC4, PSMD14, PSMD2, PSMD3, PSMD6, PSMD8, SEM1, SOS1, YTHDF2]* |
| GO:1902036 | regulation of hematopoietic stem cell differentiation | 20.5128212 | 16 | up | *[PSMA2, PSMA3, PSMA4, PSMA5, PSMA8, PSMB5, PSMB6, PSMB7, PSMC4, PSMD14, PSMD2, PSMD3, PSMD6, PSMD8, SEM1, YTHDF2]* |
| GO:0030097 | hemopoiesis | 10.3305788 | 100 | down | *[ACTN1, ACVR2A, AGO4, ANGPT1, ANXA1, ATM, BRAF, CCR6, CD40LG, CDK13, CR1, CREB1, CREBBP, CRIP2, CRTAM, CTNNBIP1, CUL4A, ERCC1, F2RL1, FAM210B, FBXO7, FLVCR1, FNIP1, FOXP1, GAB3, GATA1, GATA2, HCLS1, HHEX, HIPK1, IKZF1, IL1B, IL4R, IL6R, IL7R, IRF2BP2, ITGB8, ITPKB, KAT6A, KLF1, KMT2C, KMT2D, LEPROT, LFNG, LYN, MKNK2, MS4A1, MS4A2, MTURN, N4BP2L2, NCKAP1L, NDFIP1, NFAM1, NOTCH1, PBX1, PICALM, PIK3CD, PIM1, PIP4K2A, PREX1, PRKCA, PSEN1, PSMB2, PSME4, PTBP3, PTGER4, PTPRC, PTPRJ, RASSF2, RC3H1, RC3H2, RCOR1, RPL22, SIN3A, SLC25A38, SLC46A2, SLC4A1, SNRK, SNX10, SOS2, SOX13, STAT5B, STK11, STK4, TESC, TET2, TGFBR2, TMEM91, TNRC6B, TNRC6C, TSC1, TSPAN2, VSIR, WASF2, XRCC5, YY1, ZC3H8, ZFP36L1, ZFP36L2, ZMIZ1]* |
| GO:0002366 | leukocyte activation involved in immune response | 11.50442505 | 91 | down | *[ACP3, ADGRE3, ANXA1, ARHGAP45, ATP6V0C, C5AR1, CANT1, CAT, CBL, CCR6, CD1C, CD244, CD40LG, CD84, CDK13, CHI3L1, CMTM6, CPPED1, CR1, CTSB, CTSC, CTSS, CXCL1, CXCR2, DDX3X, ERCC1, F2RL1, FCGR2A, FOXP1, GATA2, GCA, GLIPR1, GMFG, GOLGA7, HLA-F, HVCN1, IL4R, IL6R, IMPDH1, IST1, ITGAX, KMT5B, KRT1, LAMP2, LAT, LAT2, LFNG, LILRA2, LRMP, LYN, MANBA, MAPK1, MGAM, MMP28, MOSPD2, MS4A3, NCKAP1L, NDFIP1, NFAM1, PADI2, PECAM1, PIK3CD, PIKFYVE, PSEN1, PTAFR, PTGDR, PTGER4, PTPRC, PTPRJ, PYGL, RAB18, RAB37, RAB3D, RAB5B, RC3H1, RC3H2, RHOG, RIF1, ROCK1, SH2D1B, SIRPA, SLC15A4, SLC44A2, SLPI, STK10, STXBP3, TBC1D10C, TSC1, TUBB2A, VAPA, XRCC5]* |
| GO:0002444 | myeloid leukocyte mediated immunity | 11.7363348 | 73 | down | *[ACP3, ADGRE3, ARHGAP45, ATP6V0C, C5AR1, CANT1, CAT, CBL, CD84, CDK13, CHI3L1, CMTM6, CPPED1, CR1, CTSB, CTSC, CTSS, CXCL1, CXCL6, CXCR2, DDX3X, F2RL1, FCGR2A, GATA2, GCA, GLIPR1, GMFG, GOLGA7, HVCN1, IL4R, IMPDH1, IST1, ITGAX, KRT1, LAMP2, LAT, LAT2, LRMP, LYN, MANBA, MAPK1, MGAM, MMP28, MOSPD2, MS4A3, NCKAP1L, NFAM1, PADI2, PECAM1, PIK3CD, PIKFYVE, PSEN1, PTAFR, PTGDR, PTPRC, PTPRJ, PYGL, RAB18, RAB37, RAB3D, RAB5B, RHOG, ROCK1, SIRPA, SLC15A4, SLC44A2, SLPI, STK10, STXBP3, TBC1D10C, TUBB2A, VAPA, XRCC5]* |
| GO:0002275 | myeloid cell activation involved in immune response | 11.88925076 | 73 | down | *[ACP3, ADGRE3, ARHGAP45, ATP6V0C, C5AR1, CANT1, CAT, CBL, CD84, CDK13, CHI3L1, CMTM6, CPPED1, CR1, CTSB, CTSC, CTSS, CXCL1, CXCR2, DDX3X, F2RL1, FCGR2A, GATA2, GCA, GLIPR1, GMFG, GOLGA7, HVCN1, IL4R, IMPDH1, IST1, ITGAX, KRT1, LAMP2, LAT, LAT2, LILRA2, LRMP, LYN, MANBA, MAPK1, MGAM, MMP28, MOSPD2, MS4A3, NCKAP1L, NFAM1, PADI2, PECAM1, PIK3CD, PIKFYVE, PSEN1, PTAFR, PTGDR, PTPRC, PTPRJ, PYGL, RAB18, RAB37, RAB3D, RAB5B, RHOG, ROCK1, SIRPA, SLC15A4, SLC44A2, SLPI, STK10, STXBP3, TBC1D10C, TUBB2A, VAPA, XRCC5]* |
| GO:0002446 | neutrophil mediated immunity | 11.51079178 | 64 | down | *[ACP3, ADGRE3, ARHGAP45, ATP6V0C, C5AR1, CANT1, CAT, CDK13, CHI3L1, CMTM6, CPPED1, CR1, CTSB, CTSC, CTSS, CXCL1, CXCL6, CXCR2, DDX3X, F2RL1, FCGR2A, GCA, GLIPR1, GMFG, GOLGA7, HVCN1, IMPDH1, IST1, ITGAX, KRT1, LAMP2, LRMP, MANBA, MAPK1, MGAM, MMP28, MOSPD2, MS4A3, NCKAP1L, NFAM1, PADI2, PECAM1, PIKFYVE, PSEN1, PTAFR, PTPRC, PTPRJ, PYGL, RAB18, RAB37, RAB3D, RAB5B, RHOG, ROCK1, SIRPA, SLC15A4, SLC44A2, SLPI, STK10, STXBP3, TBC1D10C, TUBB2A, VAPA, XRCC5]* |
| GO:0042119 | neutrophil activation | 11.89189148 | 66 | down | *[ACP3, ADGRE3, ARHGAP45, ATP6V0C, C5AR1, CANT1, CAT, CDK13, CHI3L1, CMTM6, CPPED1, CR1, CTSB, CTSC, CTSS, CXCL1, CXCL6, CXCR2, DDX3X, F2RL1, FCGR2A, GCA, GLIPR1, GMFG, GOLGA7, HVCN1, IMPDH1, IST1, ITGAX, KRT1, LAMP2, LILRA2, LRMP, MANBA, MAPK1, MGAM, MMP28, MOSPD2, MS4A3, NCKAP1L, NFAM1, PADI2, PECAM1, PIKFYVE, PREX1, PSEN1, PTAFR, PTPRC, PTPRJ, PYGL, RAB18, RAB37, RAB3D, RAB5B, RHOG, ROCK1, SIRPA, SLC15A4, SLC44A2, SLPI, STK10, STXBP3, TBC1D10C, TUBB2A, VAPA, XRCC5]* |
| GO:0002283 | neutrophil activation involved in immune response | 11.60221004 | 63 | down | *[ACP3, ADGRE3, ARHGAP45, ATP6V0C, C5AR1, CANT1, CAT, CDK13, CHI3L1, CMTM6, CPPED1, CR1, CTSB, CTSC, CTSS, CXCL1, CXCR2, DDX3X, FCGR2A, GCA, GLIPR1, GMFG, GOLGA7, HVCN1, IMPDH1, IST1, ITGAX, KRT1, LAMP2, LILRA2, LRMP, MANBA, MAPK1, MGAM, MMP28, MOSPD2, MS4A3, NCKAP1L, NFAM1, PADI2, PECAM1, PIKFYVE, PSEN1, PTAFR, PTPRC, PTPRJ, PYGL, RAB18, RAB37, RAB3D, RAB5B, RHOG, ROCK1, SIRPA, SLC15A4, SLC44A2, SLPI, STK10, STXBP3, TBC1D10C, TUBB2A, VAPA, XRCC5]* |
| **CP-DF** | | | | | |
| GO:0051607 | defense response to virus | 18.01470566 | 49 | down | *[AIM2, ANKRD17, APOBEC3F, APOBEC3G, BST2, CXCL10, DDX1, DDX21, ELMOD2, G3BP1, GBP1, GBP3, GPAM, HSP90AA1, IFI27, IFI44L, IFI6, IFIH1, IFNG, IFNLR1, IL12RB1, ILF3, ILRUN, ISG20, MICB, MX1, MYDGF, NDUFAF4, OAS1, OAS2, PDE12, POLR3A, POLR3C, POLR3G, PRF1, RNASE1, RNASE2, RTP4, SLFN13, STAT1, STAT2, STING1, TLR7, TOMM70, TTC4, VAMP8, ZC3HAV1, ZMPSTE24, ZMYND11]* |
| GO:0002474 | antigen processing and presentation of peptide antigen via MHC class I | 31.06796074 | 32 | down | *[CALR, IDE, MR1, PNKD, PSMA2, PSMA3, PSMA4, PSMA5, PSMA8, PSMB10, PSMB3, PSMB5, PSMB6, PSMB7, PSMB9, PSMC2, PSMC4, PSMC6, PSMD14, PSMD2, PSMD3, PSMD5, PSMD6, PSMD7, PSMD8, PSME1, SAR1B, SEC23A, SEC24A, SEC24D, SEM1, VAMP8]* |
| GO:0002478 | antigen processing and presentation of exogenous peptide antigen | 24.1935482 | 45 | down | *[AP1S1, CALR, CENPE, CLTC, DCTN5, DNM2, DYNLL1, KIF11, KIF15, KIF2C, KIF3B, KIF4A, KIFAP3, KLC1, LAG3, LGMN, PRICKLE1, PSMA2, PSMA3, PSMA4, PSMA5, PSMA8, PSMB10, PSMB3, PSMB5, PSMB6, PSMB7, PSMB9, PSMC2, PSMC4, PSMC6, PSMD14, PSMD2, PSMD3, PSMD5, PSMD6, PSMD7, PSMD8, PSME1, SAR1B, SEC23A, SEC24A, SEC24D, SEM1, VAMP8]* |
| GO:0045088 | regulation of innate immune response | 15.86826324 | 53 | down | *[AIM2, EHHADH, HAVCR2, HRAS, HSP90AA1, ICAM2, IFNG, JAK2, LAG3, LRP8, MED1, NR1H3, NRAS, OAS1, POLR3C, POLR3G, PRKCA, PSMA2, PSMA3, PSMA4, PSMA5, PSMA8, PSMB10, PSMB3, PSMB5, PSMB6, PSMB7, PSMB9, PSMC2, PSMC4, PSMC6, PSMD14, PSMD2, PSMD3, PSMD5, PSMD6, PSMD7, PSMD8, PSME1, PSPC1, PTPN1, PTPN11, PTPN2, SEM1, SERPING1, STAT1, STAT2, STING1, TOMM70, UBE2K, USP18, XRCC6, YTHDF2]* |
| GO:0002244 | hematopoietic progenitor cell differentiation | 20.652174 | 38 | down | *[AGPAT5, BATF, BVES, ESCO2, METTL14, MYB, NUDT21, PSMA2, PSMA3, PSMA4, PSMA5, PSMA8, PSMB10, PSMB3, PSMB5, PSMB6, PSMB7, PSMB9, PSMC2, PSMC4, PSMC6, PSMD14, PSMD2, PSMD3, PSMD5, PSMD6, PSMD7, PSMD8, PSME1, PUS7, SEM1, SLC7A6OS, SOS1, TCF12, TNFRSF13B, TOP2A, TP53, YTHDF2]* |
| GO:0045089 | positive regulation of innate immune response | 16.87763786 | 40 | down | *[AIM2, EHHADH, HAVCR2, HRAS, HSP90AA1, ICAM2, LAG3, MED1, NRAS, POLR3C, POLR3G, PRKCA, PSMA2, PSMA3, PSMA4, PSMA5, PSMA8, PSMB10, PSMB3, PSMB5, PSMB6, PSMB7, PSMB9, PSMC2, PSMC4, PSMC6, PSMD14, PSMD2, PSMD3, PSMD5, PSMD6, PSMD7, PSMD8, PSME1, PSPC1, SEM1, STING1, TOMM70, UBE2K, XRCC6]* |
| GO:0002218 | activation of innate immune response | 20.88607597 | 33 | down | *[AIM2, HRAS, HSP90AA1, ICAM2, NRAS, PRKCA, PSMA2, PSMA3, PSMA4, PSMA5, PSMA8, PSMB10, PSMB3, PSMB5, PSMB6, PSMB7, PSMB9, PSMC2, PSMC4, PSMC6, PSMD14, PSMD2, PSMD3, PSMD5, PSMD6, PSMD7, PSMD8, PSME1, PSPC1, SEM1, STING1, TOMM70, XRCC6]* |
| GO:0002479 | antigen processing and presentation of exogenous peptide antigen via MHC class I, TAP-dependent | 31.25 | 25 | down | *[CALR, PSMA2, PSMA3, PSMA4, PSMA5, PSMA8, PSMB10, PSMB3, PSMB5, PSMB6, PSMB7, PSMB9, PSMC2, PSMC4, PSMC6, PSMD14, PSMD2, PSMD3, PSMD5, PSMD6, PSMD7, PSMD8, PSME1, SEM1, VAMP8]* |
| GO:0060218 | hematopoietic stem cell differentiation | 30.85106468 | 29 | down | *[BATF, MYB, PSMA2, PSMA3, PSMA4, PSMA5, PSMA8, PSMB10, PSMB3, PSMB5, PSMB6, PSMB7, PSMB9, PSMC2, PSMC4, PSMC6, PSMD14, PSMD2, PSMD3, PSMD5, PSMD6, PSMD7, PSMD8, PSME1, PUS7, SEM1, TCF12, TP53, YTHDF2]* |
| GO:1901532 | regulation of hematopoietic progenitor cell differentiation | 31.25 | 30 | down | *[METTL14, MYB, NUDT21, PSMA2, PSMA3, PSMA4, PSMA5, PSMA8, PSMB10, PSMB3, PSMB5, PSMB6, PSMB7, PSMB9, PSMC2, PSMC4, PSMC6, PSMD14, PSMD2, PSMD3, PSMD5, PSMD6, PSMD7, PSMD8, PSME1, PUS7, SEM1, SOS1, TCF12, YTHDF2]* |
| GO:1902036 | regulation of hematopoietic stem cell differentiation | 34.61538315 | 27 | down | *[MYB, PSMA2, PSMA3, PSMA4, PSMA5, PSMA8, PSMB10, PSMB3, PSMB5, PSMB6, PSMB7, PSMB9, PSMC2, PSMC4, PSMC6, PSMD14, PSMD2, PSMD3, PSMD5, PSMD6, PSMD7, PSMD8, PSME1, PUS7, SEM1, TCF12, YTHDF2]* |
| GO:0050852 | T cell receptor signalling pathway | 16.89497757 | 37 | down | *[ADA, BTN3A3, CTLA4, DENND1B, EIF2B1, EIF2B2, EIF2B3, EIF2B5, GBP1, HRAS, LILRB4, PSMA2, PSMA3, PSMA4, PSMA5, PSMA8, PSMB10, PSMB3, PSMB5, PSMB6, PSMB7, PSMB9, PSMC2, PSMC4, PSMC6, PSMD14, PSMD2, PSMD3, PSMD5, PSMD6, PSMD7, PSMD8, PSME1, PTPN2, PVRIG, RAB29, SEM1]* |
| GO:0002223 | stimulatory C-type lectin receptor signalling pathway | 22.31404877 | 27 | down | *[HRAS, ICAM2, NRAS, PRKCA, PSMA2, PSMA3, PSMA4, PSMA5, PSMA8, PSMB10, PSMB3, PSMB5, PSMB6, PSMB7, PSMB9, PSMC2, PSMC4, PSMC6, PSMD14, PSMD2, PSMD3, PSMD5, PSMD6, PSMD7, PSMD8, PSME1, SEM1]* |
| GO:0030097 | hemopoiesis | 11.47 | 111.00 | up | *[ACTN1, ACVR2A, AGO3, AGO4, AHSP, ANGPT1, ARHGEF7, BRAF, C17orf99, CA2, CDK13, CHD2, CR1, CREB1, CREBBP, CTNNB1, CTNNBIP1, CUL4A, ERCC1, F2RL1, FAM210B, FAXDC2, FBXO7, FLCN, FLVCR1, FNIP1, FOXP1, GAB3, GATA1, GATA2, GNAO1, HIPK1, IL4R, ITGA2B, ITGB8, ITPKB, JAM3, KAT6A, KAT8, KLF1, KMT2A, KMT2C, KMT2D, L3MBTL3, LEPROT, MBD4, MKNK2, MPL, MS4A1, MTURN, N4BP2L2, NCKAP1L, NDFIP1, NOTCH1, PBX1, PDGFRA, PICALM, PIK3CD, PIK3R1, PIM1, PIP4K2A, PLCG2, PREX1, PRKCA, PRKCB, PSEN1, PSMB1, PSMF1, PTBP3, PTGER4, PTPRC, PTPRJ, RASSF2, RBPJ, RC3H1, RCOR1, ROGDI, RPL22, SH2B3, SIN3A, SLC25A38, SLC4A1, SMAD7, SMPD3, SNRK, SOS2, SP3, ST3GAL1, STAT5B, STK11, STK4, TAZ, TESC, TET2, TGFBR2, THBS1, TMEM64, TMEM91, TNRC6B, TRAF6, TRIM10, TSC1, TSPAN2, VSIR, WASF2, YY1, ZBTB1, ZBTB7A, ZFP36L1, ZFP36L2, ZMIZ1]* |
| GO:0030099 | myeloid cell differentiation | 14.16 | 63.00 | up | *[ACTN1, ACVR2A, AGO3, AGO4, AHSP, CA2, CREB1, CREBBP, CTNNB1, CTNNBIP1, CUL4A, F2RL1, FAM210B, FAXDC2, FLVCR1, FOXP1, GAB3, GATA1, GATA2, ITGA2B, ITGB8, ITPKB, KAT6A, KAT8, KLF1, KMT2A, KMT2C, KMT2D, L3MBTL3, MBD4, MPL, MTURN, NCKAP1L, NDFIP1, PIK3CD, PIK3R1, PIP4K2A, PRKCA, PRKCB, PSEN1, PTBP3, RASSF2, RBPJ, RCOR1, SH2B3, SIN3A, SLC25A38, SLC4A1, SNRK, SP3, STAT5B, TESC, TET2, TGFBR2, THBS1, TMEM64, TNRC6B, TRAF6, TRIM10, TSPAN2, WASF2, ZBTB7A, ZFP36L1]* |
| **CP-SD** | | | | | |
| GO:0002474 | antigen processing and presentation of peptide antigen via MHC class I | 34.95145798 | 36 | down | *[CALR, CANX, IDE, MR1, PDIA3, PNKD, PSMA2, PSMA3, PSMA4, PSMA5, PSMA8, PSMB3, PSMB5, PSMB6, PSMB7, PSMB9, PSMC2, PSMC4, PSMC6, PSMD13, PSMD14, PSMD2, PSMD3, PSMD5, PSMD6, PSMD7, PSMD8, PSME1, SAR1B, SEC23A, SEC24A, SEC24C, SEC24D, SEM1, TAPBP, TAPBPL]* |
| GO:0002478 | antigen processing and presentation of exogenous peptide antigen | 25.2688179 | 47 | down | *[CALR, CANX, CENPE, CLTC, DCTN5, DNM2, DYNLL1, KIF11, KIF15, KIF2C, KIF3B, KIF4A, KIFAP3, KLC1, LAG3, LGMN, PDIA3, PRICKLE1, PSMA2, PSMA3, PSMA4, PSMA5, PSMA8, PSMB3, PSMB5, PSMB6, PSMB7, PSMB9, PSMC2, PSMC4, PSMC6, PSMD13, PSMD14, PSMD2, PSMD3, PSMD5, PSMD6, PSMD7, PSMD8, PSME1, SAR1B, SEC23A, SEC24A, SEC24C, SEC24D, SEM1, TAPBP]* |
| GO:0019886 | antigen processing and presentation of exogenous peptide antigen via MHC class II | 20.38834953 | 21 | down | *[CANX, CENPE, CLTC, DCTN5, DNM2, DYNLL1, KIF11, KIF15, KIF2C, KIF3B, KIF4A, KIFAP3, KLC1, LAG3, LGMN, PRICKLE1, SAR1B, SEC23A, SEC24A, SEC24C, SEC24D]* |
| GO:0002479 | antigen processing and presentation of exogenous peptide antigen via MHC class I, TAP-dependent | 32.5 | 26 | down | *[CALR, PDIA3, PSMA2, PSMA3, PSMA4, PSMA5, PSMA8, PSMB3, PSMB5, PSMB6, PSMB7, PSMB9, PSMC2, PSMC4, PSMC6, PSMD13, PSMD14, PSMD2, PSMD3, PSMD5, PSMD6, PSMD7, PSMD8, PSME1, SEM1, TAPBP]* |
| GO:1902036 | regulation of hematopoietic stem cell differentiation | 34.61538315 | 27 | down | *[PSMA2, PSMA3, PSMA4, PSMA5, PSMA8, PSMB3, PSMB5, PSMB6, PSMB7, PSMB9, PSMC2, PSMC4, PSMC6, PSMD13, PSMD14, PSMD2, PSMD3, PSMD5, PSMD6, PSMD7, PSMD8, PSME1, PUS7, SEM1, TCF12, TP73, YTHDF2]* |
| GO:0002223 | stimulatory C-type lectin receptor signalling pathway | 22.31404877 | 27 | down | *[HRAS, ICAM2, NRAS, PLCG2, PSMA2, PSMA3, PSMA4, PSMA5, PSMA8, PSMB3, PSMB5, PSMB6, PSMB7, PSMB9, PSMC2, PSMC4, PSMC6, PSMD13, PSMD14, PSMD2, PSMD3, PSMD5, PSMD6, PSMD7, PSMD8, PSME1, SEM1]* |
| GO:0002474 | antigen processing and presentation of peptide antigen via MHC class I | 34.95145798 | 36 | down | *[CALR, CANX, IDE, MR1, PDIA3, PNKD, PSMA2, PSMA3, PSMA4, PSMA5, PSMA8, PSMB3, PSMB5, PSMB6, PSMB7, PSMB9, PSMC2, PSMC4, PSMC6, PSMD13, PSMD14, PSMD2, PSMD3, PSMD5, PSMD6, PSMD7, PSMD8, PSME1, SAR1B, SEC23A, SEC24A, SEC24C, SEC24D, SEM1, TAPBP, TAPBPL]* |
| GO:0002478 | antigen processing and presentation of exogenous peptide antigen | 25.2688179 | 47 | down | *[CALR, CANX, CENPE, CLTC, DCTN5, DNM2, DYNLL1, KIF11, KIF15, KIF2C, KIF3B, KIF4A, KIFAP3, KLC1, LAG3, LGMN, PDIA3, PRICKLE1, PSMA2, PSMA3, PSMA4, PSMA5, PSMA8, PSMB3, PSMB5, PSMB6, PSMB7, PSMB9, PSMC2, PSMC4, PSMC6, PSMD13, PSMD14, PSMD2, PSMD3, PSMD5, PSMD6, PSMD7, PSMD8, PSME1, SAR1B, SEC23A, SEC24A, SEC24C, SEC24D, SEM1, TAPBP]* |
| GO:0045088 | regulation of innate immune response | 14.97006035 | 50 | down | *[A2M, AIM2, CADM1, EHHADH, HAVCR2, HRAS, HSP90AA1, ICAM2, IL12A, LAG3, LRP8, MATR3, NR1H3, NRAS, PLCG2, POLR3B, POLR3G, PSMA2, PSMA3, PSMA4, PSMA5, PSMA8, PSMB3, PSMB5, PSMB6, PSMB7, PSMB9, PSMC2, PSMC4, PSMC6, PSMD13, PSMD14, PSMD2, PSMD3, PSMD5, PSMD6, PSMD7, PSMD8, PSME1, PSPC1, PTPN1, PTPN11, PTPN2, SEM1, TNFAIP3, TOMM70, UBE2K, USP18, XRCC6, YTHDF2]* |
| GO:0002244 | hematopoietic progenitor cell differentiation | 21.73913002 | 40 | down | *[AGPAT5, BATF, BVES, CITED2, ESCO2, MIXL1, NUDT21, PSMA2, PSMA3, PSMA4, PSMA5, PSMA8, PSMB3, PSMB5, PSMB6, PSMB7, PSMB9, PSMC2, PSMC4, PSMC6, PSMD13, PSMD14, PSMD2, PSMD3, PSMD5, PSMD6, PSMD7, PSMD8, PSME1, PUS7, SEM1, SLC7A6OS, SOS1, TCF12, TNFRSF13B, TOP2A, TP53, TP73, UFL1, YTHDF2]* |
| GO:0045089 | positive regulation of innate immune response | 17.29957771 | 41 | down | *[AIM2, CADM1, EHHADH, HAVCR2, HRAS, HSP90AA1, ICAM2, IL12A, LAG3, MATR3, NRAS, PLCG2, POLR3B, POLR3G, PSMA2, PSMA3, PSMA4, PSMA5, PSMA8, PSMB3, PSMB5, PSMB6, PSMB7, PSMB9, PSMC2, PSMC4, PSMC6, PSMD13, PSMD14, PSMD2, PSMD3, PSMD5, PSMD6, PSMD7, PSMD8, PSME1, PSPC1, SEM1, TOMM70, UBE2K, XRCC6]* |
| GO:0002218 | activation of innate immune response | 20.88607597 | 33 | down | *[AIM2, HRAS, HSP90AA1, ICAM2, MATR3, NRAS, PLCG2, PSMA2, PSMA3, PSMA4, PSMA5, PSMA8, PSMB3, PSMB5, PSMB6, PSMB7, PSMB9, PSMC2, PSMC4, PSMC6, PSMD13, PSMD14, PSMD2, PSMD3, PSMD5, PSMD6, PSMD7, PSMD8, PSME1, PSPC1, SEM1, TOMM70, XRCC6]* |
| GO:0002479 | antigen processing and presentation of exogenous peptide antigen via MHC class I, TAP-dependent | 32.5 | 26 | down | *[CALR, PDIA3, PSMA2, PSMA3, PSMA4, PSMA5, PSMA8, PSMB3, PSMB5, PSMB6, PSMB7, PSMB9, PSMC2, PSMC4, PSMC6, PSMD13, PSMD14, PSMD2, PSMD3, PSMD5, PSMD6, PSMD7, PSMD8, PSME1, SEM1, TAPBP]* |
| GO:0060218 | hematopoietic stem cell differentiation | 31.9148941 | 30 | down | *[BATF, PSMA2, PSMA3, PSMA4, PSMA5, PSMA8, PSMB3, PSMB5, PSMB6, PSMB7, PSMB9, PSMC2, PSMC4, PSMC6, PSMD13, PSMD14, PSMD2, PSMD3, PSMD5, PSMD6, PSMD7, PSMD8, PSME1, PUS7, SEM1, TCF12, TP53, TP73, UFL1, YTHDF2]* |
| GO:1901532 | regulation of hematopoietic progenitor cell differentiation | 31.25 | 30 | down | *[MIXL1, NUDT21, PSMA2, PSMA3, PSMA4, PSMA5, PSMA8, PSMB3, PSMB5, PSMB6, PSMB7, PSMB9, PSMC2, PSMC4, PSMC6, PSMD13, PSMD14, PSMD2, PSMD3, PSMD5, PSMD6, PSMD7, PSMD8, PSME1, PUS7, SEM1, SOS1, TCF12, TP73, YTHDF2]* |
| GO:0038095 | Fc-epsilon receptor signaling pathway | 17.29729652 | 32 | down | *[BTK, IGKC, IGKV1-17, IGLC1, IGLV1-44, IGLV6-57, PLCG2, PPP3CB, PSMA2, PSMA3, PSMA4, PSMA5, PSMA8, PSMB3, PSMB5, PSMB6, PSMB7, PSMB9, PSMC2, PSMC4, PSMC6, PSMD13, PSMD14, PSMD2, PSMD3, PSMD5, PSMD6, PSMD7, PSMD8, PSME1, SEM1, SOS1]* |
| GO:1902036 | regulation of hematopoietic stem cell differentiation | 34.61538315 | 27 | down | *[PSMA2, PSMA3, PSMA4, PSMA5, PSMA8, PSMB3, PSMB5, PSMB6, PSMB7, PSMB9, PSMC2, PSMC4, PSMC6, PSMD13, PSMD14, PSMD2, PSMD3, PSMD5, PSMD6, PSMD7, PSMD8, PSME1, PUS7, SEM1, TCF12, TP73, YTHDF2]* |
| GO:0050852 | T cell receptor signalling pathway | 16.4383564 | 36 | down | *[ADA, CTLA4, DENND1B, EIF2B1, EIF2B2, EIF2B3, EIF2B5, HRAS, LILRB4, PAWR, PLCG2, PSMA2, PSMA3, PSMA4, PSMA5, PSMA8, PSMB3, PSMB5, PSMB6, PSMB7, PSMB9, PSMC2, PSMC4, PSMC6, PSMD13, PSMD14, PSMD2, PSMD3, PSMD5, PSMD6, PSMD7, PSMD8, PSME1, PTPN2, RAB29, SEM1]* |
| GO:0002223 | stimulatory C-type lectin receptor signalling pathway | 22.31404877 | 27 | down | *[HRAS, ICAM2, NRAS, PLCG2, PSMA2, PSMA3, PSMA4, PSMA5, PSMA8, PSMB3, PSMB5, PSMB6, PSMB7, PSMB9, PSMC2, PSMC4, PSMC6, PSMD13, PSMD14, PSMD2, PSMD3, PSMD5, PSMD6, PSMD7, PSMD8, PSME1, SEM1]* |
| GO:0030097 | hemopoiesis | 12.08677673 | 117 | up | *[ACTN1, ACVR2A, ADGRG3, AGO3, AGO4, AHSP, ANGPT1, ANXA1, BRAF, C17orf99, CA2, CD4, CDK13, CEBPB, CLCF1, CR1, CREB1, CREBBP, CRIP2, CTNNB1, CTNNBIP1, CUL4A, ERCC1, F2RL1, FAM210B, FAXDC2, FBXO7, FLT3LG, FLVCR1, FNIP1, FOS, FOXP1, G6PD, GAB3, GATA1, GATA2, HDAC5, HHEX, HIPK1, IL4R, IRF2BP2, ITPKB, JUNB, KAT6A, KAT8, KLF1, KMT2D, L3MBTL3, LEPROT, LYN, MFHAS1, MKNK2, MPL, MS4A1, MTURN, N4BP2L2, NCKAP1L, NDFIP1, NLRP3, NOTCH1, OSM, PBX1, PICALM, PIK3CD, PIK3R1, PIM1, PIP4K2A, PLCL2, PREX1, PRKCA, PSEN1, PSME4, PSMF1, PTBP3, PTGER4, PTPRC, RASSF2, RBPJ, RC3H1, RCOR1, ROGDI, RPL22, SGPL1, SH2B3, SIN3A, SLC25A38, SLC4A1, SMAD7, SNRK, SNX10, SOS2, SP3, ST3GAL1, STAT5B, STK11, STK4, TAZ, TESC, TET2, TGFBR2, TMEM64, TMEM91, TNRC6B, TNRC6C, TRAF6, TRIM10, TSPAN2, TYROBP, VSIR, WASF2, YY1, ZBTB1, ZBTB7A, ZFP36, ZFP36L1, ZFP36L2, ZMIZ1]* |
| GO:0030099 | myeloid cell differentiation | 15.05618 | 67 | up | *[ACTN1, ACVR2A, AGO3, AGO4, AHSP, CA2, CD4, CEBPB, CREB1, CREBBP, CTNNB1, CTNNBIP1, CUL4A, F2RL1, FAM210B, FAXDC2, FLVCR1, FOS, FOXP1, G6PD, GAB3, GATA1, GATA2, ITPKB, JUNB, KAT6A, KAT8, KLF1, KMT2D, L3MBTL3, LYN, MFHAS1, MPL, MTURN, NCKAP1L, NDFIP1, PIK3CD, PIK3R1, PIP4K2A, PRKCA, PSEN1, PTBP3, RASSF2, RBPJ, RCOR1, SH2B3, SIN3A, SLC25A38, SLC4A1, SNRK, SNX10, SP3, STAT5B, TESC, TET2, TGFBR2, TMEM64, TNRC6B, TNRC6C, TRAF6, TRIM10, TSPAN2, TYROBP, WASF2, ZBTB7A, ZFP36, ZFP36L1]* |

Table-S6: Gene set enrichment involved in immune system analysis of DEGs for the functioning of immune system.
