# Supplementary material for: Analysis of gene expression profile for identification of novel gene signatures during dengue infection
Source: Infect Med (Beijing). 2023 Feb 18;2(1):19–30. doi: 10.1016/j.imj.2023.02.002 (PMC10699721; doi:10.1016/j.imj.2023.02.002)
Supplement: Supplementary file 2 [file mmc2.docx]

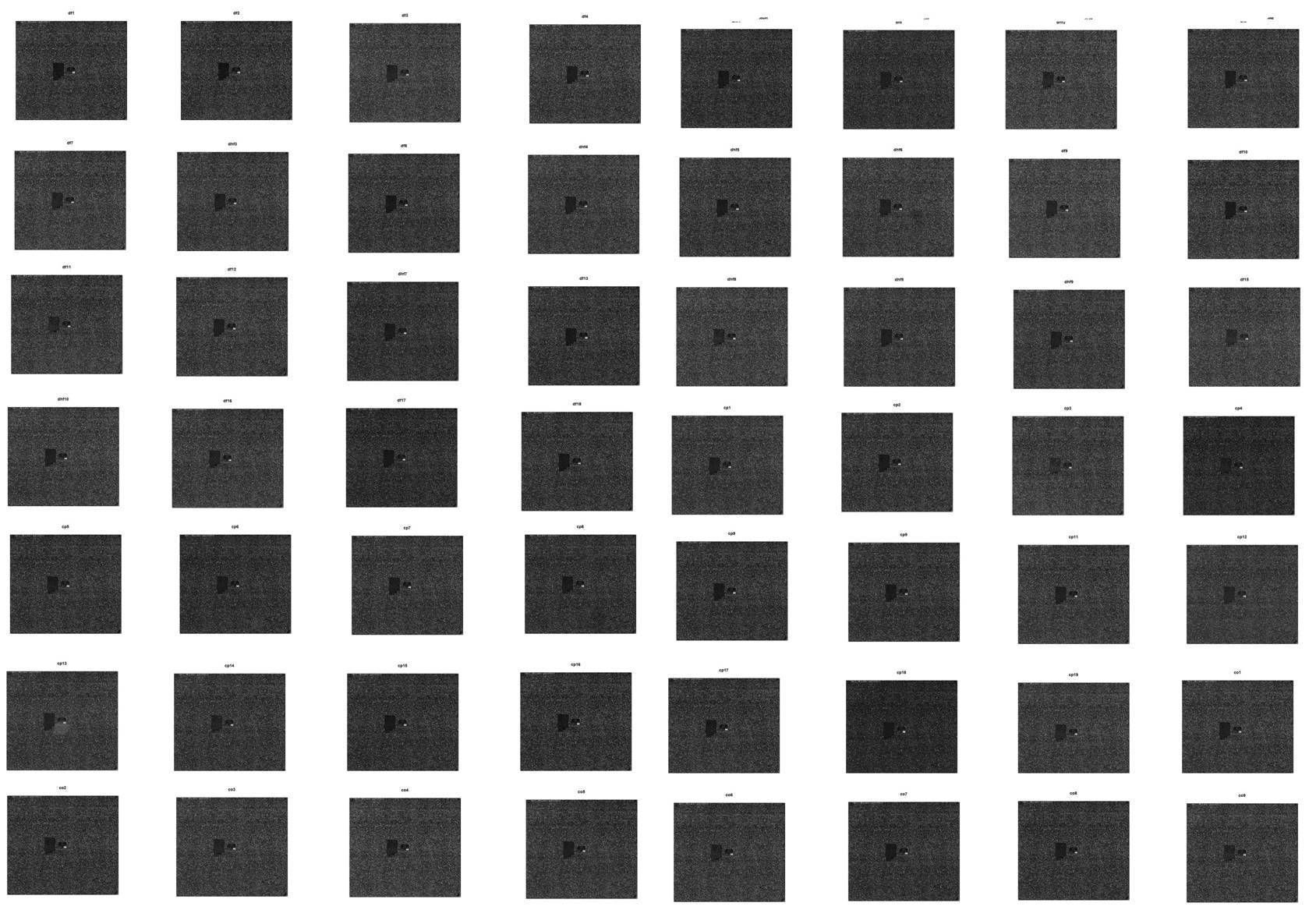


Figure-S1: Gene expression data at pre-processing stage, the 56 quality images showing many spots with different intensities.


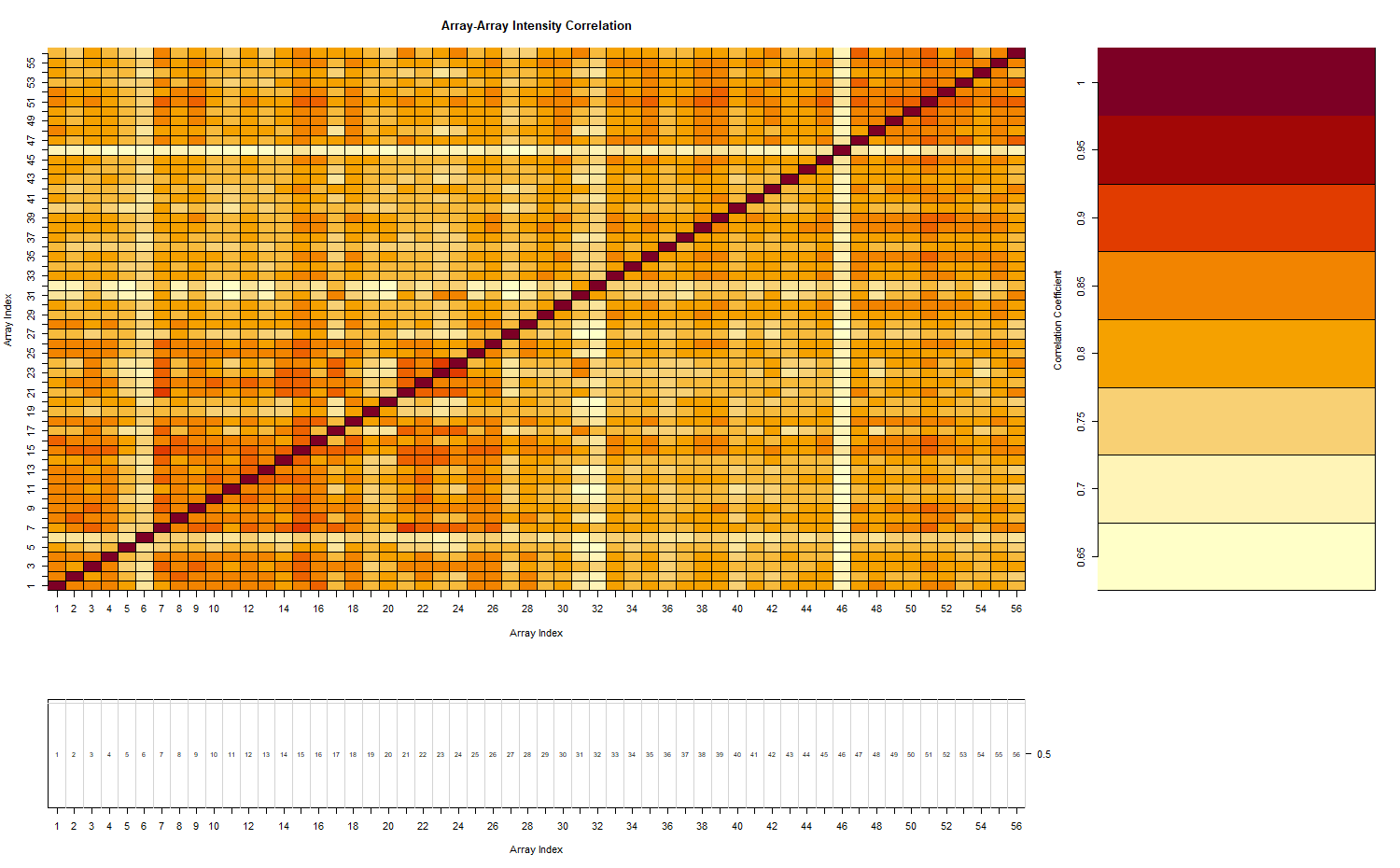


Figure-S2: Visualization of Array-array intensity correlation plot between 56 samples.


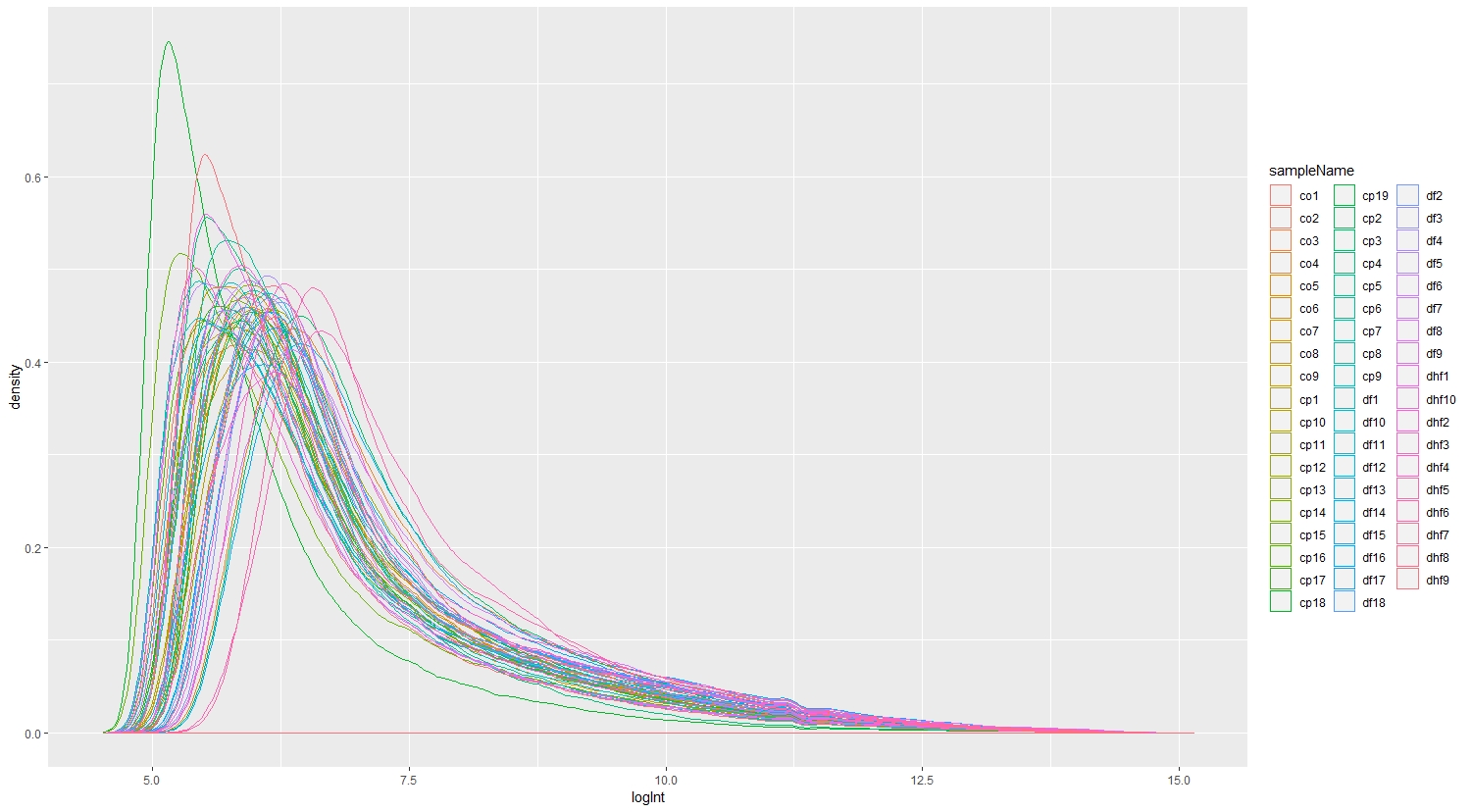


Figure-S3: Density plot of probe signals on 56 different arrays.


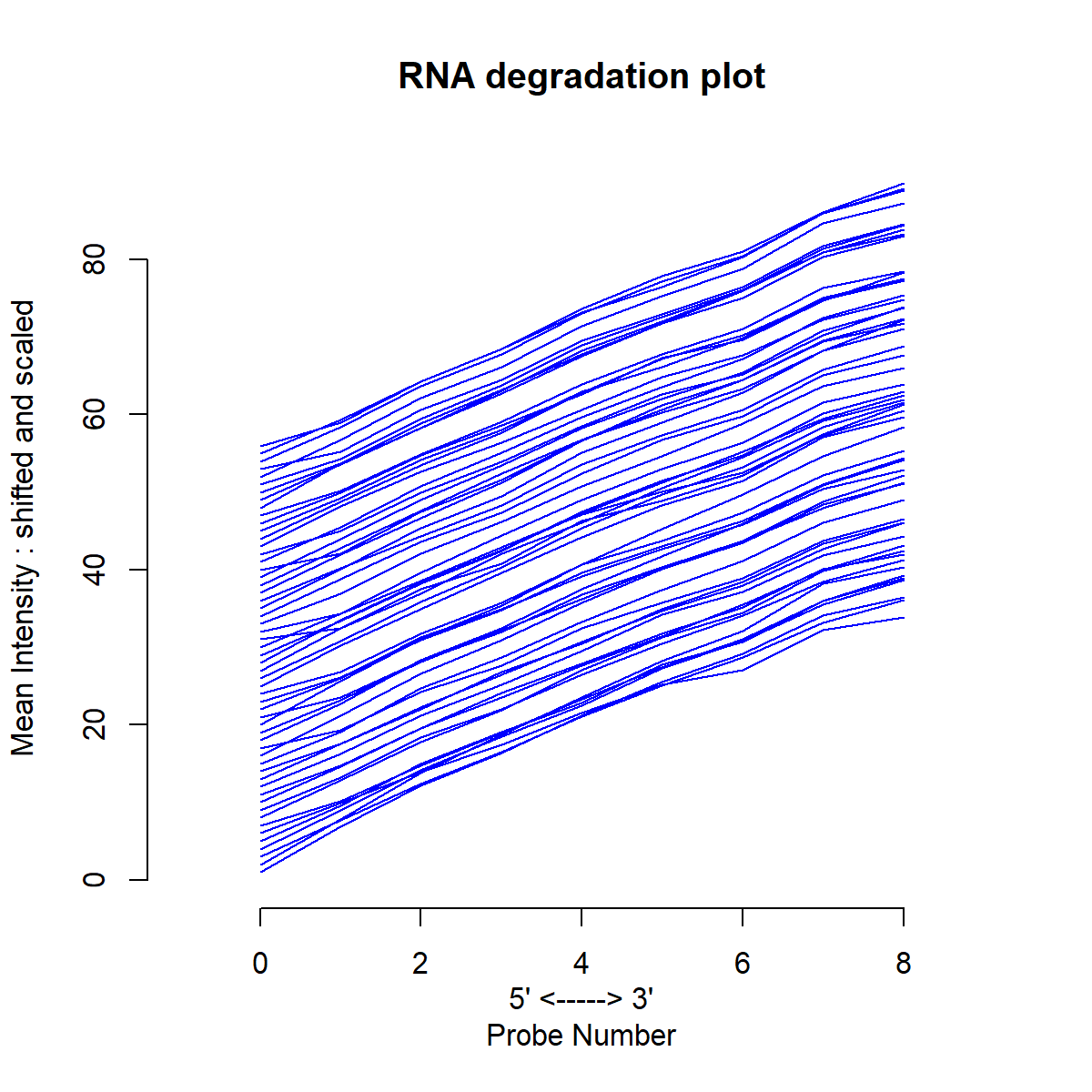


Figure-S4: RNA degradation plot shows 56 arrays give a curve with a regular and comparable slope.


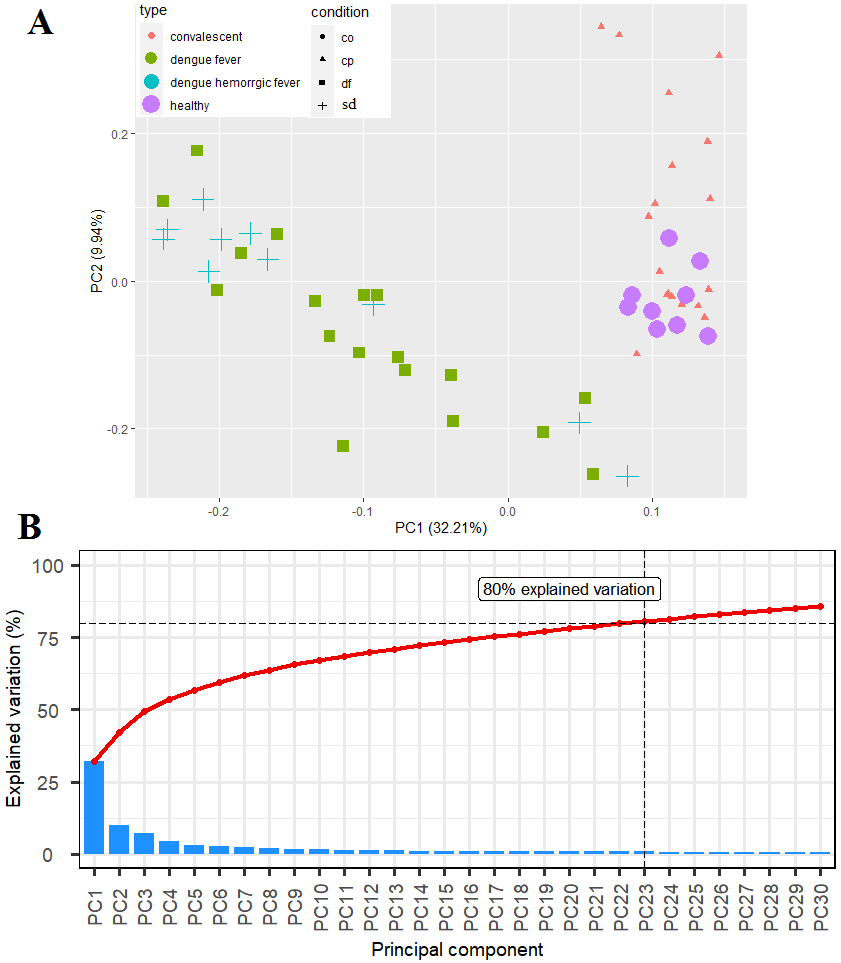


Figure-S5: Principal component analysis 3-dimensional scatter plot represent the differential gene expression patterns of three clinical forms of Dengue (Dengue fever, convalescent, severe dengue) along with Healthy. B) Scree plot showing number of factors in exploratory analysis of Dengue data.

1. **df-c (down regulated genes (Probe ID)):**


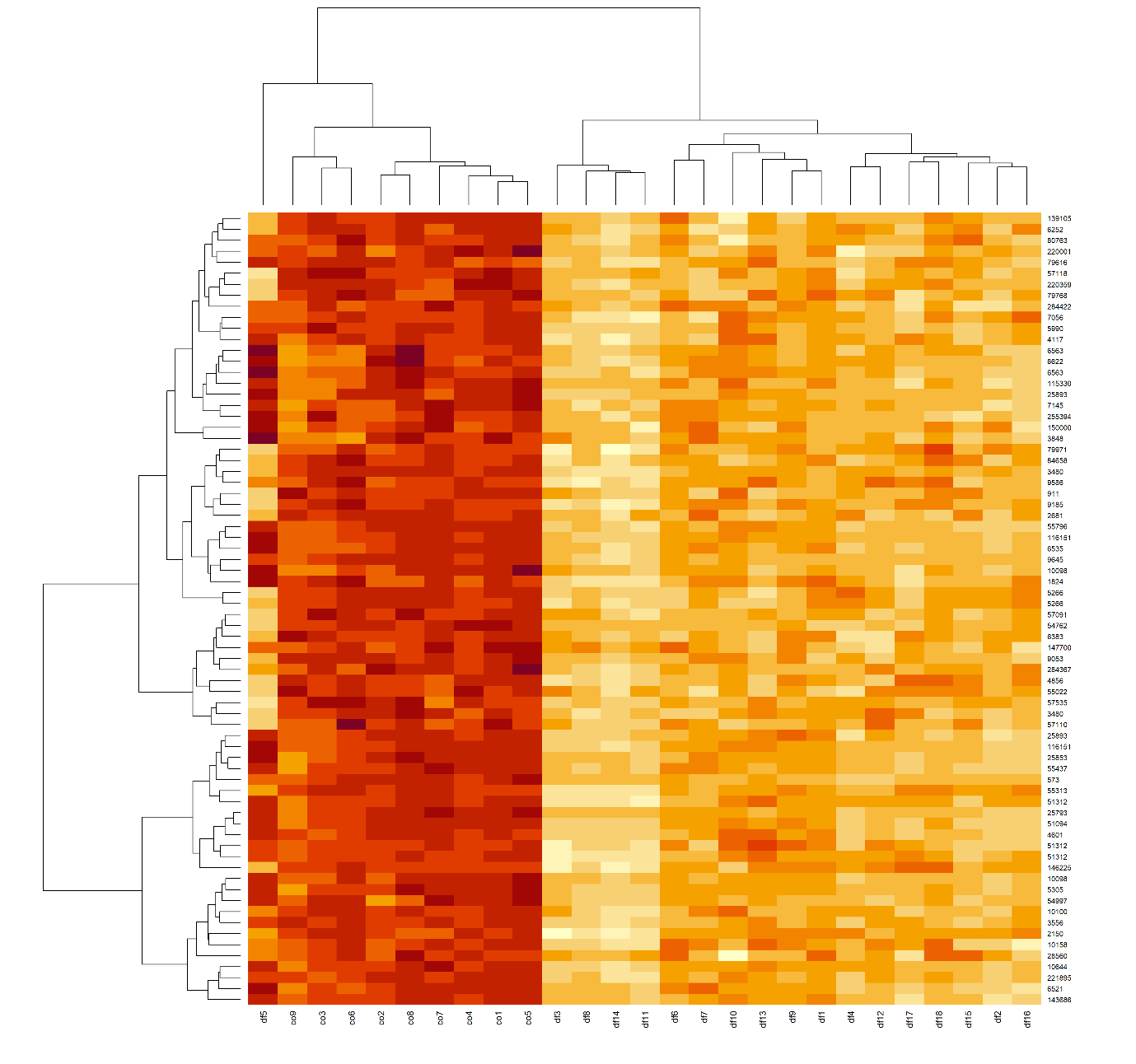


Figure-S6(A-F): Hierarchical cluster analysis of top 200 DEGs (up-regulated and down regulated) between different clinical groups.

1. **df-c (Up regulated genes):**

**
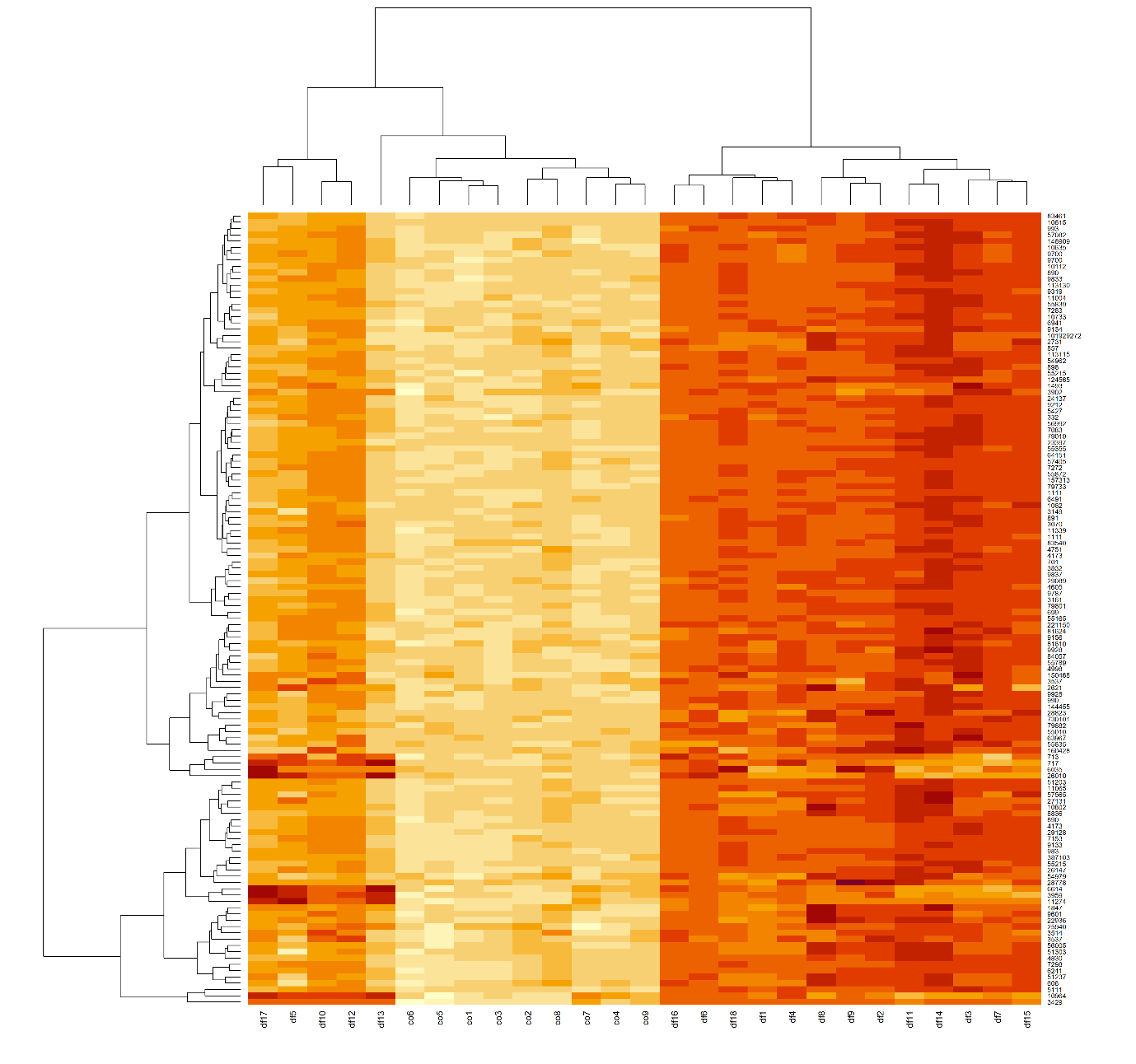
**

1. **cp-df (down-regulated genes):**

**
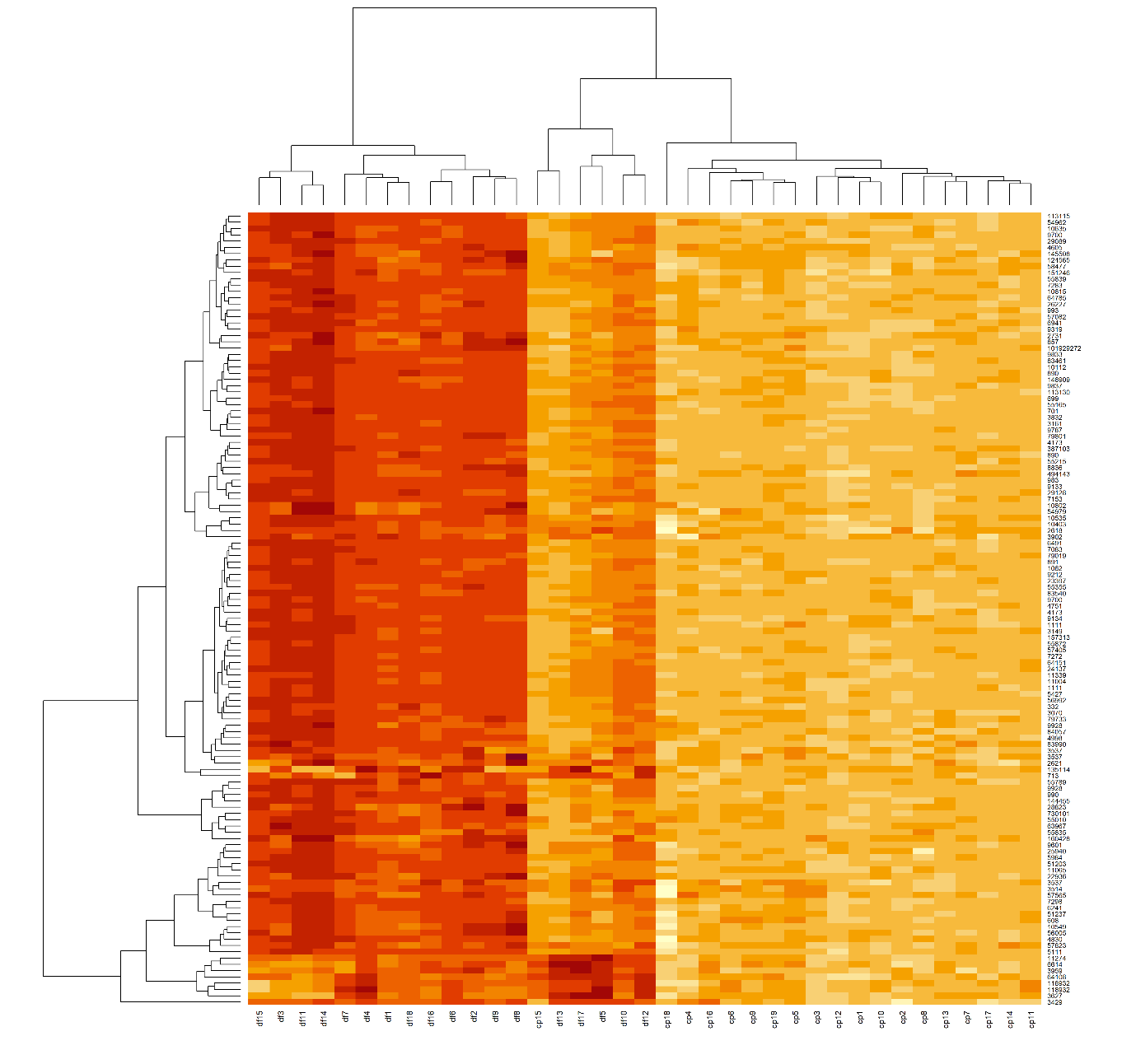
**

1. **cp-df (up-regulated genes):**

**
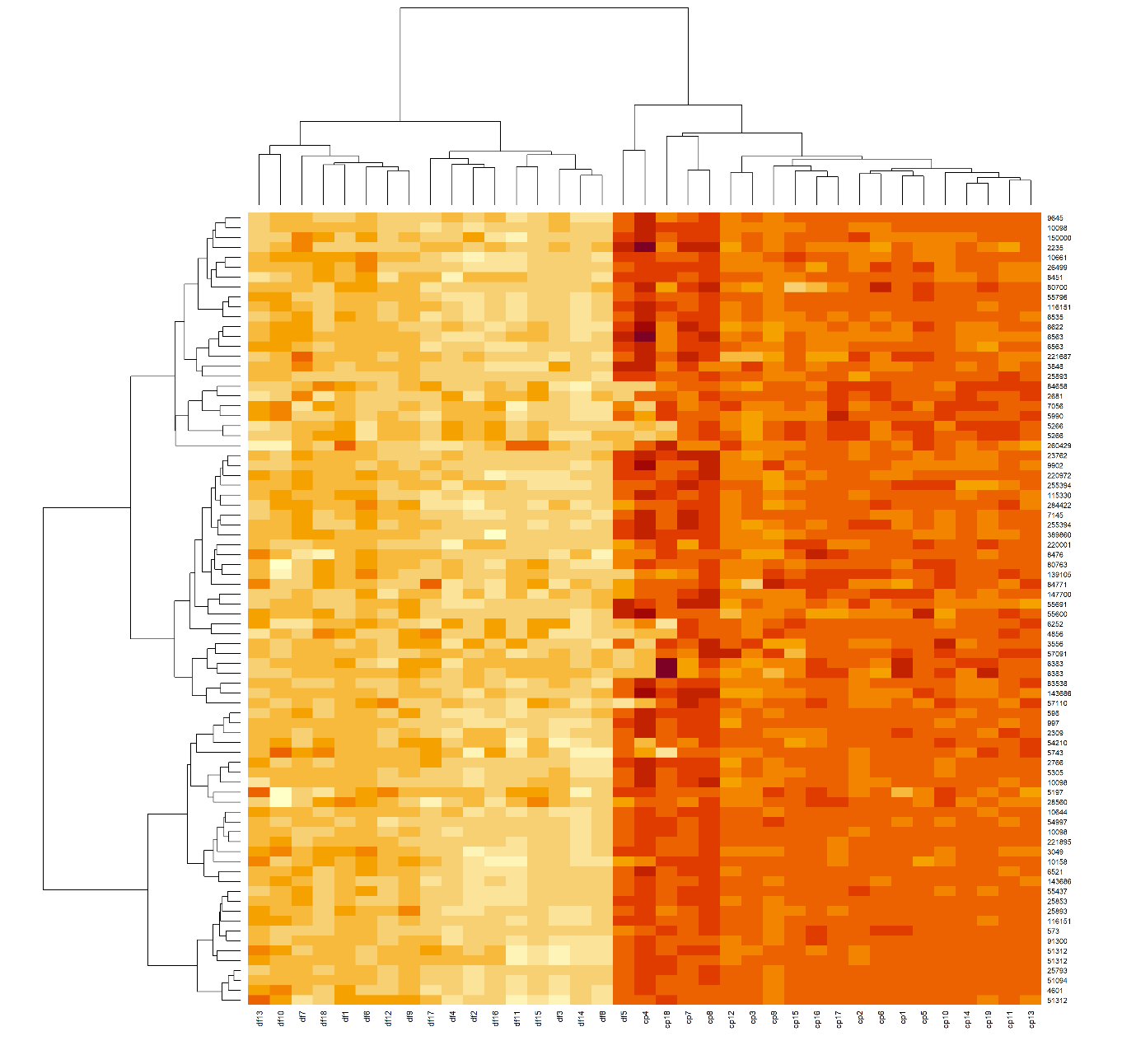
**

1. **cp-dhf (Up regulated genes):**

**
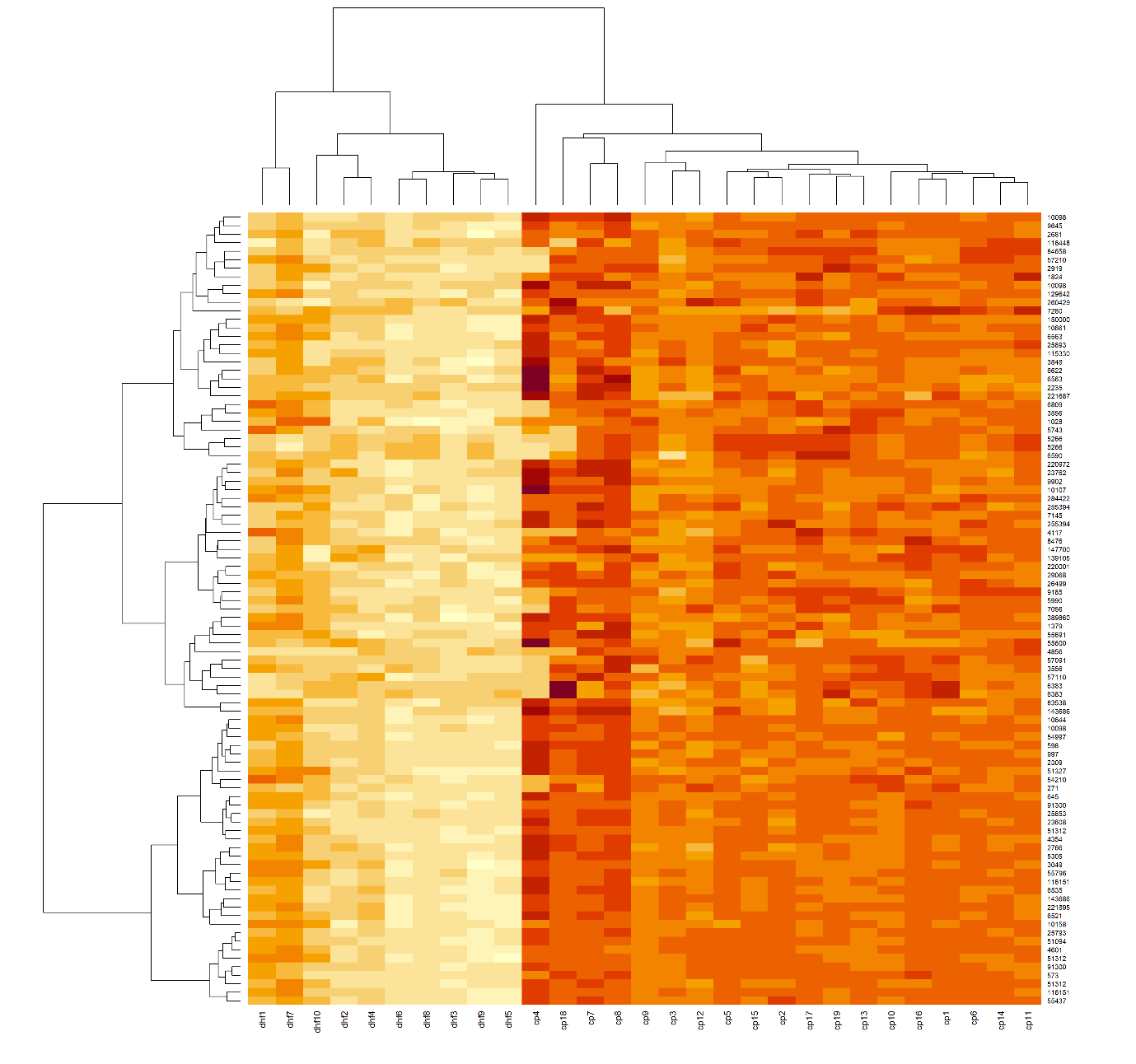
**

1. **cp-dhf (downregulated genes):**

**
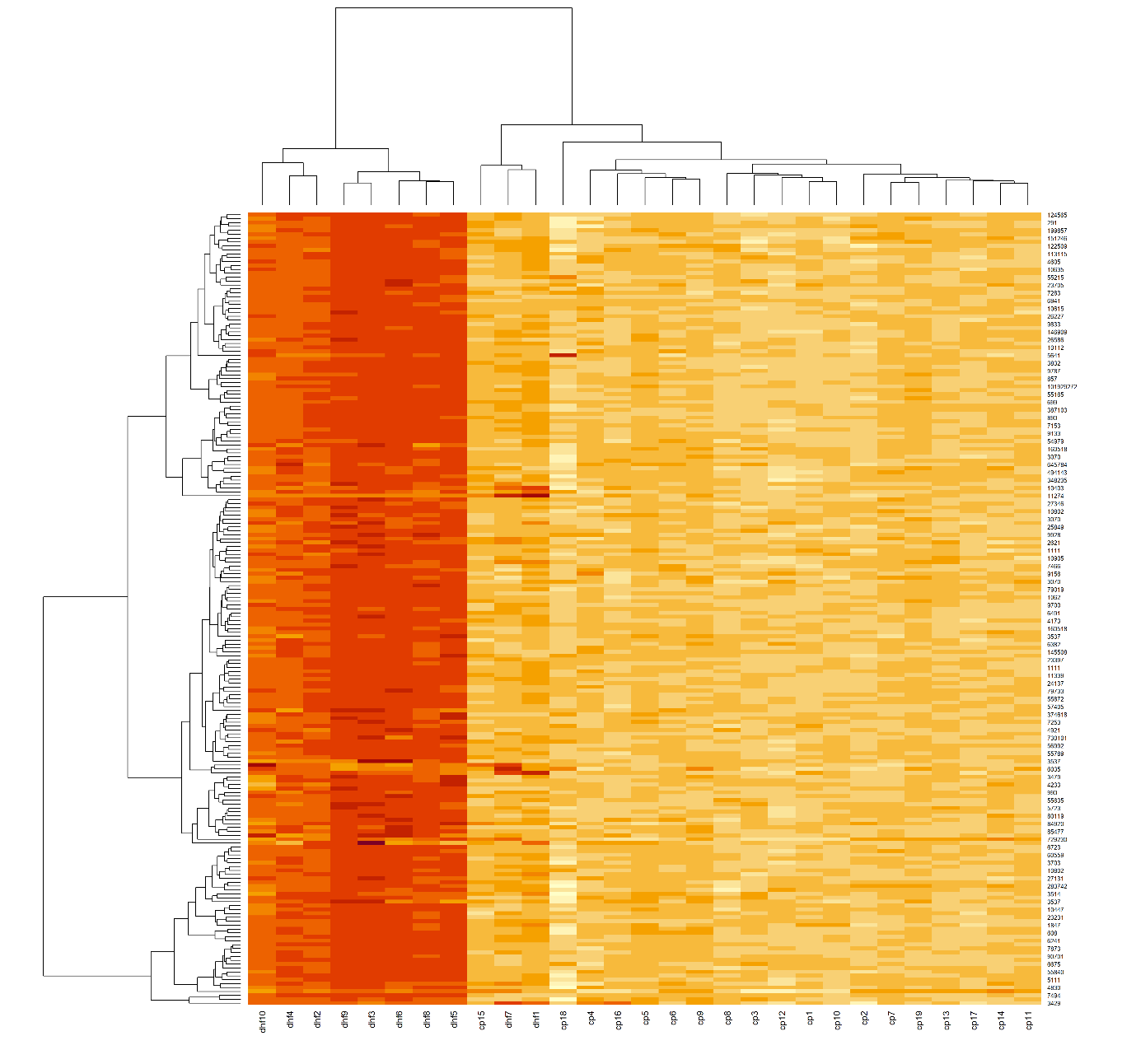
**

1. **dhf-cp (cellular component)**

**
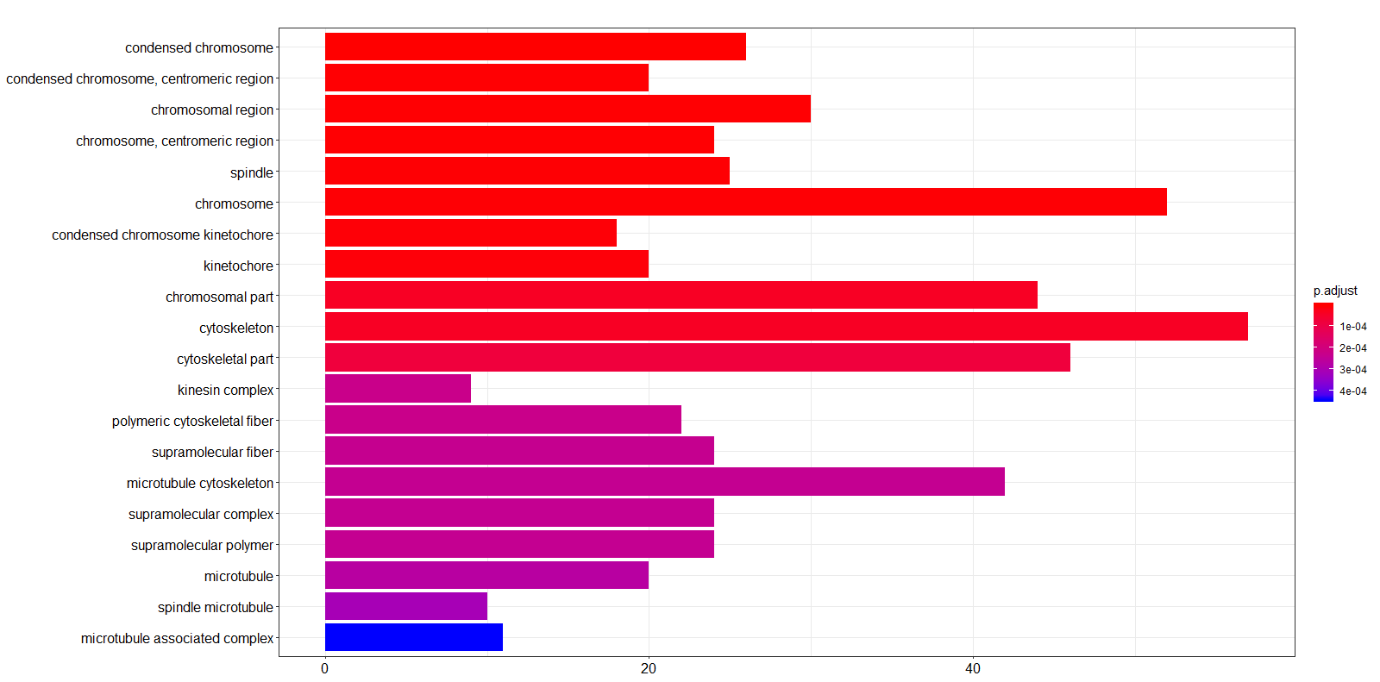
**

Figure-S7 (A-M): Gene ontology (GO) enrichment analysis showing most enriched GO terms are cellular component, biological processes and molecular function. The x-axis represents the number of DEGs enriched terms. Y-axis represents the GO terms.

1. **dhf-cp (Molecular function)**

**
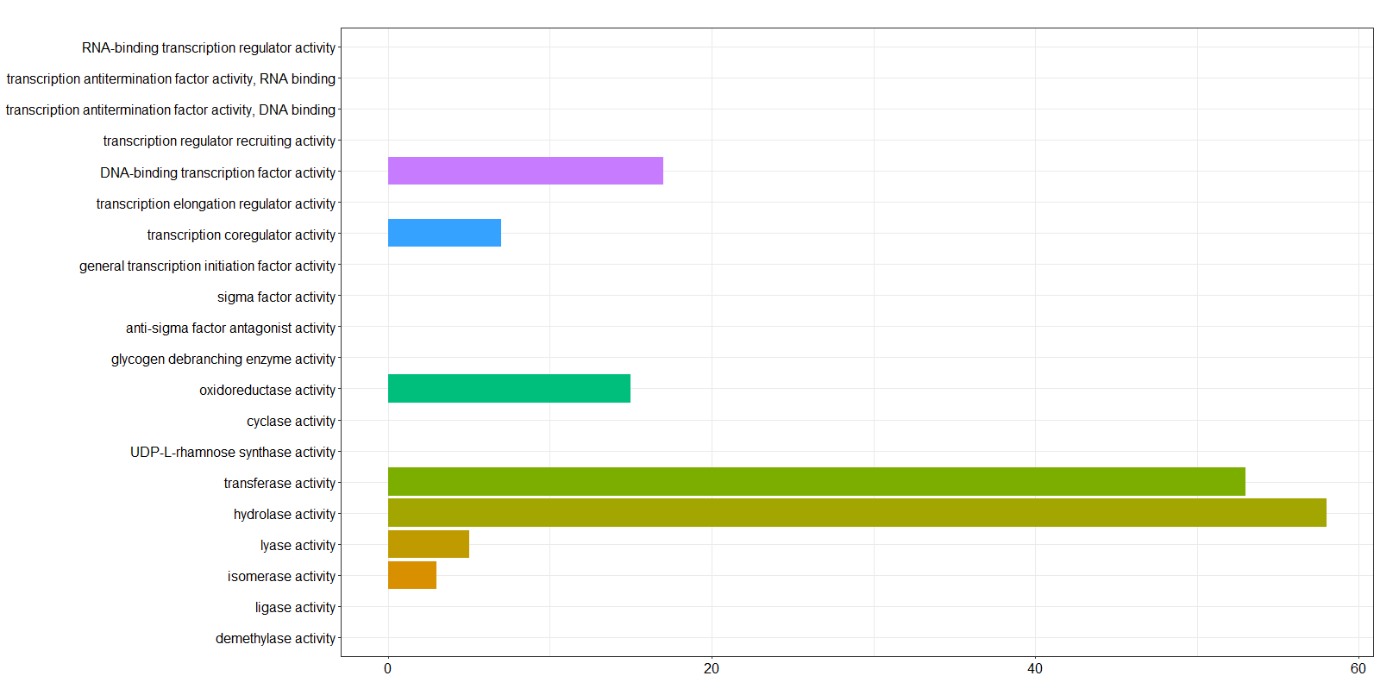
**

1. **dhf-cp (Biological activity)**

**
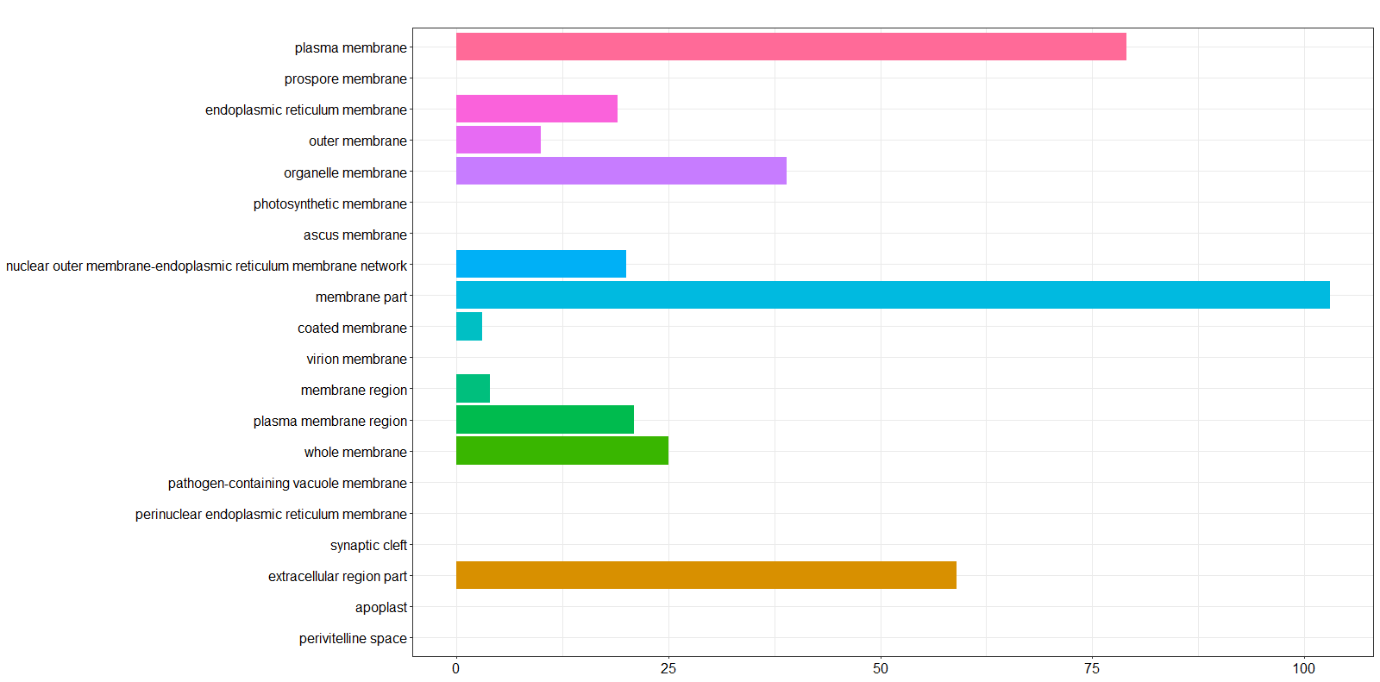
**

1. **df-co (cellular component)**
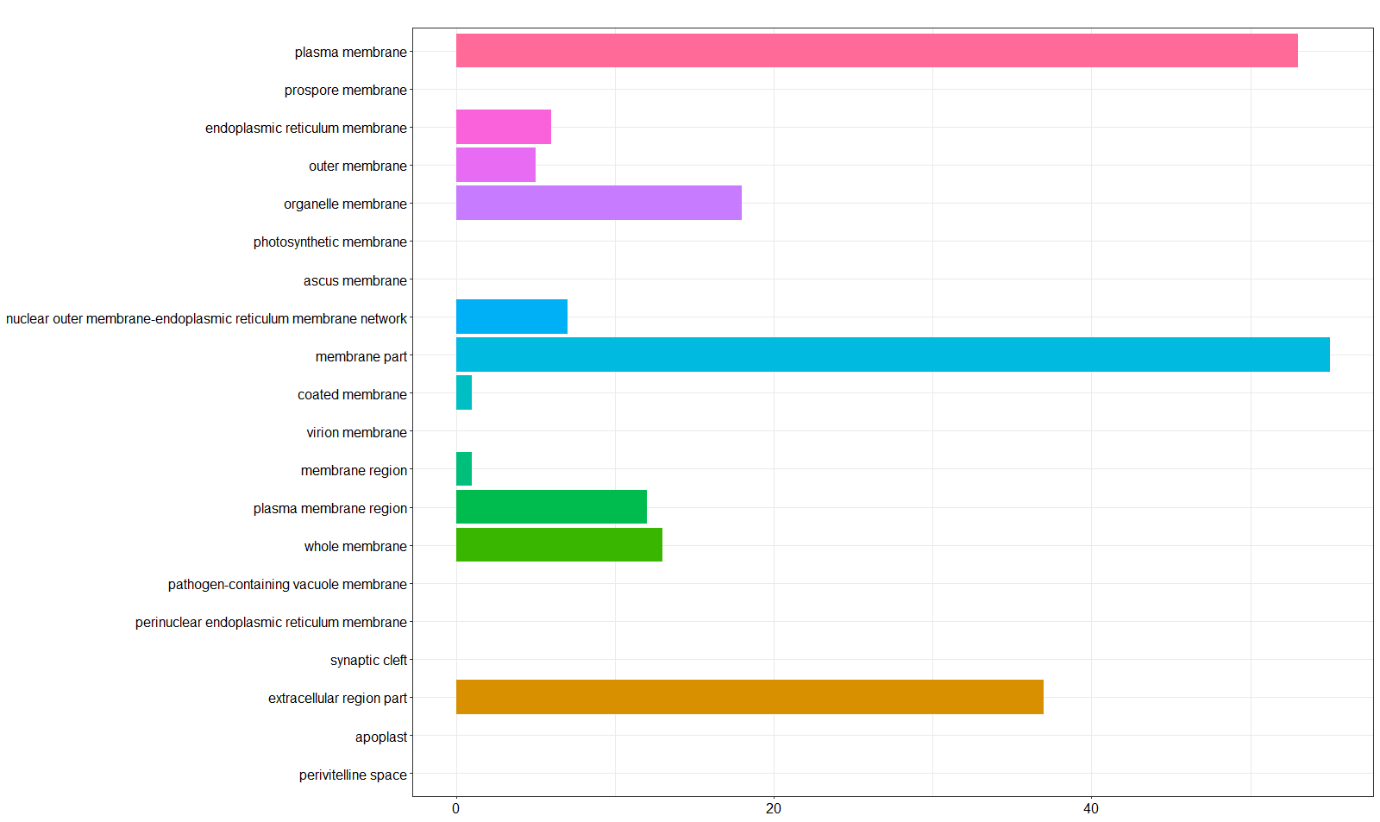

2. **df-co (Molecular function)**

**
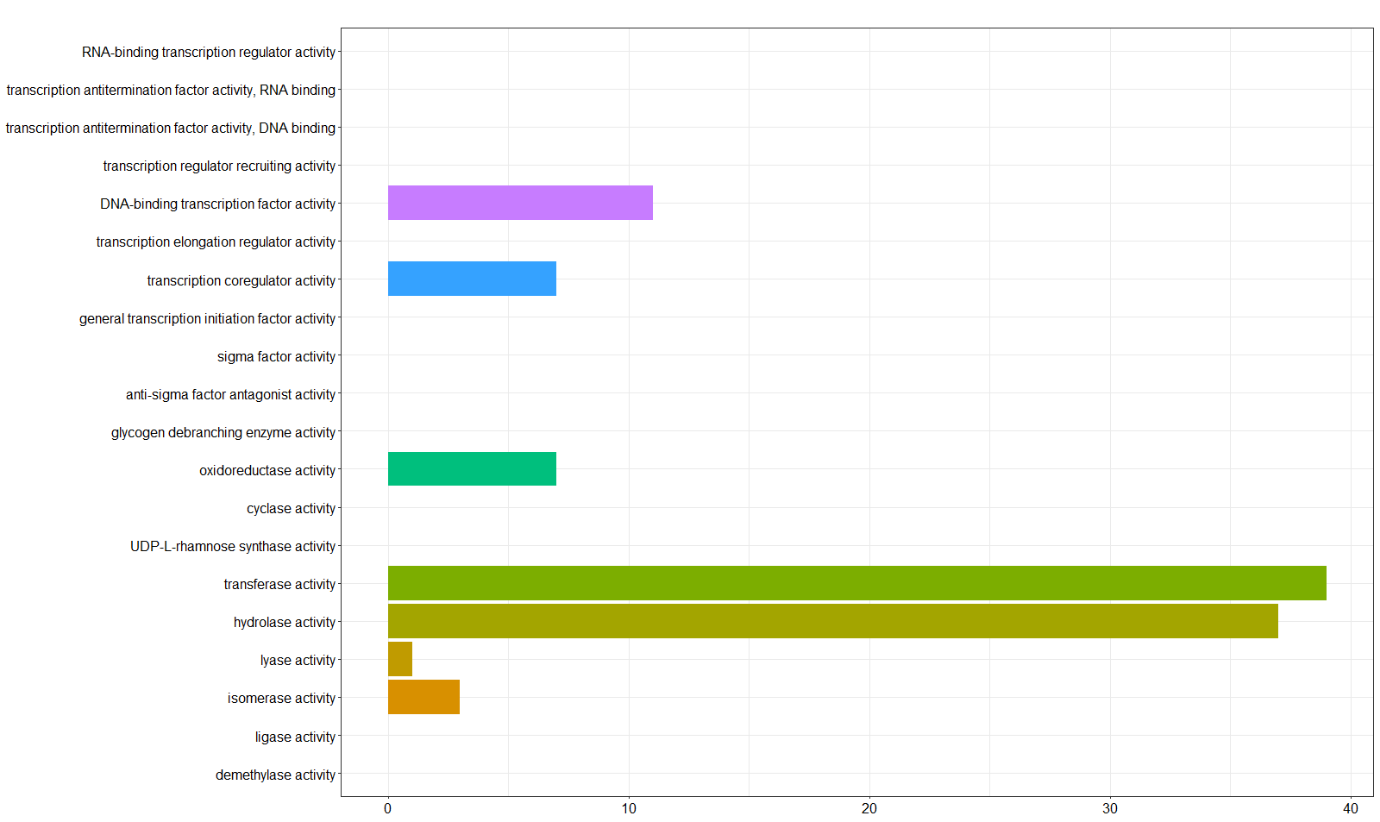
**

1. **df-co (Biological function)**

**
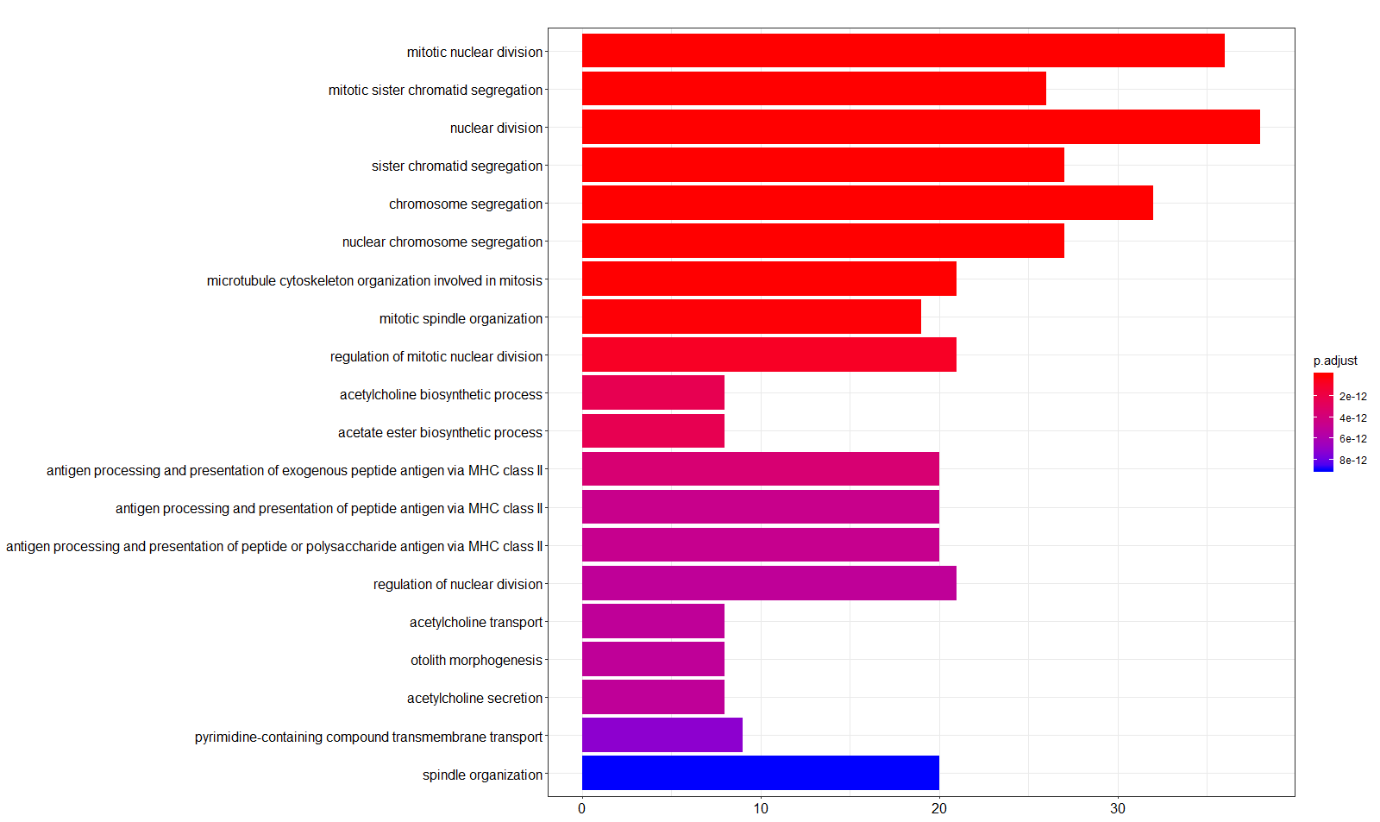
**

1. **cp-df (Biological process):**

**
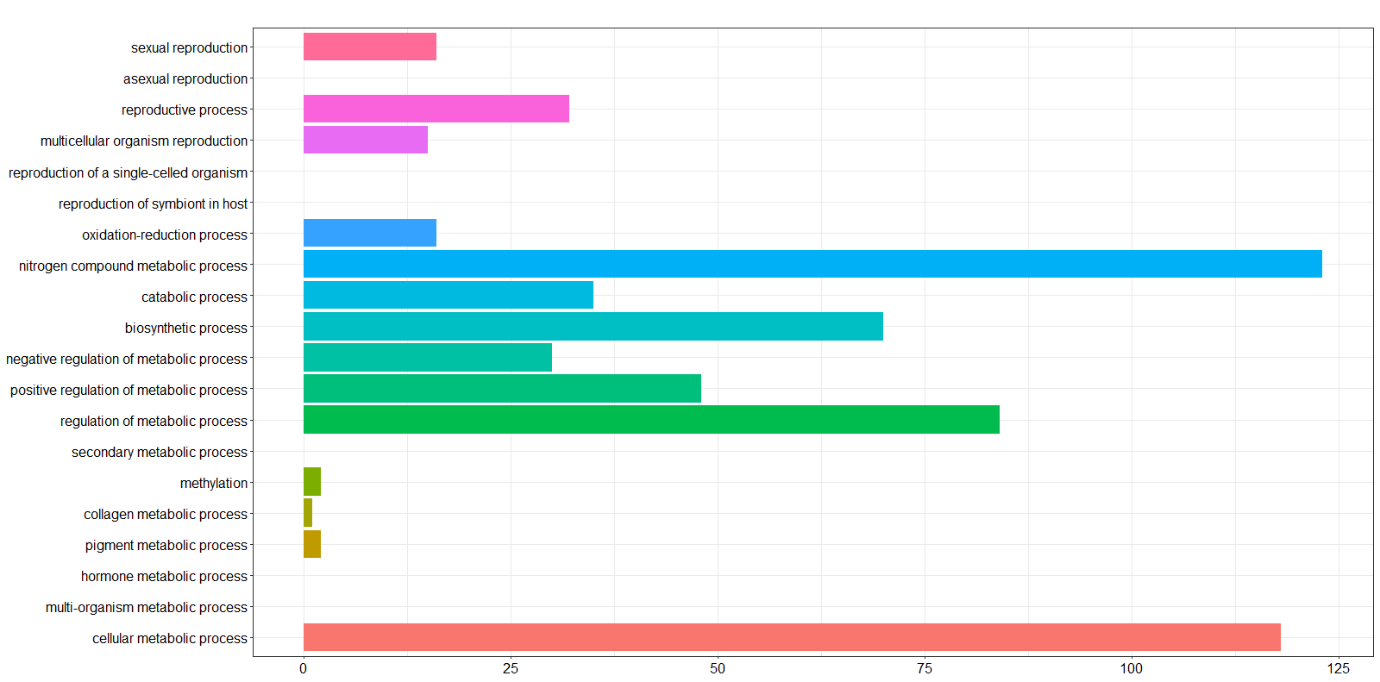
**

1. **cp-df (cellular component):**

**
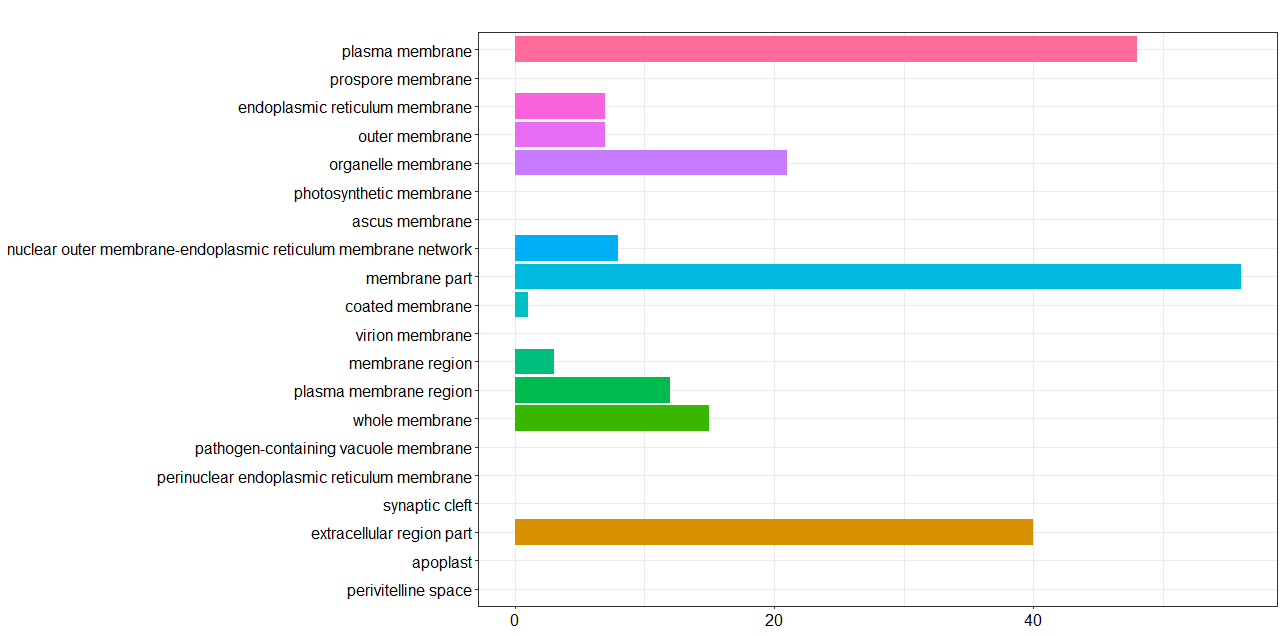
**

1. **cp-df (Molecular function):**

**
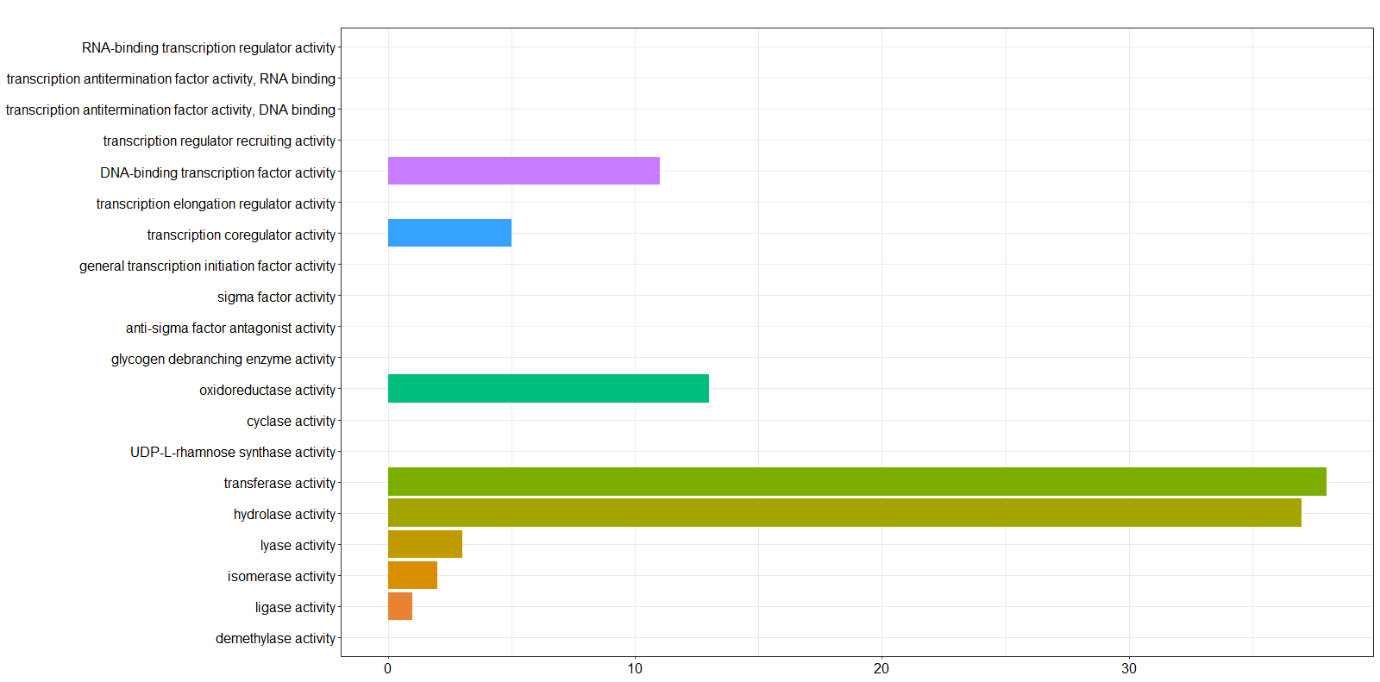
**

1. **cp-dhf (Biological function)**

**
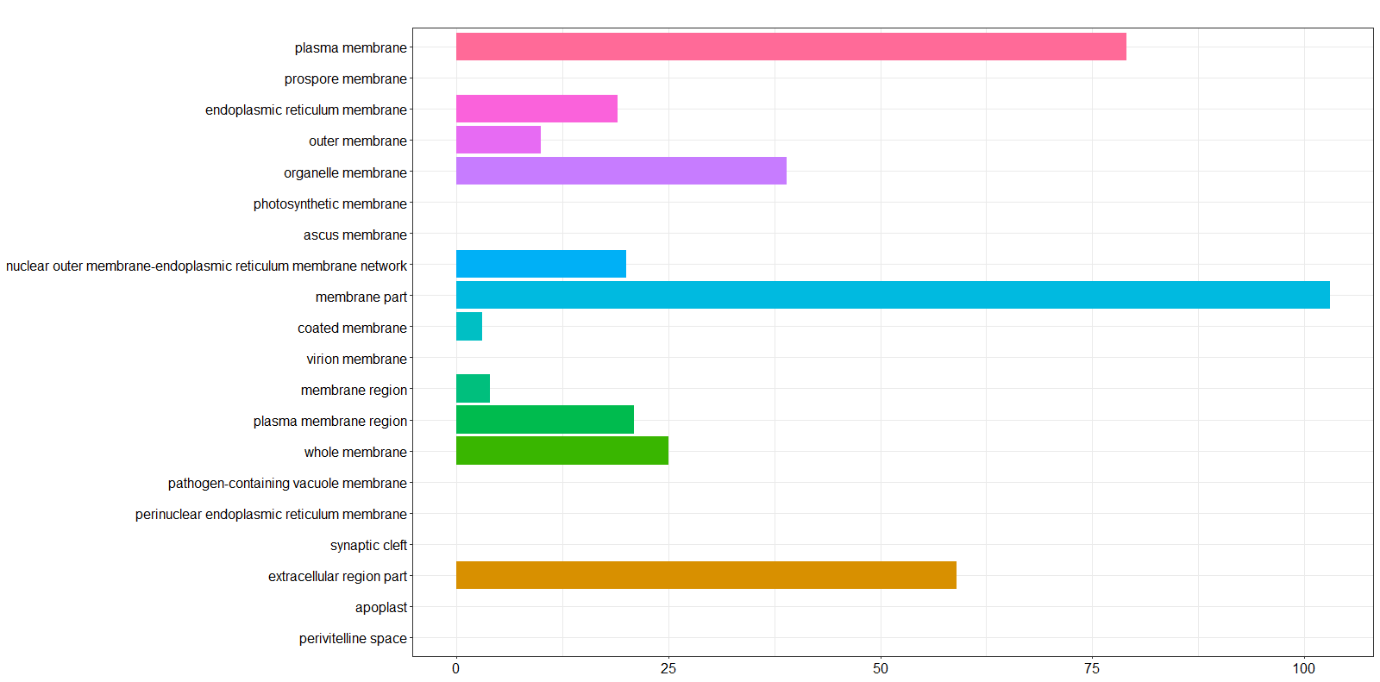
**

1. **cp-dhf (molecular function)**

**
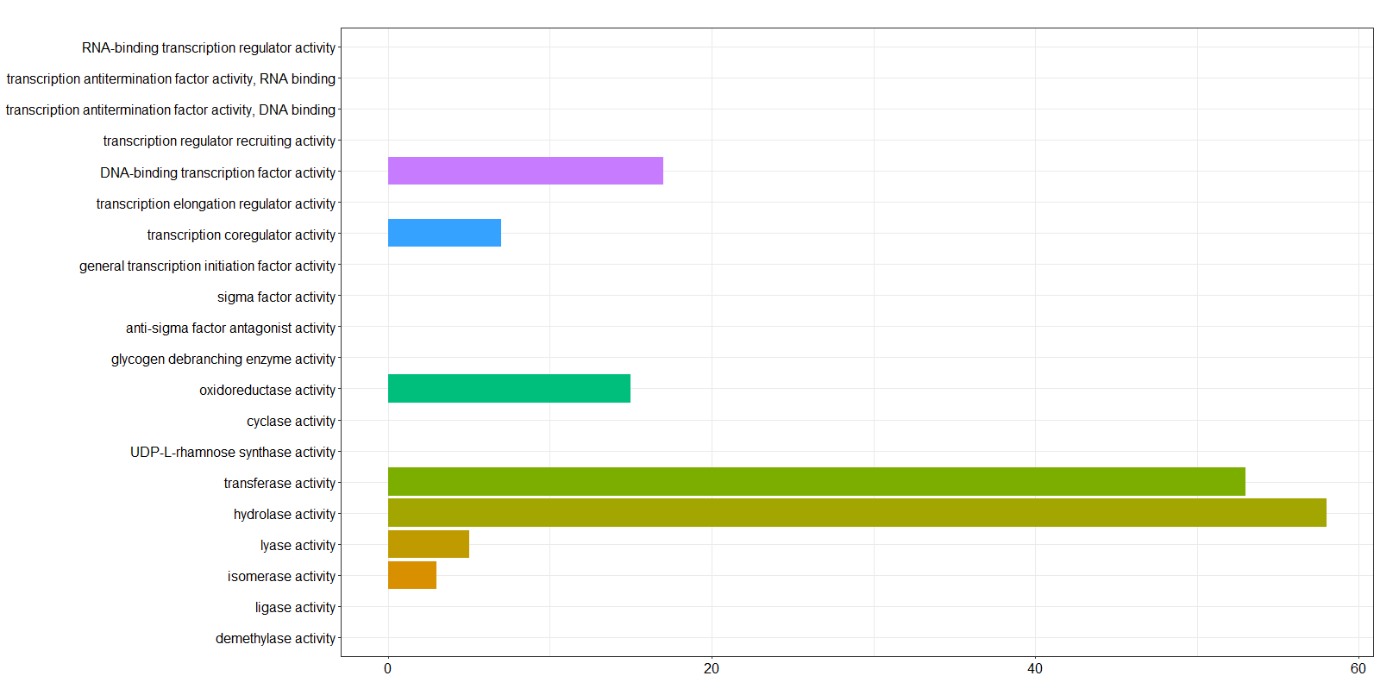
**

1. **cp-dhf (cellular component)**

**
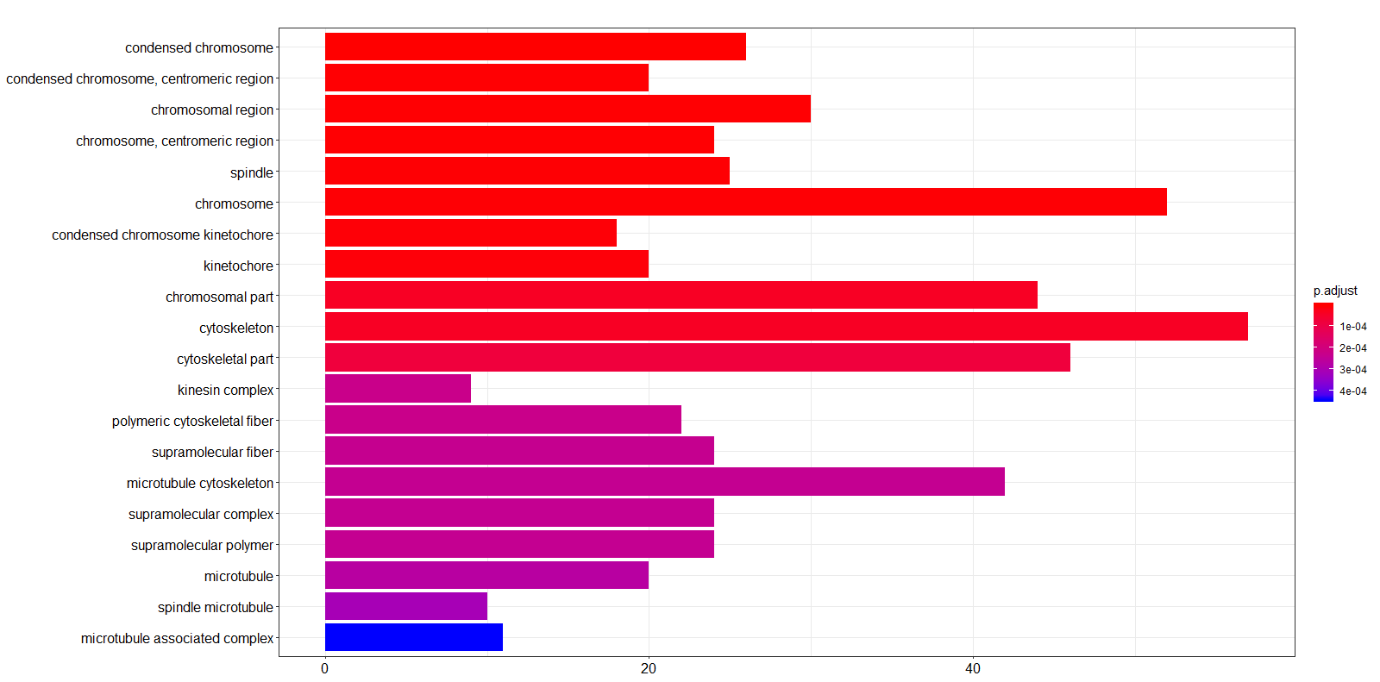
**

1. **dhf-co (cellular component)**

**
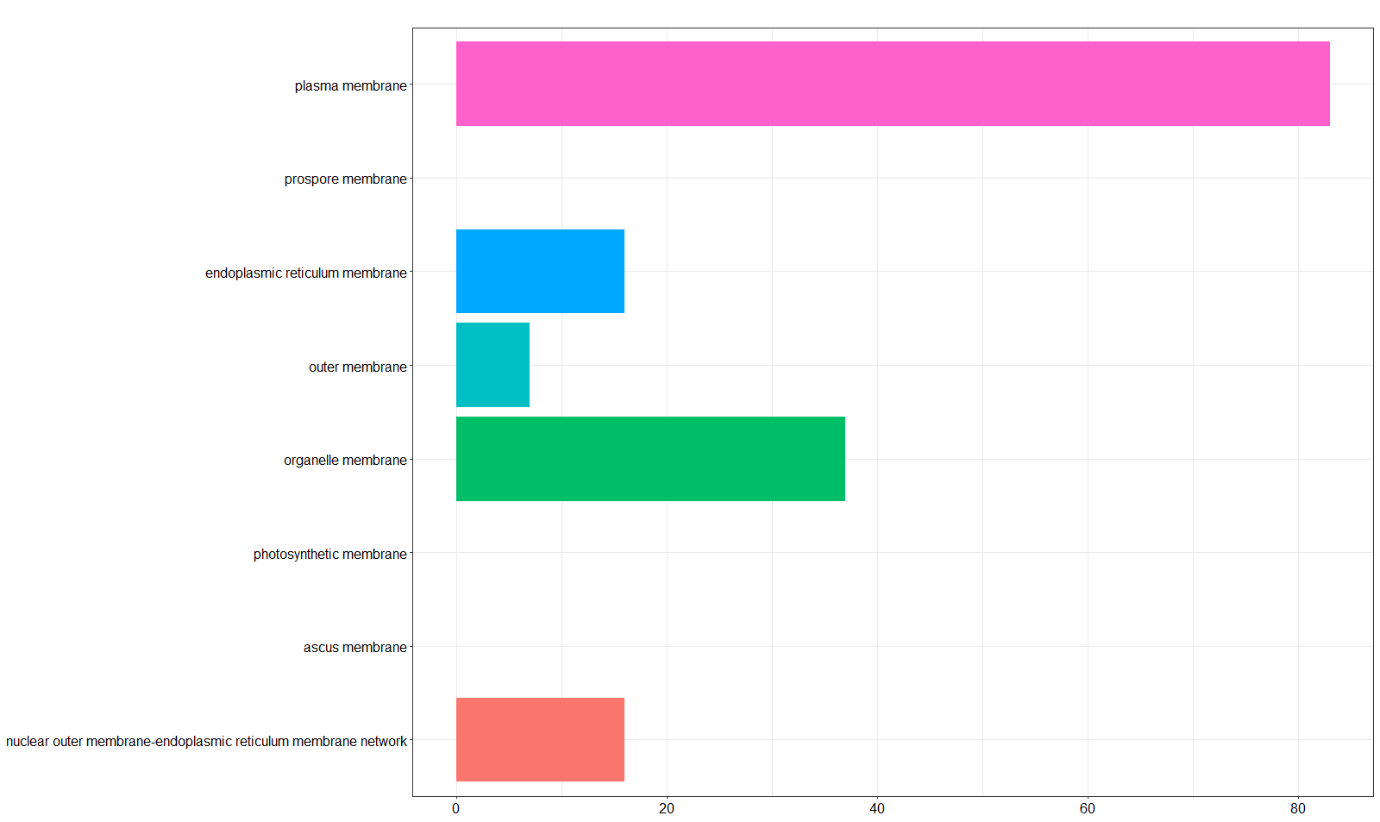
**


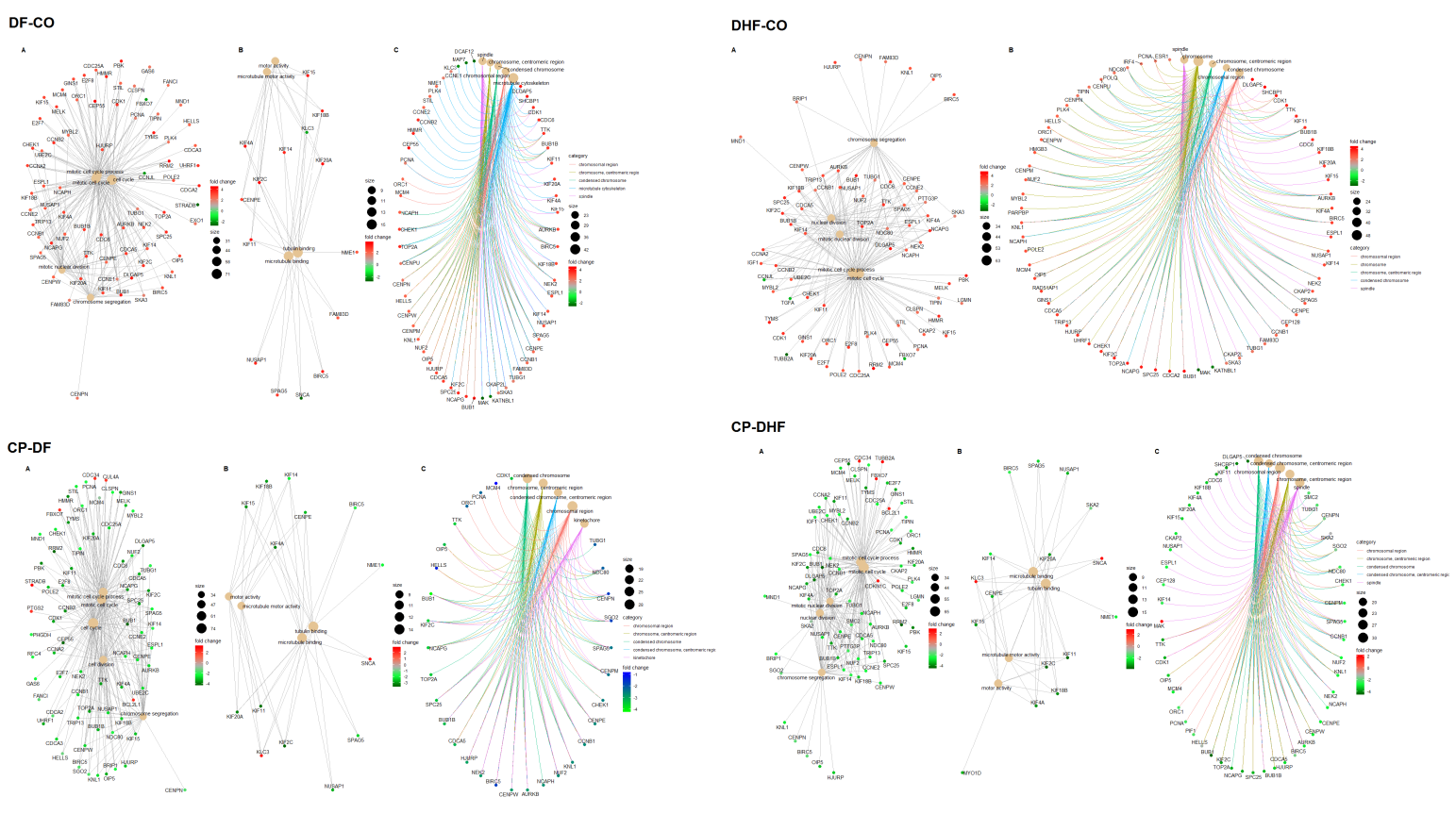


Figure-S8: Gene ontology analysis in different clinical conditions using network plot.


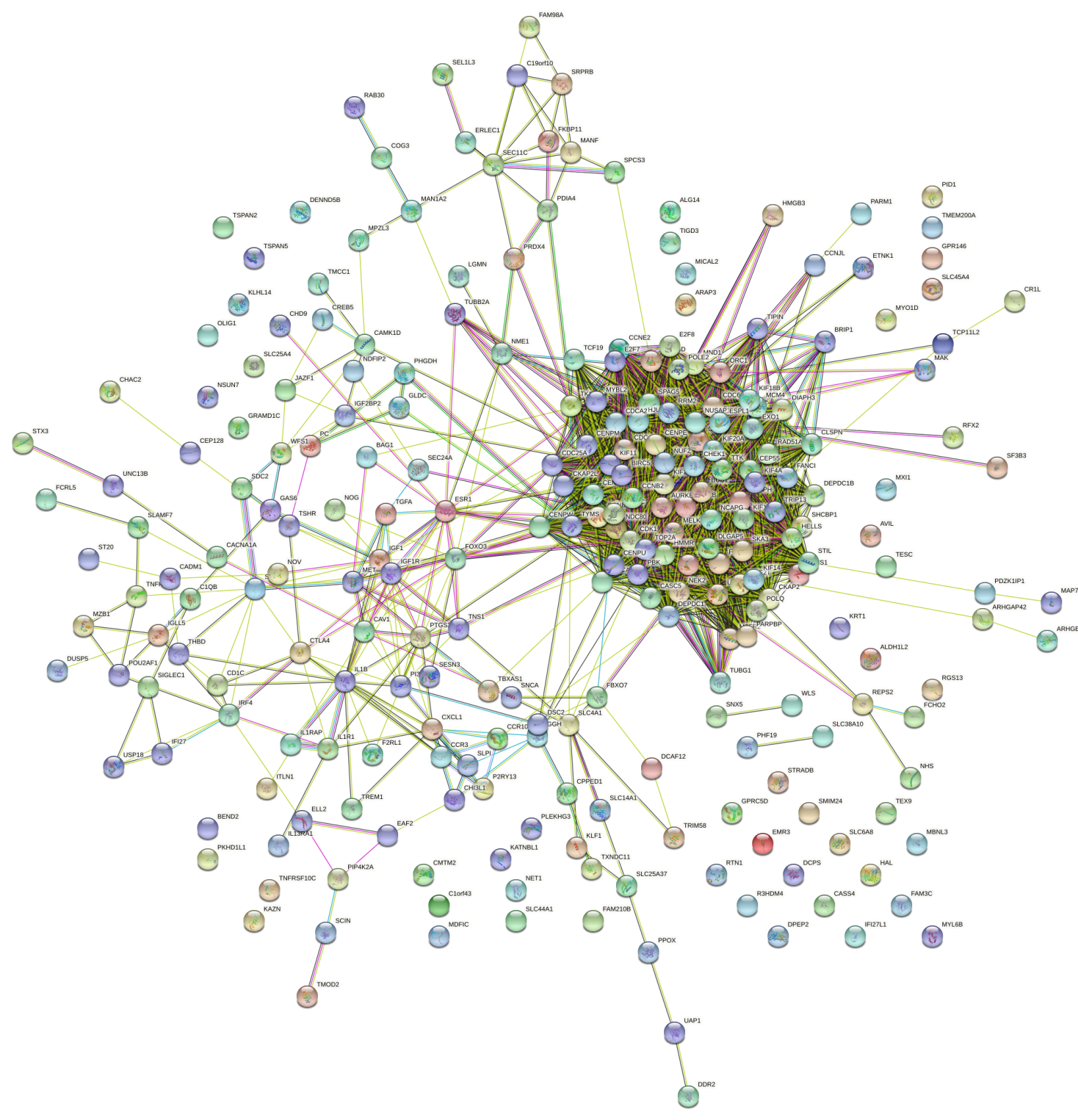


Figure-S9: STRING protein-protein interaction networks for the characterization of DEGs in DF-CO, SD-CO, CP-DF & CP-SD.


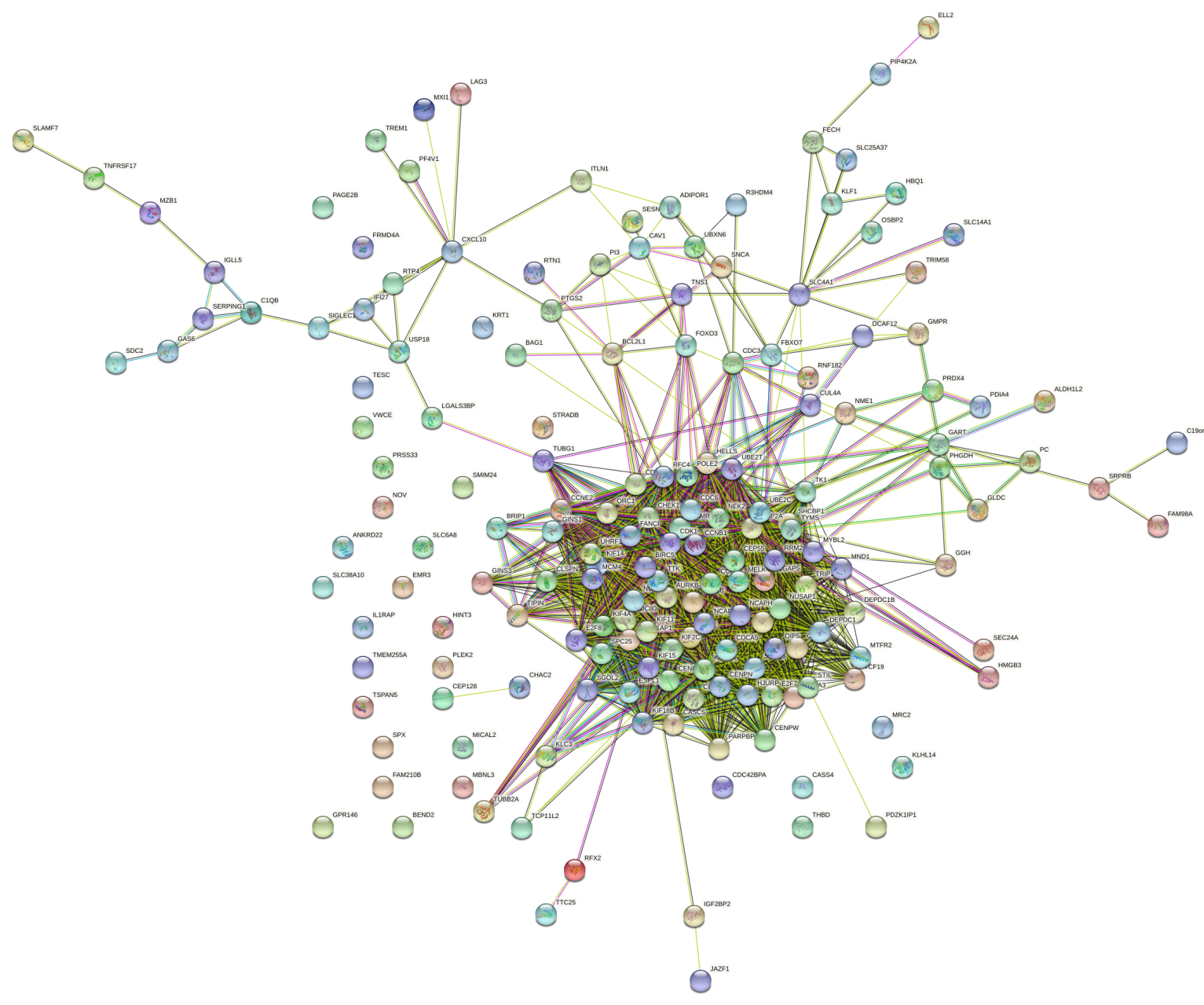


Figure-S10: STRING protein-protein interaction networks for the characterization of DEGs in DF-CO, SD-CO, CP-DF & CP-SD.


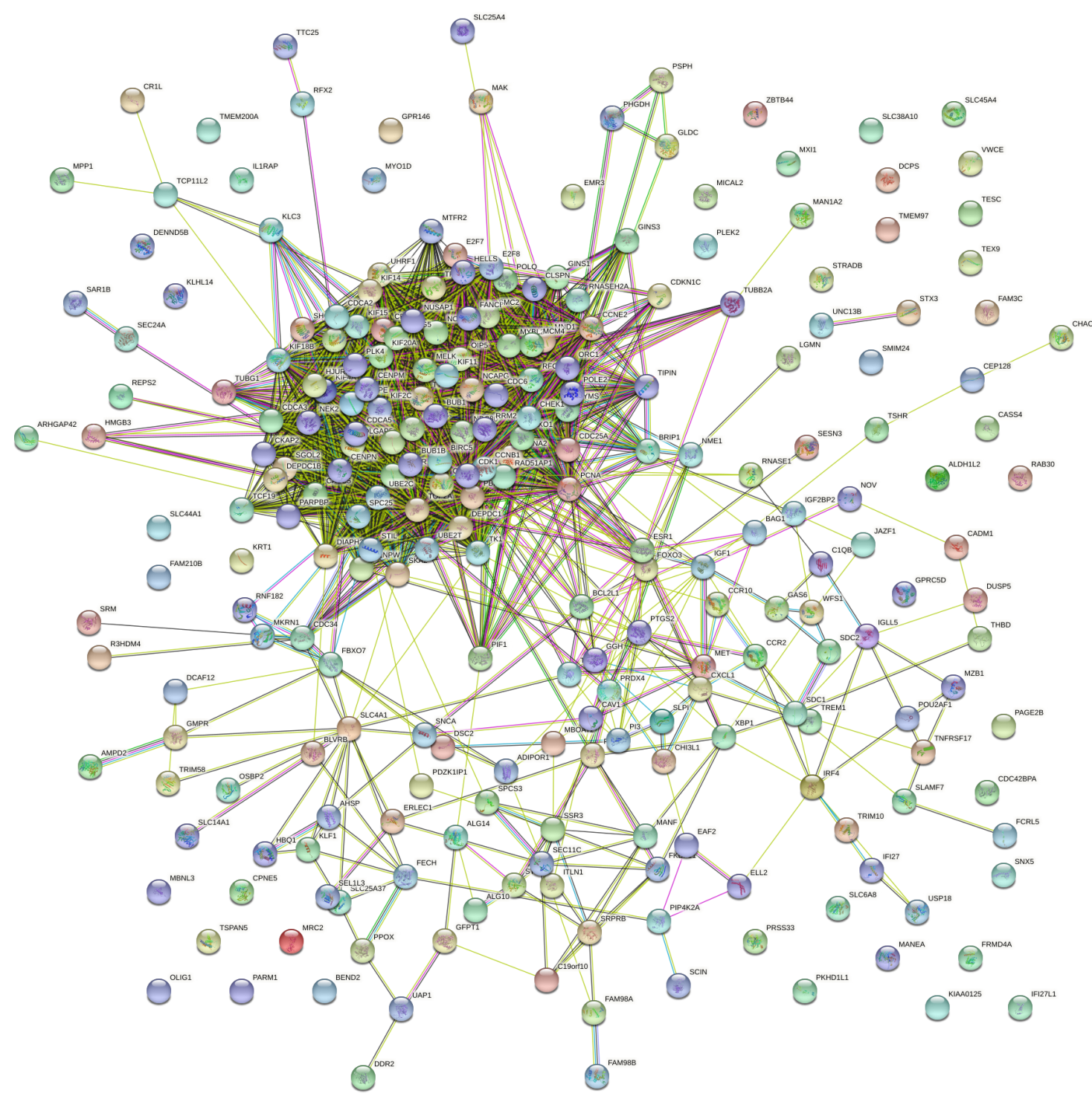


Figure-S11: STRING protein-protein interaction networks for the characterization of DEGs in DF-CO, SD-CO, CP-DF & CP-SD.


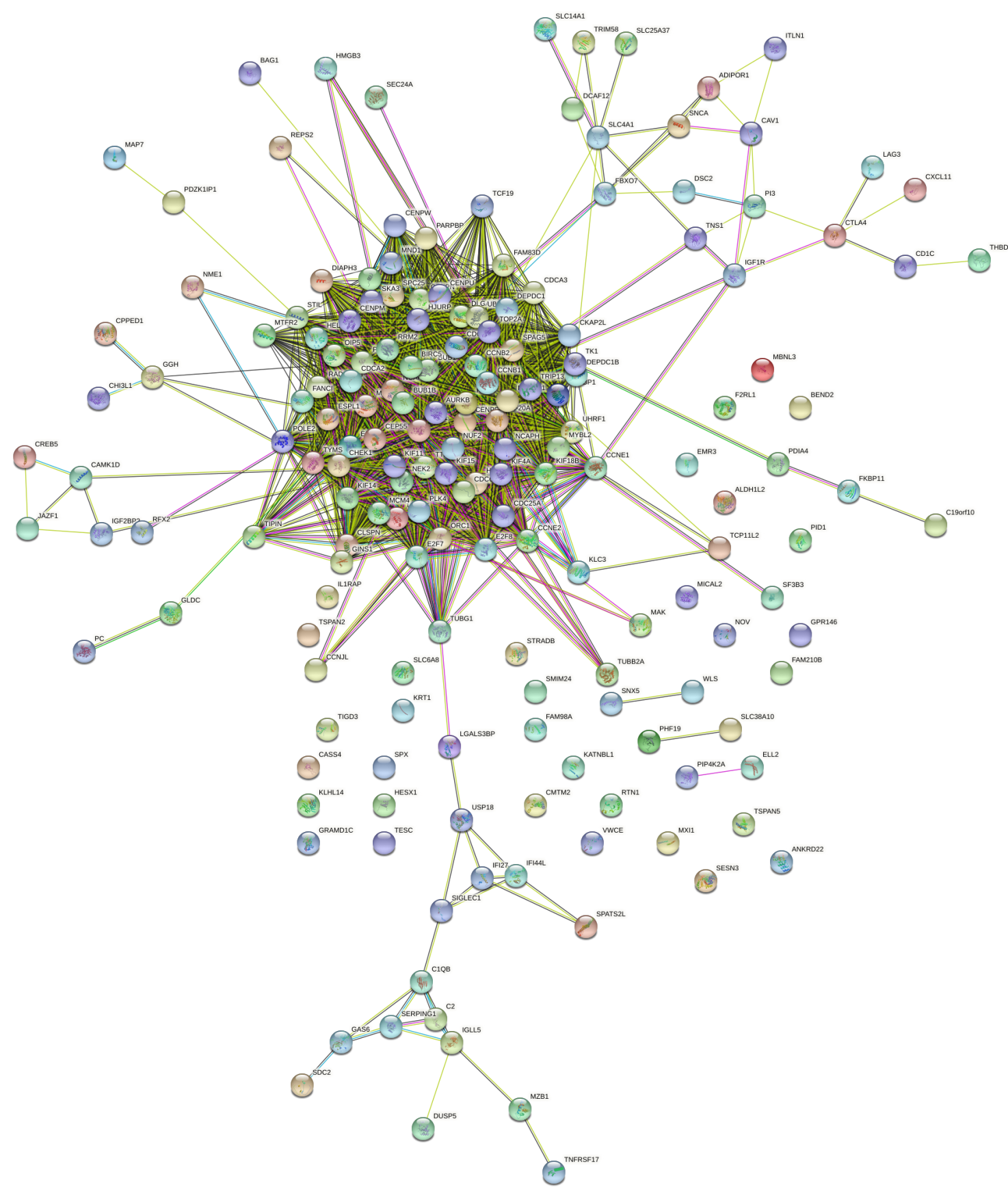


Figure-S12: STRING protein-protein interaction networks for the characterization of DEGs in DF-CO, SD-CO, CP-DF & CP-SD.
